# DNA damage and Repair Modify DNA methylation and Chromatin Domain of the Targeted Locus: Mechanism of allele methylation polymorphism

**DOI:** 10.1038/srep33222

**Published:** 2016-09-15

**Authors:** Giusi Russo, Rosaria Landi, Antonio Pezone, Annalisa Morano, Candida Zuchegna, Antonella Romano, Mark T. Muller, Max E. Gottesman, Antonio Porcellini, Enrico V. Avvedimento

**Affiliations:** 1 Dipartimento di Medicina Molecolare e Biotecnologie Mediche, Istituto di Endocrinologia ed Oncologia Sperimentale del C.N.R., Università Federico II, 80131 Napoli, Italy; 2 Dipartimento di Biologia, Università Federico II, 80126 Napoli, Italy; 3 Epigenetics Division, TopoGEN, Inc., 27960 CR319, Buena Vista, Colorado, 81211, USA; 4 Institute of Cancer Research, Columbia University Medical Center, New York, New York, 10032, USA

## Abstract

We characterize the changes in chromatin structure, DNA methylation and transcription during and after homologous DNA repair (HR). We find that HR modifies the DNA methylation pattern of the repaired segment. HR also alters local histone H3 methylation as well chromatin structure by inducing DNA-chromatin loops connecting the 5′ and 3′ ends of the repaired gene. During a two-week period after repair, transcription-associated demethylation promoted by Base Excision Repair enzymes further modifies methylation of the repaired DNA. Subsequently, the repaired genes display stable but diverse methylation profiles. These profiles govern the levels of expression in each clone. Our data argue that DNA methylation and chromatin remodelling induced by HR may be a source of permanent variation of gene expression in somatic cells.

DNA methylation in somatic cells is associated with aging, chromatin changes and efficiency of transcription^1^,^2^. There are two types of DNA methylation: 1. A stable and invariant form – imprinting - which is sex-specific and identical in individuals and cells^3^; and, 2. A metastable somatic type that changes with age and differs among individuals and cells^4^,^5^.

We have used a system pioneered by M. Jasin, in which a double-strand break (DSB) in a GFP gene generated by the meganuclease I-SceI is repaired by gene conversion from a second copy of the gene^6^,^7^. We, and others, have shown that DNA damage and homology-directed repair (HR) induce *de novo* methylation of the repaired segment. This methylation pattern is stably transmitted to daughter cells^8^,^9^,^10^. In the absence of selection, such as a neutral gene like GFP, the distribution of differentially methylated clones in the population is essentially random. We find two populations of cell clones, those that express high levels of GFP and clones that express low levels of GFP, referred to as Rec H and Rec L clones, respectively. Relative to the parental gene, the repaired GFP is hypomethylated in Rec H clones and hypermethylated in Rec L clones. The altered methylation pattern is largely restricted to a segment immediately 3′ to the DSB along the direction of transcription. Hypermethylation of this tract significantly modifies the local chromatin structure and reduces transcription^8^,^11^. These data nicely account for the high polymorphism of methylation profiles in cells populations derived from individual somatic tissues^12^. However, genome-wide methylation analysis suggests that different mechanisms may explain the loss or gain of methylation: 1. Stochastic processes may produce the high rate of methylation polymorphism; 2. Deterministic events may contribute to the gain or loss of methylation at specific loci. Moreover, changes in DNA methylation are strictly associated with post-translational modifications (PTMs) of histones. It is unclear if histone PTM variations drive or are induced by local DNA methylation. These events could generate cells with the same genotype but with various levels of gene expression.

We address the following three questions in this paper. First, what is the relationship between chromatin changes and DNA methylation at the site of repair. Second, what is the origin of the polymorphism of somatic DNA methylation. Third, does, in fact, the extent and pattern of methylation following repair impart variation of gene expression in cell populations with an identical genotype? We chose to approach these questions in a system in which DNA damage and repair can be controlled temporally and spatially, focusing our attention on local transient as well as permanent changes induced by damage and repair.

## Results

### Spatial and temporal changes of the histone H3 methylation code after homologous repair of a DSB at the GFP locus

#### The DRGFP system

The critical features of the system we use to study repair and methylation can be summarized as follows. A reporter construct (Direct-Repeat GFP: DRGFP) is randomly integrated in the genome of Hela cells at an average copy number of 1. I-SceI induces a double-strand break (DSB) in one GFP copy (I cassette) that can be repaired from the second copy (II cassette) by homologous recombination (HR), yielding GFP^+^ clones. 75–90% of the cells are repaired by NHEJ with or without small deletions at the *I-SceI* site^6^,^13^. Importantly, GFP^+^ cells can arise in this system only by HR^6^. I-SceI expression starts 2 h after transfection with an I-SceI plasmid, peaks at 24 h and slowly decays up to 48 h. At 48 h total HR, measured by qPCR, is approx. 5.0 to 10%. Cells exposed to I-SceI but which are not GFP^+^ are termed UnRec (unrecombinant). GFP^+^ cells fall into two expression classes: high and low expressers, Rec H and Rec L, respectively.

#### Transient histone H3 methylation changes induced by a DSB

**Figure 1:**
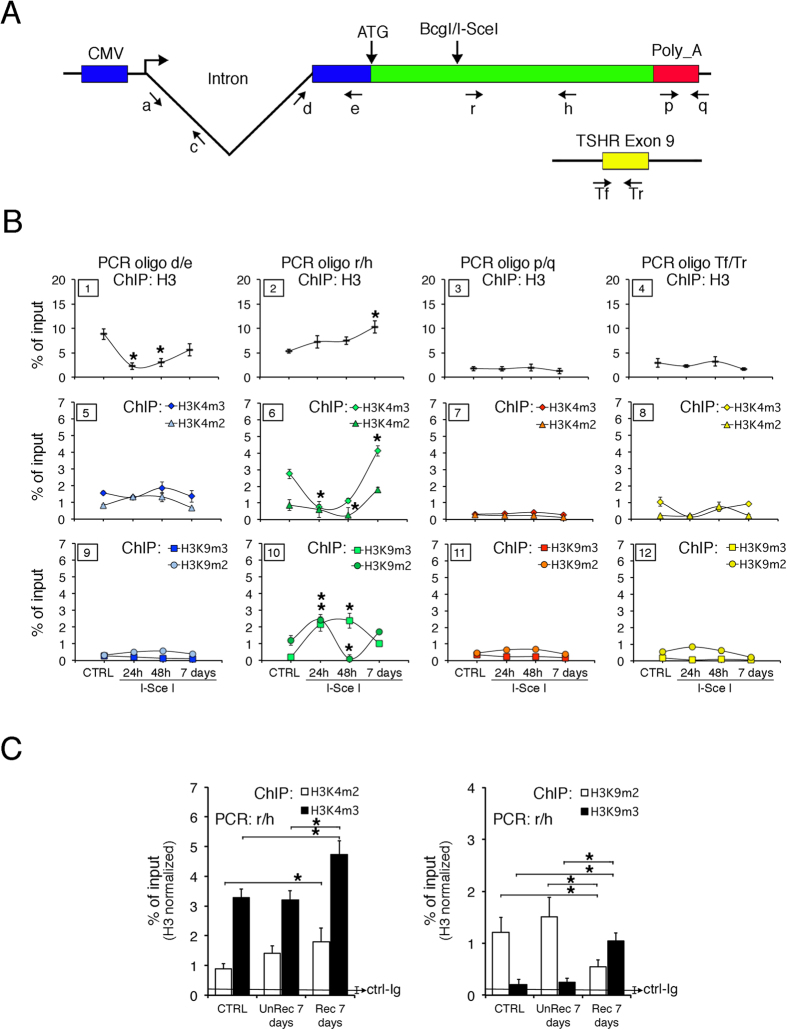
Spatial and temporal changes of histone H3 PTM after DSB at the GFP locus. (**A**) Schematic diagram of DRGFP plasmid and a reference locus (TSHR exon 9). Structure of the DRGFP plasmid integrated as single copy in different locations in Hela cells. Primers a and c cannot be used in cells transiently transfected with I-SceI plasmid because they are also present in intron 1 of the I-SceI expression vector. (**B**) H3K4m2/3 and H3K9m2/3 content of GFP and the reference gene. DRGFP cells were transfected with I-SceI and characterized 24 h, 48 h and 7 days later. Cells were fixed and the chromatin analyzed by ChIP with the indicated antibodies. qPCR on each immunoprecipitate was carried out with the primers indicated in A. The specific antibodies are indicated at the top of each column. Each panel is identified by a numbered box in the upper left side.*P < 0.01 (*t test*) as compared with untreated control or basal. (**C**) H3K4m2/3 and H3K9m2/3 content in cells sorted 7 days after transfection. CTRL are cells transfected with a control plasmid; UnRec were GFP^−^ cells sorted and separated from GFP^+^ Rec cells after I-SceI transfection. *P < 0.01 (*t test*) as compared with UnRec or CTRL. The detailed statistical analysis of the data shown in panels (B,C) is reported in Supplemental Statistical Tables 1 and 2.

Our previous work showed that levels of GFP expression reflected local DNA methylation patterns at the repaired *I-SceI* site^8^. To determine if these patterns were associated with changes in chromatin methylation, we probed the histone H3 PTMs, methylation of lysines 4 and 9, at the DSB and flanking sites 24 h, 48 h and 7 days after I-SceI expression. Chromatin was immune-precipitated with antibodies recognizing H3, H3K4m2/3 (an activation marker) or H3K9m2/3 (a repression marker). The DNA was amplified with primers corresponding to several sites upstream or downstream of the DSB along with a reference gene, exon 9 of TSHR (Fig. 1A). ChIP experiments were repeated three times in triplicates (see Supplemental Statistical Tables).

Major alterations in chromatin modification (loss of H3K4me2/3 and gain of H3K9me2/3) appeared just 3′ to the DSB 24 h after the onset of I-SceI expression: These changes disappeared 7 days later, when I-SceI levels decayed (Fig. 1B, panels 6 and 10; and ref. 11). Histone H3 decreased transiently 5′ to the DSB, but there were few other changes in the histone methylation pattern in this segment (panels 5 and 9). The H3K9 or H3K4 methylation patterns at the PolyA addition site (panels 7 and 11) or within the control TSHR gene (panels 8 and 12) were unaltered.

The major fraction of GFP ^+^ cells did not appear until 48 h. Since loss of H3K4me2/3 was detectable as early as 24 h post transfection, is likely that loss is induced by the DSB itself or by NHEJ repair of the DSB. These local histone methylation changes did not involve nucleosome or histone eviction, since total H3 content did not change significantly at the *I-SceI* site (Fig. 1B, panel 2).

#### Dynamics of histone H3 methylation changes after DNA repair

**Figure 2:**
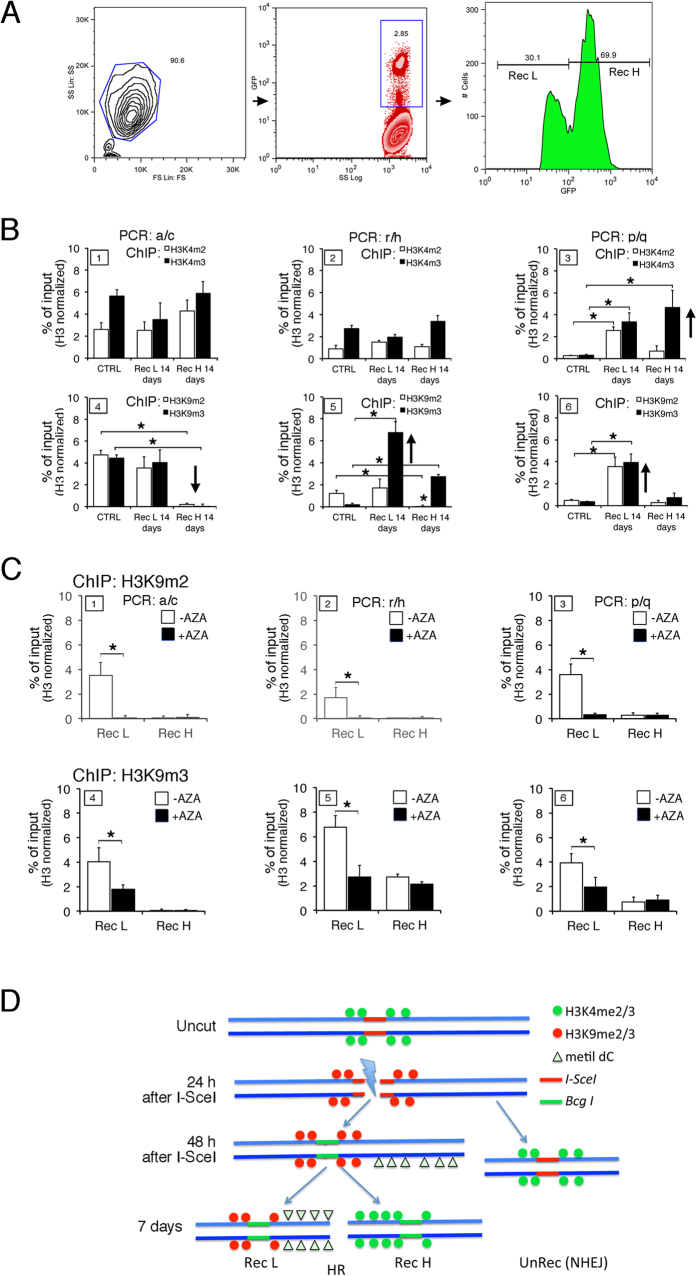
Spatial and temporal changes of histone H3 K4-K9 methylation after homologous repair at the GFP locus in HR cells. (**A**) DRGFP Hela cells were transfected with the I-SceI vector and sorted for GFP expression 14 days later as described in Methods. The panels from the left to the right show the gating strategy used to sort Rec H and Rec L cells. The percent of viable cells was 90.6. The fraction of the total GFP^+^ cells (middle panel) or of Rec H and Rec L (left panel) is indicated. (**B**) H3K4m2/3 and H3K9m2/3 levels at GFP chromatin in purified Rec H and Rec L cells, 14 days after I-SceI transfection. The specific primers are indicated at the top of each column as shown in Fig. 1A. The data are normalized to total H3 content. *P < 0.01 (*t test*) as compared with cells transfected with control plasmid (CTRL). (**C**) H3K9m2/3 content of GFP in chromatin derived from sorted Rec H or Rec L clones, untreated or treated with 5-azadC (10 μM for 3 days and analyzed 4 days later). ChIP analysis was performed with the indicated antibodies. qPCR on each immunoprecipitate was carried out with primers r/h, as shown in Fig. 1A. *p < 0.01 (*Wilcoxon rank-sum test*) as compared with untreated control or (primers a/c). (**D**) Histone H3 and DNA methylation changes following the DSB and HR, A schematic cartoon illustrating the major chromatin changes of H3K4me2/3 or H3K9me2/3 and DNA methylation (Δ) following the DSB and the HR or NHEJ in recombinant (HR) and non recombinant (NHEJ) cells. The detailed statistical analysis of the data shown in panels (B,C) is reported in Supplemental Statistical Tables 3 and 4.

At 7 days post-HR, we analysed H3K4me2/3 (Fig. 1C) and found no clear change in status in control cells (no DSB), cells subjected to I-SceI, sorted GFP^−^ (UnRec) and HR cells, GFP^+^ (Rec). We did observe, however that H3K9me2/3 marks appeared to be selectively modified in HR processed DSBs (Fig. 1C). To explore these changes in H3K9 methylation, we sorted the high GFP^+^ (Rec H) and the low (Rec L) GFP^+^-expressing cells, which had been repaired by HR (Fig. 2A) and analysed the histone modification at various sites using primers in Fig. 1A. This analysis was performed at 14 days post I-SceI exposure (a time when there was no further HR). This analysis yielded the following results. First, we saw little change in H3K4me2/3 levels in the intron 5′ to the DSB (primers a/c) when we compared control cells to Rec H or Rec L cells (Fig. 2B, panel 1). Second, H3K9me2/3 levels in Rec H cells were significantly reduced relative to control and Rec L cells (Fig. 2B panel 4). Third, the region immediately 3′ to the *I-SceI* site (primers r/h) was enriched in H3K4me3 in Rec H cells and in H3K9me3 in Rec L cells (Fig. 2B, panel 2 and 5). Fourth, GFP^+^ cells (both Rec H and Rec L) were strikingly different from control cells at the polyA addition site (primers p/q). Specifically, Rec H cells were characterized by high H3K4me3 levels (Fig. 2B, panel 3) whereas Rec L cells showed elevated H3K9me2/3 levels (Fig. 2B, panel 6). These changes were detectable in the population of HR cells but not in the mass, unsorted, population (Fig. 1B, panels 7 and 11, and 1C). From these data, we conclude that chromatin corresponding to the repaired GFP gene in recombinant GFP^+^ cells showed significant localized changes of histone H3 K4-K9 methylation markers compared to control cells. H3K9me3 was elevated at the 3′ end of the DSB in Rec L cells, and depleted at the 5′ end of the DSB in Rec H cells. Conversely, H3K4me3 accumulated mainly at the polyA site in Rec H cells. Loss of the repressive H3K9me2/3 marks at the promoter region in Rec H cells and the dramatic increase in H3K9me3 just 3′ to the repaired DSB in Rec L cells are related to high and low GFP mRNA levels in this region in H and L clones, respectively^8^.

The changes in the methylation represent a permanent, HR-associated, modification in Rec L cells. Recall that Rec L cells acquired new methylated CpGs at the DNA region immediately 3′ to the DSB after recombination, consistent with the idea that histone and DNA methylation are causally related.

#### DNA Methylation stabilizes the H3K9m2/3 marker

Why does the elevated H3K9m3 mark persist in Rec L cells? We hypothesized that HR-induced methylation of repaired DNA may maintain H3K9m3 at the repaired site. To test this idea, we de-methylated DNA in Rec L cells with 5-azadC and measured H3K9m2/3 content at three locations in the GFP locus (Fig. 2C; primers defined in Fig. 1A). We found that 5-azadC significantly reduced H3K9m2 and H3K9m3 content in Rec L cells throughout the gene (Fig. 2C). Thus, DNA methylation at the repaired GFP locus is required to maintain chromatin H3K9 methylation within the region. Taken together, these data suggest that the extensive, rapid, localized and transient increase of H3K9m2/3 is induced by DSB formation in all treated cells (Fig. 1B, panel 10). Seven days later (after most of the breaks are repaired), this mark largely disappears and is replaced by the activation marker H3K4m2/3. In the low-expressing Rec L fraction, in which GFP DNA is hypermethylated following HR, the repressive H3K9m2/3 marker remains at the *I-SceI* site. A schematic model of the histone methylation changes induced by the DSB and HR is shown in Fig. 2D.

#### Chromatin looping induced by damage and repair

**Figure 3:**
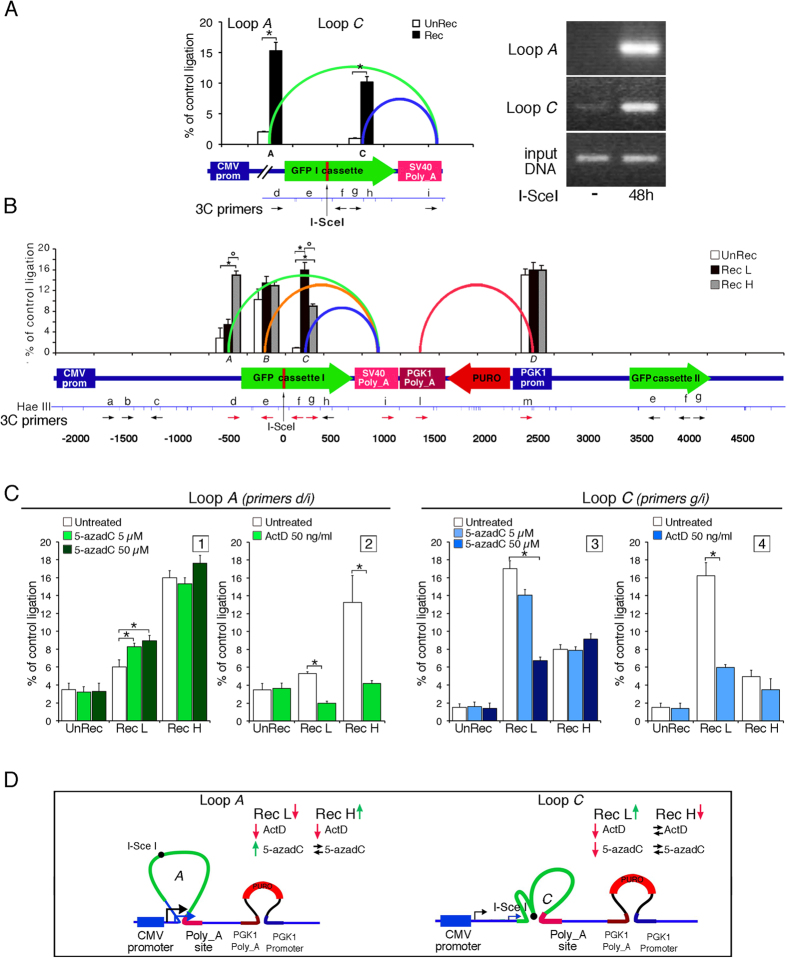
Chromatin-DNA domains induced by damage-repair. (**A**) **Left.** GFP chromatin loops in recombinant (Rec, black) and unrecombinant (UnRec, white) cells 7 days after DSB. qPCR in 3C, performed with the primers indicated (HaeIII map), show the frequency of ligation (mean ± SD) of the DRGFP HaeIII fragments amplified with the specific primers. The ends of the loops are shown by the green (loop A) and blue (loop C) lines, corresponding to the regions indicated by arrows and boxes. Ligation efficiency is relative to the DRGFP plasmid digested with HaeIII, ligated and amplified by qPCR. Distance, in bp, is relative to *I-SceI* site (vertical arrow), *p < 0.01 (*t test*) Rec *vs* UnRec. **Right.** A representative gel of the ligated fragments (loops A and C) in cells exposed or not for 48 h to I-SceI. (**B**) GFP loops in Rec H and Rec L cells. The 5′ end of the loop A (green) includes an alternative GFP transcription start site, identified by primer *d*. Differences between non-recombinant, H or L cells *p < 0.01 (*t test*); °p < 0.01 (*t test*) H vs L cells. (**C**) Inhibition of methylation (panels 1, 3) or transcription (panels 2, 4) alters the chromatin loops induced by HR. **Panels 1 and 3.** Sorted GFP^−^ (UnRec) or Rec H or Rec L cells were exposed to 5 or 50 μM 5-azadC for 24 h + 24 h in standard medium. Loops A and C were monitored as described above. The results derive from at least 3 experiments in triplicate. *p < 0.01 as compared to untreated samples (*Wilcoxon rank-sum test*). **Panels 2 and 4.** Sorted GFP^−^ (UnRec) or Rec H or Rec L cells were exposed to 5 μM actinomycin D for 24 h, washed and analysed 48 h later. *p < 0.01 (*Wilcoxon rank-sum test*) compared to untreated cells. (**D**) Model summarizing the features of Loops A and C. The red and green arrows indicate decrease or increase of loop formation, respectively, while the black arrows indicate no change. The two GFP transcription start sites are indicated by black and blue arrows.

We suggest that *de novo* DNA methylation at the site of DSB repair stabilizes the H3K9m3 mark, which alters the chromatin structure of the entire GFP gene. To map specific domains modified by DNA damage and repair, we examined the structure of chromatin at the repaired locus in UnRec (repaired by NHEJ) and Rec cells by chromosome conformation capture (3C). Among the primers used (Fig. 3A,B), only a few were able to amplify specific DNA fragments. PCR and sequence analysis showed these segments to be contiguous in the chromatin but not in the DNA. Two looped segments are shown in Fig. 3A. Loops *A* and *C* are specific to recombinant cells and mark different regions of GFP cassette I at the 5′ end relative to the *I*-*SceI* site: loop *A* links a region that includes a GFP transcription start site driven by the chicken β-actin promoter (5′ end, identified by primer *d*, from −517 to −279; green loop). Loop *C* includes a more distal 3′ region of the GFP coding sequence located downstream to the *I-SceI* site (5′ end identified by primers *f*, *g*, from +70 to +300; blue loop). Figure 3A, right panel, shows that 48 h after I-SceI expression, at which time repair was complete, both loops were detectable in the mass culture. Thus, these loops form soon after the repair process. Figure 3B shows the chromatin loops detected in Rec H and Rec L sorted cells after I-SceI transfection. In addition to loops A and C, two other chromatin loops juxtapose different elements of the DRGFP insert. One loop connects the PGK1-PolyA addition site of the puromycin acetyl transferase gene with its promoter (loop D in red, Fig. 3B). This loop, present both before and after DNA damage and repair, marks the border of the puromycin acetyl transferase gene transcription unit, and serves as an internal positive control. Loop B, present in both recombinant and UnRec cells, serves as an additional internal control (Fig. 3B, orange loop, 5′ end identified by primer *e*). Loops A and C mark selectively recombinant cells as shown in Fig. 3A. Specifically, the frequency of loop A is high in Rec H and low in Rec L cells, whereas loop *C* abundance was the converse, low in Rec H and high in Rec L cells. Note that the 5′ end of loop C corresponds to the segment of GFP that is *de novo* methylated after repair. It is possible that local DNA methylation influences the formation or stability of loop C in L cells. Consistent with this notion, Rec L cells exposed to 5-azadC (and consequently hypomethylated) for 72 h formed less loop C and more loop A. Thus, Rec L cells may convert to Rec H cells following loss of DNA methylation (Fig. 3C, panels 1 and 3). These data suggest that these chromatin loops are related to the transcription efficiency of the repaired gene. To clarify this point, we first determined RNA polymerase II (Pol II) occupancy by ChIP analysis at the GFP promoter, the translation start site, the DSB region and the 3′ polyA site (Supplemental Fig. S1A). Pol II concentrations were highest at the GFP promoter and polyA sites in non-recombinant or Rec H cells. Pol II occupancy was reduced at all sites in Rec L cells relative to UnRec or Rec H cells. Second, we inhibited transcription with low doses of actinomycin D and monitored formation of loops A and C. Actinomycin D reduced the abundance of loop A in both Rec H and Rec L cells and loop *C* in Rec L cells (Fig. 3C, panels 2 and 4), suggesting that both loops are either generated or maintained by transcription. The results of these experiments are summarized in Fig. 3D.

In summary, DNA repair and associated transcription permanently modify the structure of local chromatin, generating chromatin loops that juxtapose the 3′ end of the transcribed gene with various 5′ sites. These structures reflect the transcriptional status of the gene and are influenced by local DNA methylation.

### BER enzymes remodel DNA methylation soon after HR

**Figure 4:**
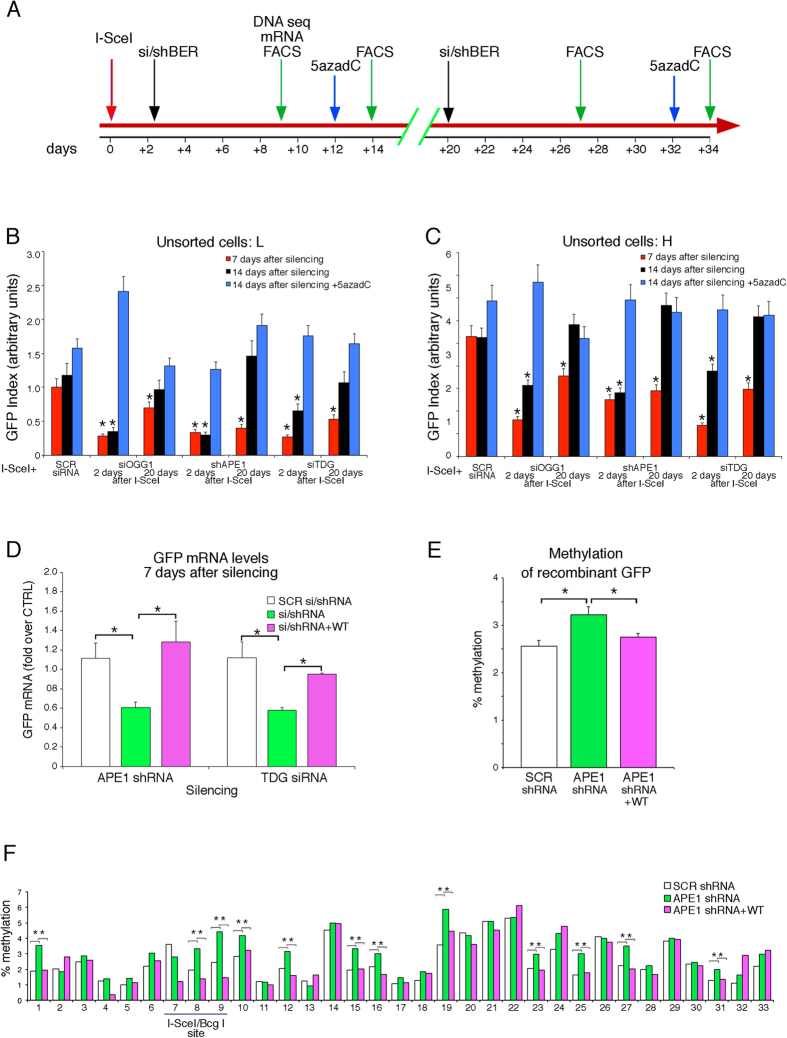
Inhibition of BER early after repair reduces transcription and increases methylation of the repaired DNA. (**A**) Chronology of BER-silencing experiments. Time 0 indicates I-SceI transfection and arrows the time of analysis or treatments. GFP^+^ cells were 10% ± 2% in all treatments. At days 12 and 32, 5-azadC (10 μg/ml, blue arrows) was added for 24 h, removed and 24 h later cells were analyzed. (**B**,**C**) Analysis was performed at day 7 (red) or 14 (black) after treatments. GFP index is the product of GFP intensity and reciprocal cell fraction in the fluorescence gate to normalize frequency of GFP^+^ cells in H and L gates to intensity of signal, to compare different experiments. The data shown derive from 20 independent experiments. (**B**,**C)** Show GFP index in Rec H and Rec L cells, respectively. *p < 0.01 (*Matched t test*) compared to scrambled control. (Supplemental Fig. S3 and Table S2). (**D**) GFP mRNA levels 7 days after APE1 and TDG-silencing by qPCR for recombinant GFP. TDG protein levels are in Supplemental Fig. S3C. *P < 0.01 (*Wilcoxon rank-sum test*) compared to scrambled control. (**E,F)** Methylation analysis of GFP in mass cultures of I-SceI-transfected cells, in which APE1 levels were modified after HR (48 h after I-SceI and analyzed 7 days later, panel A). DNA was subjected to bisulfite analysis and sequenced with Myseq Illumina (Supplemental Table S3). Panel E shows the average methylation of the recombinant GFP. All the cells were exposed to I-SceI and these vectors: SCR shRNA (white shaded) scrambled shRNA; APE1 shRNA (green) shRNA APE1; APE1 shRNA + WT (purple) shRNA APE1 and APE1 expression vector. The percent of methylation of recombinant GFP in all samples is normalized to the recombinant GFP cassette as shown in Supplemental Table S3; data were expressed as the mean ± SEM. *p < 0.01 (*t test*) compared APE1 shRNA *vs* SCR shRNA or *vs* APE1 shRNA + WT. Panel F shows the percent of CpG methylation in recombinant GFP in cells in which the levels of APE1 were modified early after repair. The position of the *I-SceI/BcgI* site corresponds to CpG 7 and 8. *p < 0.01 (*Pearson’s chi-squared test*) compared APE1 shRNA *vs* SCR shRNA (+SceI) or *vs* APE1 shRNA + WT.

We previously reported that GFP DNA methylation in HR cells was modified by transcription after repair. Thus, inhibiting transcription with short pulses of actinomycin D shortly after HR permanently increased DNA methylation of the repaired GFP gene^11^. We hypothesized that transcription was associated with active demethylation^14^,^15^. To test this idea, we focused on BER enzymes, which promote DNA oxidation and cytosine demethylation during transcription^16^,^17^,^18^. We first asked if APE1, the BER apurinic site nuclease, was recruited to GFP chromatin before or after repair. Indeed, APE1 was enriched mainly at the promoter site of all cell types, but was specifically enriched at the 3′ end of the DSB in Rec H and Rec L cells (Supplemental Fig. S1B). We note that APE1 was present at high levels along the entire gene in Rec H cells, suggesting a role for APE1 in the transcription of the repaired gene (Supplemental Fig. S1B). We have previously shown that OGG1, the 8-oxoG glycosylase and APE1 are important in Myc^17^, estrogen-16 and retinoic acid-18 induced transcription. We hypothesized that BER enzymes may control the rate of cytosine demethylation during transcription of the repaired gene in a precise time frame after repair. To confirm and define this critical period necessary to establish permanent DNA methylation changes, we depleted BER enzymes at 2 or 20 days after DSB formation (Fig. 4A). We selectively inhibited APE1 and two other BER enzymes, OGG1, and TDG (Supplemental Figs S2 and S3). TDG has also been directly implicated in active DNA demethylation^19^,^20^,^21^.

The effects on GFP expression were evaluated beginning 7 and 14 days after silencing, when the mRNA and protein levels expressed by the targeted genes had returned to normal (Supplemental Fig. S2C). The timing of the knock-downs relative to the formation of the DSBs is shown in Fig. 4A. Figure 4B,C display the effects of silencing OGG1, APE1 and TDG on GFP expression as assayed by cytofluorimetry. GFP expression is represented by a GFP index, which takes into account the distribution of Rec H and Rec L cell populations (% of Rec H and Rec L peaks) and the fluorescence intensity (see legend of Fig. 4). Silencing was initiated at 2 or 20 days after DSB formation and Rec H and Rec L peak percentages and fluorescence intensity were measured. The color code of the histograms shown in Fig. 4B,C indicates the time of analysis after initiation of silencing (7 and 14 days, red bars and black bars, respectively), and the effects of treating the samples with 5-azadC on GFP expression (blue bars). Since HR is complete by 2 days following exposure to I-SceI, BER depletion at or after this time had no effect on the frequency of GFP^+^ cells (Supplemental Figs S2 and S3), although it significantly altered the levels of GFP expression (Fig. 4B–E).

Figure 4B,C show that silencing of BER enzymes early after HR permanently inhibited GFP expression in both Rec H and Rec L cell populations, even when the concentration of the depleted proteins returned to pre-treatment levels (Supplemental Fig. S2C). Inhibition of GFP expression was due to specific reduction of APE1 and TDG mRNA, since it was reversed with plasmids expressing APE1 and TDG (Fig. 4D). APE1 silencing resulted in an elevation of DNA methylation (Fig. 4E) and consistent with this observation, 5-azadC restored GFP expression to normal levels in BER-depleted cells (Fig. 4B,C, blue bars). Depletion of the BER enzymes 20 days after DSB formation did not modify GFP methylation or expression (Fig. 4B,C). To gain insight into the methylation status of repaired GFP in cells in which APE1 levels were manipulated early after repair, we performed deep sequencing of bisulfite-treated DNA derived from of mass cultures (data set are available at Figshare; DOI:10.6084/m9.figshare.3470099). We analysed at least 8,000 GFP molecules/samples (Supplemental Table S3) and measured total methylation of GFP in cells in which APE1 levels were down-regulated after repair. Total methylation did not differ significantly between recombinant and unrecombinant or control cells, because HR cells include both hypo- (H) and hyper-methylated (L) clones. However, GFP methylation increased in cells in which APE1 had been depleted early after repair. Reconstitution of APE1 eliminated this increase and restored methylation to control (scrambled shRNA) levels (Fig. 4E).

We next analysed the methylation status of individual CpGs in the HR region. We found that APE1 levels significantly affected the methylation pattern.

The percentage of methylation of each CpG, (numbered from 1 to 33), is tabulated within the *I-SceI* region. APE1 depletion stimulated methylation at a subset of these sites 1, 8, 9, 10, 12, 15, 16, 19, 23, 25, 27, 31. The methylation gain at the majority of these sites was eliminated when APE1 levels were restored by expressing the wild-type protein from an expression vector (*).

In conclusion, the data shown in Fig. 4F provide an extensive window on the methylation changes that follow repair. Several sites, which we call seeds, are preferentially methylated and demethylated after HR, suggesting that the DNA methylation status early after repair is subject to extensive remodelling by transcription and BER-associated demethylation.

### Discrete DNA methylation patterns mark clones with distinct GFP gene expression levels

DNA methylation status is highly polymorphic and can be reshaped during and after DNA damage-repair events. Over time, the DNA methylation profiles of Rec H and Rec L cells stabilize and generate cells with different but heritable GFP expression levels. These clones are characterized by specific GFP chromatin domain patterns (Figs 1,2 and 3).

**Figure 5:**
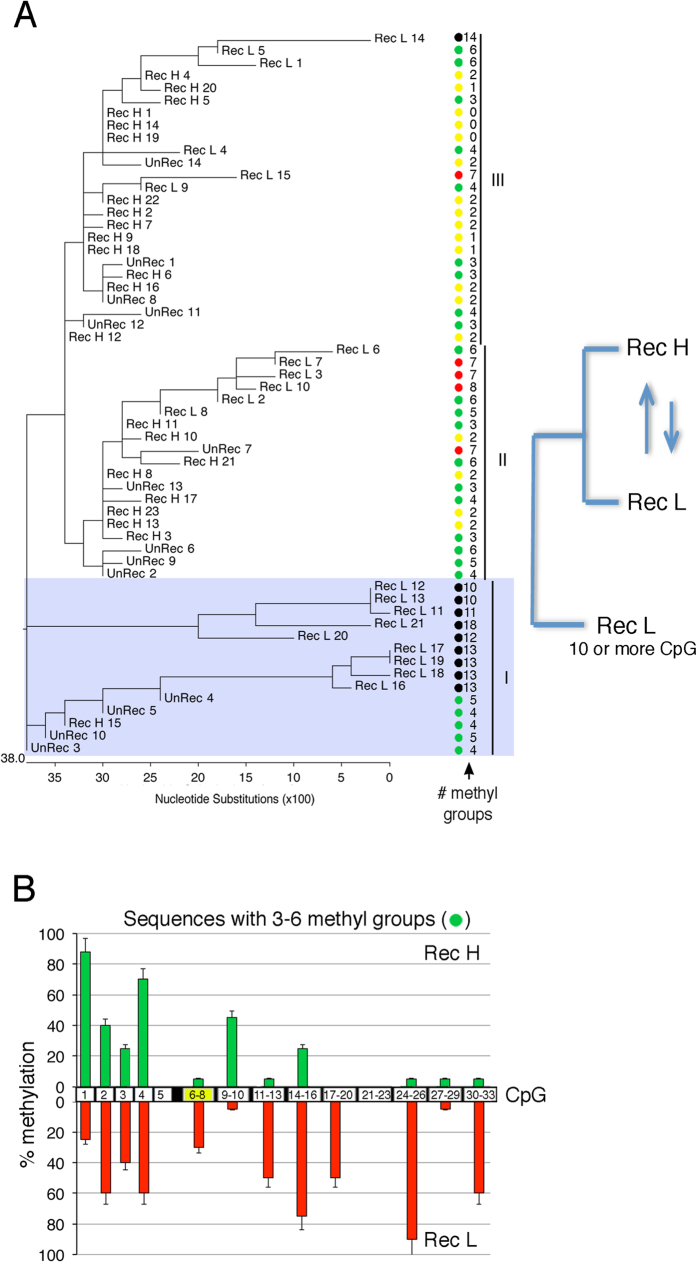
Methylated GFP molecules are polymorphic. (**A**) Qualitative analysis of GFP methylation profiles in UnRec, Rec H and Rec L clones. We compared the location of methylated CpGs in the most abundant GFP molecules (above 5–10%) derived from recombinant (Rec H and Rec L) and non-recombinant (UnRec,) cells. The sequence at the *I-SceI* site in UnRec molecules was edited to *BcgI* to eliminate the differences in the sequence between Rec and UnRec molecules and to permit the comparison of Rec and UnRec GFP molecules only on the basis of methylation. Cluster analysis (ClustalW) shows three main families of methylated molecules: I, represented essentially by Rec L clones; II and III represented by Rec H and Rec L clones. Colored circles indicate the number of methylated CpGs/molecule. (**B**) Specific CpGs are methylated in Rec H and Rec L clones following HR. Molecules containing 3 to 6 mCpGs were sorted from Rec H or Rec L cells pools and compared. The location of methylated CpGs at the 5′ and 3′ ends of the DSB is shown relative to the DSB (black-yellow box centered at the 6–8 CpG). Methylation of CpG from 1 to 5 is not modified by DSB or HR (ref. 11). The histograms show the percentage of methylation of the specific CpGs in Rec H (green) and Rec L (red) clones.

To relate the extent and placement of DNA methylation with gene expression, we compared the location and the number of methylated CpGs (mCpGs) with GFP expression levels in the most frequently modified GFP molecules isolated from sorted Rec H and Rec L cells. We ordered the methylated GFP molecules into families that share mCpGs at identical locations to define epigenetic haplotypes. Specifically, we asked if molecules with the same number of mCpGs, but located at different sites in the gene (i.e., different haplotypes), expressed similar levels of GFP. We also included in this analysis the most frequent UnRec molecules to reveal potential relationships between GFP molecules present before and after HR. Figure 5A shows the similarity of the repaired GFP molecules on the basis of the position of mCpGs and GFP expression levels. There are two main branches in the tree: the first, indicated as I, includes essentially L clones with 10 or more mCpGs; the second, indicated as II and III, contains GFP molecules with intermediate frequencies of mCpGs (from 3 to 7) and includes both H and L clones. The arrows shown in the cartoon below the tree indicate that the L and H clones in these groups are very similar in terms of mCpG content. The clones with intermediate levels of mCpGs contain the same number of mCpGs but carry them in different locations. This reveals that the location of the mCpGs, rather than their absolute frequency is critical for GFP expression. We have mapped the mCpGs that characterize Rec H (green) and Rec L (red) clones. We find that some positions (for example mCpGs 9–10) are specific to Rec H clones, while others characterize Rec L clones (mCpGs 17–20; Fig. 5B). These mCpGs that characterize H and L clones were detected in a large unsorted pool of GFP^+^ molecules isolated from a mass culture of cells exposed to I-SceI (Fig. 4F). These mCpGs are stable over time and can be recovered with the same frequency after three years of continuous culture^8^,^11^.

### Homologous targeting of GFP in ES cells also generates clones with various levels of GFP expression and DNA methylation

**Figure 6:**
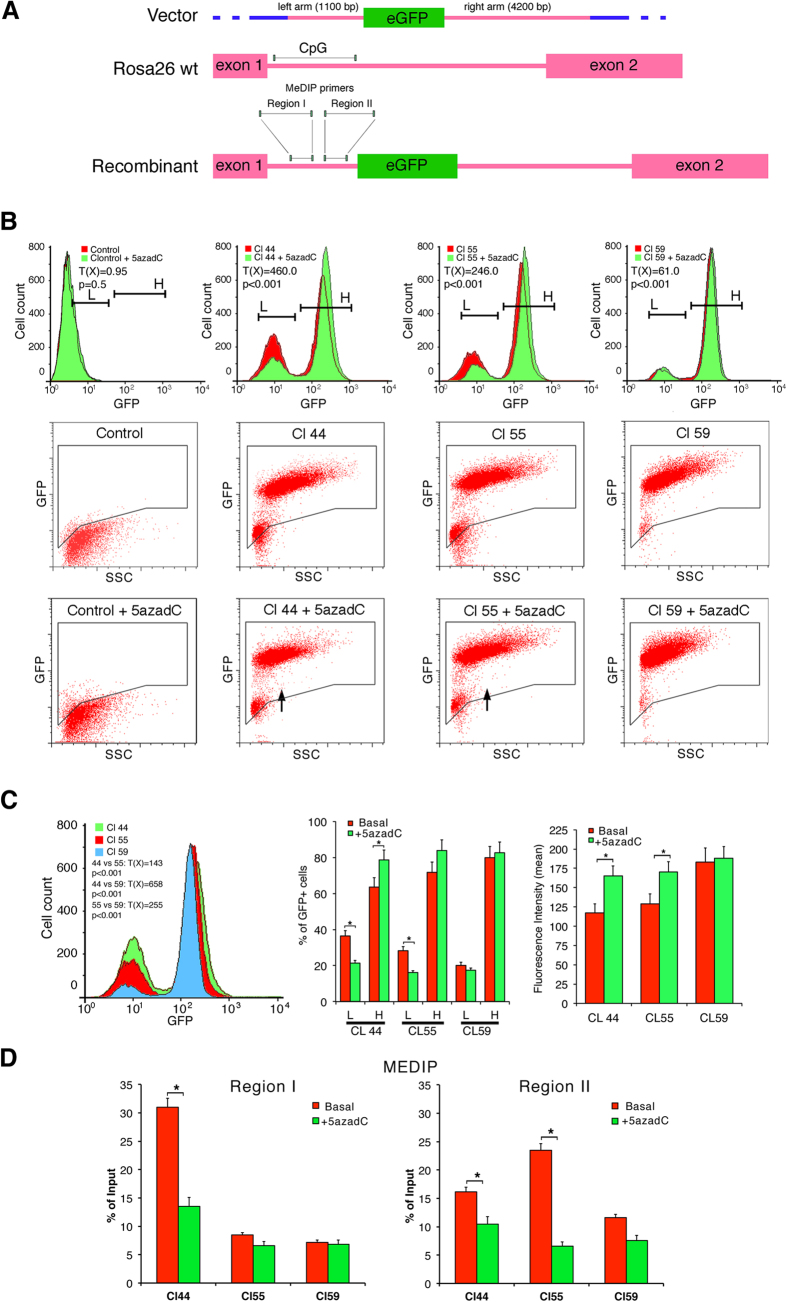
Homologous targeting of GFP to the mouse Rosa26 locus generates ES clones with variable DNA methylation and GFP expression. Three independent ES clones in which the EGFP gene was targeted to the mouse Rosa26 locus, (kindly provided by A. Simeone and D. Acampora, Intern. Inst. Gen. Biophys., IGB, Naples, Italy). These clones, containing a single copy integrated GFP, were purified 7 days after transfection with the targeting vector, amplified and analyzed by cytofluorimetry as described in Methods. The same clones were exposed to 5-azadC (0.5 μM) for 4 days and analyzed 48 h later. Panel (A) shows: 1. the structure of the targeting vector; the two homologous regions are shown in red; 2. the structure of the mouse Rosa26 locus and; 3. the structure of the targeted locus. A segment at the 5′ end, containing a CpG island, is shown (lines). The primers for MEDIP analysis are located in regions I and II. Panel (B) shows the cytofluorimetric analysis of the 3 clones exposed or not to 0.5 μM 5-azadC for 4 days and analyzed 48 h later. Dot Plot scans are shown to illustrate the composition of GFP^+^ or GFP^−^ cells. The arrows indicate the shift of the L population after 5-azadC treatment. Differences in GFP expression between control and 5-azadC treated cells were tested for statistical significance using the Chi Square test, T(X), (Population Comparison module of the FlowJo software from Tree Star). Cl 44, untreated vs 5-azadC T(X) = 460, p > 0.001; Cl 55, untreated vs 5-azadC T(X) = 246, p > 0.001; Cl 59, untreated vs 5-azadC T(X) = 61, p < 0.001. (**C**) The panel on the left shows the overlapping profiles of the three clones without treatment to compare the relative GFP expression levels. Cl 44 vs Cl55 T(X) = 143, p < 0.001; Cl 44 vs Cl59 T(X) = 658, p > 0.001; Cl 55 vs Cl59 T(X) = 255, p > 0.001. The central and the left panels show the quantitative analysis of GFP expression in Rec H and Rec L cells before or after 5-azadC treatment as % of GFP^+^ cells and mean of fluorescence intensity. In the left panel, Rec H and Rec L clones were analyzed together. Differences between treatments were tested for statistical significance using matched pairs *t* test: *p < 0.001. Panel (D) shows MEDIP analysis of region I and II, respectively in the 3 clones. *p < 0.001.

The data presented above indicate that HR repair of a DSB changes the methylation pattern of the repaired segment. In these experiments the DSB was artificially generated by the I-SceI meganuclease. To study DSBs, created by a different mechanism, we targeted CMV-GFP to a mouse DNA locus (Rosa26) by homologous recombination, which requires formation and repair of a DSB (Fig. 6A). Based on the *I-SceI* data, we predicted that the expression level of the inserted gene would differ in genetically identical clones. We isolated 3 mouse ES clones carrying a single copy of CMV-GFP targeted to the Rosa26 locus (A. Simeone and D. Acampora, unpublished observations and Fig. 6A). These clones, 44, 55 and 59, were characterized for GFP expression. Clones 44 and 55 contain two populations that differ in GFP expression levels, whereas clone 59 contains primarily cells that express high GFP levels (Fig. 6B). We propose that the distinct progeny of clones 44 and 55 are equivalent to the hypomethylated Rec H and hypermethylated Rec L clones found in DRGFP HeLa cells, and that clone 59 only generated Rec H progeny. To test this hypothesis, we treated the cells with 5-azadC and measured GFP expression. Exposure to 5-azadC shifted Rec H cells to the right (higher expression) and reduced the number of Rec L cells in clones 44 and 55. Demethylation slightly affected GFP expression in derivatives of clone 59 (Fig. 6B,C).

We also determined the DNA methylation status of the CpG island at the 5′ end of the homologous targeting sequence with primers specific to sub-regions I and II (Fig. 6A). MEDIP analysis showed that region I in clone 44 was hypermethylated compared to clones 55 and 59. Region II in clone 55, and to a lesser extent in clone 44, was hypermethylated compared to clone 59, which is not methylated (Fig. 6D). The differences in mCpG content were largely reversed by treatment with 5-azadC (Fig. 6D). On the basis of the *I-SceI* data. we hypothesize that the methylated subregion is located at the 3′ end of the DSB, which initiates HR along the direction of transcription. We propose that the DSB occurring during homologous pairing upstream of region I generated clone 44 and that a DSB between regions I and II generated clone 55. Clone 59 is equivalent to high expressor clones found in GFP^+^ cells.

These data extend the notion of HR-induced methylation and suggest a general mechanism that modifies expression of targeted genes by homologous recombination.

## Discussion

The data reported here shed light on somatic DNA methylation and consequent histone modification induced by damage and homologous repair. They suggest that cell-to-cell variations in gene expression are dependent on the different DNA methylation profiles and chromatin structures of the expressed gene acquired during or soon after HR.

Transient and stable *cis* and *trans* chromatin changes induced by DNA damage and repair. Concurrent with DSB formation by I-SceI and repair by HR, chromatin near the lesion becomes enriched with the repressive chromatin mark, H3K9m2/3. This modification has been reported to be essential to recruit other histone-modifying enzymes and ATM to the site of damage^22^. The I-SceI-treated cells also transiently lose H3K4 methylation, but restore the H3K4m2/3 mark 2 to 7 days after exposure. The rapid appearance of H3K9me2/3 and loss of H3K4me3 at the DSB region after I-SceI exposure (Fig. 1B, panels 6, 10) suggest that these changes are induced by the formation of DSBs. Subsequent purification reveals that H3K9m2/3 is selectively retained after HR only in Rec L clones (Fig. 2A,B). Maintenance of H3K4m2/3 is secondary to DNA methylation. Thus, treatment of Rec L cells with the DNA demethylating agent, 5-azadC, significantly reduced the levels of H3K9m2/3 on GFP chromatin (Fig. 2C).

*De novo* methylation of the repaired segment was also responsible for stabilization of the chromatin loop specific to Rec L cells (loop *C* in Fig. 3). Repressive methylation H3K9m2/3 marks in Rec L cells were also present at sites physically distant to the repaired DSB. However, these sites were, in fact, juxtaposed and linked by loop *C* (Fig. 3). At other physically distant sites (e.g., the puromycin-resistance gene), histone marks were not modified by damage and repair (data not shown). Note that a similar series of events occurs during transposon integration and silencing. Chromatin repressive marks (H3K9me2/3) are induced early during integration of the transposable element (TE) followed by methylation of the integrated segment^23^.

We propose that the initial functionally relevant event in HR-directed gene modification is the formation of H3K9me3 at the DSB. This modification is induced by the DSB or by NHEJ in the majority of cells. Histone methylation is carried out by the histone methyltransferase SUV39, which is recruited to the DSB^11^,^24^,^25^. The increase of H3K9me3 contributes to the repression of local transcription induced by DNA damage^26^,^27^. The H3K9me2/3 at the DSB is progressively lost after repair except in Rec L cells, and to a lesser extent in Rec H clones (see Fig. 2B, panel 5). Rec L and Rec H clones are characterized by discrete chromatin loops (Fig. 3) and specific H3K4me2/3 and H3K9me2/3 profiles along the GFP gene (Fig. 2B). These marks appear very early after DSB formation in unsorted mass culture and precede stabilization (Fig. 3A). The most striking feature of the HR cells is the stability and the inheritance of the chromatin and DNA changes at the repaired locus. Our data also indicate that the initial *de novo* methylation of the repaired segment can be revised. Early after exposure to I-SceI, the ratio of Rec H to Rec L clones was approximately 1:1. After 7–14 days, the ratio changed to ≃4:1, depending on the clone^8^. The ratio was stabilized permanently by day 21. The repressive chromatin marks (H3K9me2/3) were maintained only in Rec L cells, suggesting that the DNA methylation profiles after repair stabilize repressive chromatin markers (Fig. 2C).

BER enzymes reshape stable methylation. Overall, the final methylation status of the repaired gene is polymorphic (Figs 4F and 5A). We find that depletion of BER enzymes two days after DSB formation modified permanently the expression of repaired GFP (Fig. 4 and Supplemental Figs S2 and S3). We, and others, have reported that recruitment of BER and NER enzymes to promoter sites of several nuclear hormones or HIF-induced genes is essential for transcription^16^,^18^,^28^,^29^. Additionally, the BER enzyme, TDG, which suppressed GFP DNA methylation after DSB repair (Supplemental Fig. S2B,C) is involved in active CpG de-methylation during transcription^19^,^20^,^21^. These data and those shown in Fig. 4 and Supplemental Figs S2 and S3 indicate that other BER enzymes, (e.g., OGG1 and APE1), which recognize and process oxidized G, are also involved in transcription-associated demethylation. Indeed, although the absolute methylation levels of the repaired segments do not change dramatically, depletion of BER enzymes significantly changes the qualitative methylation profiles of the repaired segment, 3′ to the *I-SceI* site (Fig. 4). We propose, therefore, that BER enzymes perform transcription-associated demethylation at GFP at the repaired locus. This process is partly stochastic with respect to a particular CpG, since some CpGs are preferentially methylated (Figs 4F and 5B,C). These may seed further methylation along the repaired gene. Methylation revision generates polymorphic methylation profiles, which influence GFP expression depending on the location of the methylated CpG (Fig. 5). We wish to stress that CpG preferentially methylated in sorted H or L clones were also identified in mass culture sequencing (Figs 4F and 5B).

Polymorphism of somatic methylation profiles. Comparing the levels of expression of GFP with the methylation profiles of single DNA molecules, we find a relationship between the profile and the expression of the repaired gene. We describe clones carrying the same number of mCpGs (Fig. 5), but whose GFP enzyme levels vary dramatically. The differences of GFP expression between Rec H and Rec L clones are reduced or erased by 5-azadC treatment, indicating that the variations in expression reflect the methylation status of specific CpGs.

In conclusion, we propose that the variability of GFP expression is affected by editing of local methylation by transcription and active demethylation (Fig. 4; ref. 19), which together with DNA damage and repair represent a major source of polymorphism of methylation in somatic cells.

Is DNA methylation in somatic cells deterministic or stochastic? It was recently reported that methylation of INK4-ARF suppressor gene is induced by a specific Ki-Ras oncogene transcriptional program^30^. This observation supports a general deterministic model for DNA methylation in which locus-specific targeting of DNMT enzymes induces and maintains DNA methylation. The choice of target is not random, but determined by specific affinities of transcription factors and chromatin modellers. Eventually, the preference of DNMT1 for hemi-methylated DNA stabilizes the methylation profiles. This deterministic model may rationalize clustering of methylated sites in the same DNA region, but fails short of explaining the extreme polymorphism of methylated alleles found with a deeper sequence coverage of the genome (Fig 5 and ref. 12).

Our data suggest that both deterministic and stochastic factors govern stable DNA methylation profiles. In the system described here, we can quantify the deterministic and the stochastic factors that contribute to the final methylation status of GFP epialleles. HR and specific factors, recruited to the DSB, establish the location and the strand that will be methylated (Figs 2D and 5 and refs 10,11). In contrast to this deterministic modification of the epigenome, stochastic editing of methylation by transcription and BER enzymes generates polymorphism of the methylated GFP alleles.

We propose, therefore that both mechanisms contribute to the final methylation status of DNA in each cell. Importantly, our work impacts in the area of genome editing, which is largely driven by HR. The final penetrance of a repaired gene will be directed by the events related to DNA methylation revision described here.

## Methods

### Cell culture, transfections and plasmids

HeLa cells lines were cultured at 37 °C in 5% CO_2_ in RPMI medium supplemented with 10% fetal bovine serum (Invitrogen), 1% penicillin-streptomycin, and 2 mM glutamine. HeLa-DR-GFP cells were obtained by stable transfection of HeLa cells with the pDR-GFP plasmid as described in ref. 11. We used the same conditions of growth (~40% confluent cells starting from freshly frozen aliquots). The structure of the pDR-GFP and other plasmids is described in Supplemental Information. The expression vectors for OGG1WT and for the K338R/K341R OGG1 mutant were the FLG-Tagged vectors previously described in ref. 31.

### DNA extraction and qRT-PCR and qPCR

Genomic DNA extraction was performed as described in Supplemental Information. Reverse Transcription Polymerase Chain Reactions (qRT-PCR) and Quantitative Polymerase Chain Reactions (qPCR) were performed on a 7500 Real Time PCR System (Applied Biosystems) using the SYBR Green-detection system (FS Universal SYBR Green MasterRox/Roche Applied Science). The complete list of oligonucleotides is reported in the Supplemental Table S1.

### FACS analysis

For the FACS analysis, HeLa-DR-GFP cells were harvested and re-suspended in 500 μl of PBS at density of 10^6^ cells/ml. Cell viability was assessed by Propidium Iodide (PI) staining: before FACS analysis cells were incubated with 3 μM PI for 10 min. Cytofluorimetric analysis was performed on a 9600 Cyan System (Dako Cytometrix), PI positive cells were excluded from the analysis by gating the PI-negative cells on a FSC-Linear vs FL2H-Log plot; GFP + cells were identified with a gate (R1) on a FL1H-Log vs SS-Log plot. Rec L and Rec H cells were identified on a FL1H Histogram of the R1-gated cells with 2 range-gate (see Fig. 4A). The same gate was used for all flow cytometry experiments.

Population comparison was performed using the FlowJo software (Chi-Squared Test). Differences in fluorescence intensity (mean) were determined using the matched pairs Student’s t test.

### Bisulfite DNA preparation, PCR, and sequence analysis

(The detailed protocol is described in the Supplemental Methods). A total of 2 μg of genomic DNA was bisulfite_converted according to the EZ DNA Methylation Kit (Zymo Research). Methylation status was assessed through a strategy based on the locus- specific amplification of bisulfite-treated genomic DNA, amplifying each amplicon separately, followed by Illumina MiSeq sequencing. The sequence of the bisulfite-specific primers used for this analysis is reported Supplemental Table S1. The methods involved two PCR steps, following Illumina recommended procedure. The pool of amplicons was subjected to sequencing using MiSeq system (V3 reagents kits). Sequencing was performed by 281 cycles (paired-end sequencing). Sequences in FASTQ format by Illumina sequencing machine were initially processed with Paired-End reAd mergeR (PEAR) data for an initial quality filtering and assembling (R1 plus R2). Only those sequences with a threshold quality score of ≥30 and an overlapping region within paired-end reads of 40 nt were processed with PReprocessing and INformation of SEQuence (Prinseq) to obtain FASTA for further analysis. Reads were aligned to the bisulfite converted reference sequence. Reads with ambiguous calls at the CpG dinucleotide were removed. After filtering, an average of 25,607.875 (range: 2,389–81,923) amplicon reads were obtained from each sample. Methylation states were estimated by counting the number of base calls (T/C) at CpG sites in the mapped reads compared to the referent on both strands (% methylation).

### Chromatin Immune-Precipitation (ChIP)

Cells were transfected and/or treated as indicated in the legends of the figures. The cells (~2.5 × 10^6^ for each antibody) were fixed for 10 min at room temperature by adding 1 volume of 2% formaldehyde to a final concentration of 1%; the reaction was quenched by addition of glycine to a final concentration of 125 mM. Fixed cells were harvested and the pellet was re-suspended in 1 ml of lysis Buffer containing 1× protease inhibitor cocktail (Roche Applied Science). The lysates were sonicated to have DNA fragments 300 to 600 bp. Sonicated samples were immunoprecipitated as described in Supplemental Information and the DNA was recovered and subjected to qPCR using the primers indicated in the legend of the specific figures, primers sequences are described in Supplemental Table S1.

### MeDIP

Cells were transfected and/or treated as indicated in figure legends. A total of ~5 × 10^6^ cells were harvested and Genomic DNA extracted as described above. Ten micrograms of total genomic DNA was digested in 200 μl for 16 h with Restriction Endonuclease mix containing 30 U each of Eco RI, Bam HI, Hind III, XbaI, Sal I (Roche Applied Science), phenol/chloroform extracted, ethanol precipitated and resuspended in 50 μl of TE buffer. An aliquot (1/10) of digested DNA was used as input control to determine DNA concentration and digestion efficiency. MeDIP was performed essentially as described^32^ except that 2 μg of antibody specific for 5 mC (Abcam) were used to precipitate methylated DNA from 5 μg of total genomic DNA.

### Chromosome conformation capture (3C)

The 3C assay was performed as described previously^18^,^33^ with minor modifications. Briefly, the Hae III restriction enzyme, which cleaves pDR-GFP and generates 55 fragments was used. Digestion was performed on formaldehyde-fixed nuclei with 150 U of restriction enzyme at 37 °C for 16 h. The restriction enzyme was inactivated by addition of SDS to 2% and incubation at 65 °C for 30 min. The reaction was diluted into 4 ml ligation buffer containing 50 U of T4 DNA Ligase and incubated at 16 °C for 18 h. Samples were de-crosslinked by incubation at 65 °C in the presence of proteinase K for 15 min, purified by phenol/chloroform extraction and ethanol precipitated. Samples were re-dissolved in 20 μl of TE buffer. Primer sequences are shown in Supplemental Table S1.

### Statistical analysis

All data (with exception of Fig. 4E) are presented as mean ± standard deviation in at least three experiments in triplicate (n ≥ 9). Data sets were analyzed statistically using *JMP Statistical Discovery™* software by SAS and tested for normality using the Shapiro-Wilks test (“normal distribution fit” tool -JMP software). Two-tailed significance tests were performed with p < 0.05 considered significant. Non-parametric analyses were done with the Mann-Whitney-U-Test (Wilcoxon rank-sum test), parametric with the t-test. Detailed statistical analysis of the data shown in Figs 1 and 2 is reported in Supplemental Statistical Tables 1, 2, 3 and 4.

## Additional Information

**How to cite this article**: Russo, G. *et al*. DNA damage and Repair Modify DNA methylation and Chromatin Domain of the Targeted Locus: Mechanism of allele methylation polymorphism. *Sci. Rep.* **6**, 33222; doi: 10.1038/srep33222 (2016).

## Supplementary Material

Supplementary Information
