# Supplementary material for: DNA damage and Repair Modify DNA methylation and Chromatin Domain of the Targeted Locus: Mechanism of allele methylation polymorphism
Source: Sci Rep. 2016 Sep 15;6:33222. doi: 10.1038/srep33222 (PMC5024116; doi:10.1038/srep33222)
Supplement: Supplementary Information [file srep33222-s1.pdf]

# Supplementary data for

# DNA damage and Repair Modify DNA methylation and Chromatin Domain of the Targeted Locus.

### Mechanism of allele methylation polymorphism

**Giusi Russo[1^], Rosaria Landi[1^], Antonio Pezone[1^], Annalisa Morano[1], Candida Zuchegna[2], Antonella Romano[2], Mark T. Muller[3], Max E. Gottesman[4], Antonio Porcellini[2*], Enrico V. Avvedimento[1*]**

1. *Dipartimento di Medicina Molecolare e Biotecnologie Mediche, Istituto di Endocrinologia ed Oncologia Sperimentale del C.N.R., Università Federico II, 80131 Napoli, Italy*
2. *Dipartimento di Biologia, Università Federico II, 80126 Napoli, Italy*
3. *Epigenetics Division, TopoGEN, Inc., 27960 CR319, Buena Vista, Colorado, USA 81211*
4. *Institute of Cancer Research, Columbia University Medical Center, New York, New York, USA 10032.*

**Supplement Index:**

| | |
|---|---|
| Supplementary Methods and References | pages 02-06 |
| Summary of Supplementary Tables and Figures | page 07 |
| Supplementary Table S1 | page 08 |
| Supplementary Table S2 | page 08 |
| Supplementary Table S3 | page 10 |
| Supplementary Figures legends | pages 11 |
| Supplementary Figures S1-S3 | pages 12-14 |
| Supplementary Statistical Tables | pages 15-39 |

## Supplemental Methods

Cell culture, transfections and plasmids. Transfection efficiency was measured by assaying β-galactosidase activity of an included pSVbGal vector (Promega). Normalization by FACS was performed using antibodies to β-gal or pCMV-DsRed-Express (Clontech). pEGFP (Clontech) was used as GFP control vector.

**Vectors.** pDR-GFP plasmid was 14,735 bp containing (see Fig. 2): CMV IE enhancer (1–385); chicken beta actin promoter (386-751); chicken beta-actin first intron (752-1,622); rabbit beta-globin second intron (1,623-1,670); rabbit beta-globin third exon (1,671-1,724); EGFP with a STOP (7) codon at I-SceI site (1,740-2,756, I-SceI at 2,135); SV40 splice/polyadenylation signal (2,757-3,023); polyadenylation signal from phosphoglycerate kinase gene (3,025-3,607) for the puromycin resistance gene (3,600-4,200); a truncated EGFP gene sequence (5,609-6,138); and 6,450 bp of mouse genome *(6 and A. Porcellini, unpublished data)*.

siRNA against TDG: cat.# SR304768; OriGene Technologies, Inc., USA

shRNA of APE1 and APE1WT plasmids: The following oligonucleotides were used for the short hairpin RNA (shRNA) of APE1: sense, 5'-GATCCCC CCTGCCACACTCAAGATCTGCTTCAAGAGAGCAGATCTTGAGTGTGGCA GGTTTTTGGAAA-3'; and antisense, 5'-AGCTTTTCCAAAAACCTGCCACA CTCAAGATCTGCTCTCTTGAAGCAGATCTTGAGTGTGGCAGGGGG-3'. These sequences were drawn following the empirical rules of Mittal *(Mittal, V. 2004)* and were designed to recognize and bind to a 21-base sequence (underlined) placed 175 nucleotides after the AUG initiation codon of the APE1 gene. As a control, we used the following scrambled oligonucleotide sequences: sense, 5'-GATCCCCAGTCTAACTCGCCACCCCGTATTCAAGA GATACGGGGTGGCGAGTTAGACTTTTTGGAAA-3'; antisense, 5'-AGCTT TTCCAAAAAAGTCTAACTCGCCACCCCGTATCTCTTGAATACGGGGTGG CGAGTTAGACTGGG-3'. These sequences were checked with BLAST (http://www.ncbi.nlm.nih.gov/blast/) for their inability to pair with any human cDNA sequence. The sequences were cloned into Bgl II and Hind III restriction sites of pSUPER vector (Oligoengine) to form the so-called pSUPER-APE1 vector.

The pFLAG-CMV-5.1/APE1-M2 expression vector was generated by modifing the pFLAG-CMV-5.1/APE1 *(Vascotto et al. 2009)*. To avoid the degradation of the ectopic APE1 mRNA by the specific siRNA sequence described above, two nucleotides of the APE1-cDNA coding sequence were mutated with site-directed mutagenesis kits (Stratagene), leaving the APE1 amino acid sequence unaffected: siRNA, 5'-CCTGCCACACTCAAGATCTGC-3'; and APE1, 5'-CCTGCAACGCTCAAGATCTGC-3'.

The expression vectors for OGG1WT ad for the K338R/K341R OGG1 mutant was the FLG-Tagged vectors previously described by Bhakat *(31)*.

**RNA and DNA extraction.** Total RNA was extracted using Triazol (Gibco/Invitrogen). Genomic DNA extraction was performed with following protocol: cellular pellet was resuspended in 10 mM TRIS (pH 7.8) and 50 mM NaCl solution ($2x10^7$ cells/ml). After addition of 1% SDS the sample was gently mixed. Proteinase K, at a final concentration of 200 µg/ml, was added

and the mixture was incubated at 55 °C overnight. The following day, hot NaCl solution (70 °C) was added to the mixture at final concentration of 1.5 M and the DNA was extracted by chloroform. DNA was ethanol precipitated, dried, and resuspended in TE buffer. Total RNA was extracted using TRIzol (Gibco/Invitrogen). cDNA was synthesized in a 20 μl reaction containing 1 μg of total RNA, 100 U of Superscript III Reverse Transcriptase (Invitrogen), and 2 μl random hexamer (20 ng/μl) (Invitrogen). mRNA was reverse-transcribed for 1 h at 50 °C, and the reaction was heat inactivated for 15 min at 70 °C. The products were stored at-20 °C.

**Antibodies used for the experiments.** Pol II 05-623 (Upstate); H3K4me2 ab32356 (Abcam); H3K4me3 ab1012 (Abcam); H3K9me2 ab1220 (Abcam); H3K9me3 ab8898 (Abcam); Total H3 ab1791 (Abcam); APE1 ab-194 (Abcam); Anti-5-methylcytosine (5-mC) antibody [EP4694] ab124936 (Abcam); GAPD ab9482 (Abcam); TDG sc-292440 (Santa Cruz Biotechnology); MCM7 sc-9966 (Santa Cruz Biotechnology); Normal rabbit IgG sc-2027 (Santa Cruz Biotechnology); Normal mouse IgG sc-2025 (Santa Cruz Biotechnology).

**Chromatin Immuno-Precipitation (ChIP).** Cells were transfected and/or treated as indicated in the legends of the figures. The cells (~2.5 x$10^6$ for each antibody) were fixed for 10 min at room temperature by adding 1 volume of 2% formaldehyde to a final concentration of 1%; the reaction was quenched by addition of glycine to a final concentration of 125 mM. Fixed cells were harvested and the pellet was resuspended in 1 ml of Lysis Buffer (See Below) containing 1X protease inhibitor cocktail (Roche Applied Science). The lysates were sonicated to have DNA fragments 300 to 600 bp. Sonicated samples were centrifuged and supernatants diluted 2 fold in the ChIP Buffer (See Below). An aliquot (1/10) of sheared chromatin was further treated with proteinase K, extracted with phenol/chloroform and precipitated to determine DNA concentration and shearing efficiency (input DNA). The ChIP reaction was set up according to the manufacturer's instructions. Briefly, the sheared chromatin was precleared for 2 h with 1 μg of non-immune IgG and 20 μl of Protein A/G PLUS-Agarose (Santa Cruz Biotechnology) saturated with salmon sperm (1 mg/ml). Precleared chromatin was divided in aliquots and incubated at 4 °C for 16 h with 1 μg of the specific antibody (see above) and non-immune IgG respectively. The immuno-complexes were recovered by incubation for 3 h at 4 °C with 20 μl of protein-A/G agarose, beads were washed with wash buffers according to the manufacturer's instructions and immunoprecipitated DNA was recovered and subjected to qPCR using the primers indicated in the legend of the specific figures, primers sequences are described in Supplemental Table S1.

**Chromosome conformation capture (3C).** The actual position of oligonucleotides and Hae III restriction sites are shown in Figs. 3A and 3BB.

A total of 5x$10^6$ cells were crosslinked in 20 ml of serum-free medium with 1% formaldehyde for 10 min at room temperature. The reaction was quenched by the addition of glycine to a final concentration of 125 mM. Fixed cells were harvested and the pellet resuspended in 0.5 ml of ice cold lysis buffer (10 mM Tris pH 8.0, 10 mM NaCl, 0.2% NP40 and protease inhibitors),

and incubated on ice for 15 min. Nuclei were washed with 0.25 ml of restriction enzyme buffer and pelleted. Nuclei were then resuspended in 180 µl of restriction enzyme buffer. SDS was added to a final concentration of 0.1%, and nuclei were incubated at 37 °C for 15 min. Triton X-100 was then added to the final concentration of 1% to sequester SDS. Digestion was performed with 150 U of restriction enzyme at 37 °C for 16 h. The restriction enzyme was inactivated by the addition of SDS to 2% and incubation at 65 °C for 30 min. The reaction was diluted into 4 ml ligation reaction buffer containing 50 U of T4 DNA Ligase (Roche Applied Science). Ligations were incubated at 16 °C for 18 h. EDTA (to a concentration of 10 mM) was added to stop the reactions. Samples were treated with Proteinase K (200 µg/ml) and incubated for 2 h at 50 °C, and then overnight at 65 °C to reverse the formaldehyde crosslinks. The following day, the DNA was purified by phenol/chloroform extraction and ethanol precipitation. Samples were redissolved in 20 µl of TE buffer. To prepare a control template, we used pDR-GFP plasmid. Five micrograms of plasmid DNA were digested with Hae III in 50 µl of 1x buffer for 8 h at 37 °C and then ligated in 20 µl with 5 U of T4 Ligase at 16 °C for 4 h. The efficiency of digestion after the entire 3C treatment was quantified by realtime PCR, amplifying a fragment spanning two Hae III sites (uncut) in different 3C DNA preparations. A total of 35 rounds of PCR amplification were used. The efficiency of ligation was assayed as follow: a linear Hind III-digested pUC18 plasmid was added to all the preparations before ligation. The ligated plasmid was quantified by real-time PCR, amplifying a fragment spanning the Hind III site (M13/pUC Forward and Reverse primers were used). An appropriate amount of DNA that would amplify within the linear range was subsequently used for the experiments. The efficiency of digestion was quantified by realtime PCR, amplifying three fragments spanning three different Hae III sites. The presence of the amplified products indicated the efficiency of digestion, and was expressed as ratio between cleaved/un-cleaved band. Experiments with a cutting Ratio >0.03 ($\Delta$CT≥4, 97% digestion) were discarded. Primers *m* and *e*; *p* and *Rec2* and *Sce/Bcg* and *q* were used.

**Bisulfite treatment and amplicon library preparation.** 2 µg of genomic DNA were converted with “C/T conversion reagent” according EZ DNA Methylation Kit (Zymo Research, USA) and eluted in 50 µl of $H_2O$ following the manufacturer’s instruction. We generated an amplicon library of bisulfite treated DNA using a double step PCR strategy. In the first PCR reaction, we amplified amplicons ranging in size between 300-450bp (all primers pairs are reported in Supplemental Table S1). The 5’ end of these primers contained overhang adapter sequences (Fw: 5’-TCGTCGGCAGCGTCAGATGTGTA TAAGAGACAG-3’, RV: 5’-GTCTCGTGGGCTCGGAGATGTGTATAAGAG ACAG-3’) that were used in the second step of PCR to add multiplexing indices and illumina sequencing adapters. First PCR products was performed using a “FastStart High Fidelity PCR System”(Roche) at the following thermo cycle condition: one cycle at 95°C for 2 min followed by 30 cycles at 95°C for 30 sec, at TM °C for 40 sec, at 72°C for 60 sec , followed by a final extension step at 72°C for 5 min. Reactions were performed in 30 µl total volumes: 3 µl 10x reaction buffer, 0.6 µl of 10 mM dNTP mix, 1 µl of 4 µM forward and reverse primers, 3.6 µl $MgCl_2$ 25 mM, 2-4 µl bisulfite template DNA, 0.25 µl FastStart Taq, and $H_2O$ up to a final volume of 30 µl. Five microliters of first

PCRs were used to check product size on 1.5% agarose gel. To eliminate small DNA fragments (primers dimers), we used 20 µl of AMPure purification magnetic Beads (Beckman-Coulter, Brea, CA, USA) following the manufacturer's protocol. Second PCR step was performed in 50 µl total volumes: 5 µl 10x reaction buffer, 1 µl dNTP mix, 5 µl forward and reverse "Nextera XT" primers (Illumina, SanDiego, CA, USA), 6 µl $MgCl_2$ 25 mM, 5 µl of first PCR product, 0.4 µl FastStart Taq, and $H_2O$ up to a final volume of 50 µl. Thermo-cycle settings were: one cycle at 95°C for 2 min followed by 8 cycles at 95°C for 30 sec, 55°C for 40 sec, 72°C for 40 sec, followed by a final extension step at 72°C for 5 min. Another purification step with 50.8 µl of AMPure Beads , was performed and all amplicons were quantified using Qubit® 2.0 Fluorometer. The quality of each amplicon was checked by Agilent 2100 Bioanalyzer using DNA 1000 Kit (Agilent Technologies, Santa Clara, CA, U.S.) according to the manufacturer's instructions. Amplicons were pooled at equimolar ratio and then diluted to final concentration of 8 picomolar. 15% (v/v) of Phix control libraries (Illumina, San Diego, CA, USA) was combined with normalized library to increase diversity of base calling during sequencing. Amplicons library was subjected to sequencing using V3 reagents kits on Illumina MiSeq system (Illumina, San Diego, CA, U.S.A). Pair-end sequencing was carry out in 281 cycles per read (281 x 2). An average of 25,607.875 reads/sample was used to further analysis.

**Sequence analysis.** First, FASTQC software (http://www.bioinformatics.bbsrc.ac.uk/projects/fastqc) was used to quality check FastQ files obtained from Illumina Miseq sequencer. Paired-end reads from sequencer platform were merged together using PEAR tool (*Zhang J.)* with a minimum of 40 overlapping residues as threshold and quality filtered was obtained using as threshold a mean PHREAD score of at least 33. Finally, FASTQ assembled reads were converted to FASTA format using PRINSEQ tool (Schmieder R). To analyze the methylation status of each amplicon, we used a homemade pipeline (Amplimethprofiler) specifically designed for deep targeted bisulfite amplicon sequencing of multiple genomic regions. This pipeline was made freely available at https://sourceforge.net/projects/amplimethprofiler. "Amplimethprofiler" recognizes corresponding target region discarding PCR artifacts and reads that do not match expected lengths. Next, reads were aligned to the corresponding bisulphite-converted reference using BLASTn (Camacho C). A filter of 99% bisulfite conversion efficiency was used. The pipeline output format reports the methylation status for each CpG dinucleotide coded 0 if the site is recognized as unmethylated, 1 if the site is recognized as methylated, and 2 if the methylation state could not be assessed. Quantitative methylation average for each site are then computed as sum of the number of non-converted bases mapped on that site over the total number of mapped reads (quantitative methylation analysis) for both strands.

**Buffers Formulation.**

3C Buffer 1: 10 mM Tris pH 8.0, 10 mM NaCl, 0.2% NP40 and protease inhibitors.
3C Ligation Buffer: 66 mM Tris-HCl, 5 mM $MgCl_2$, 5 mM DTT, 1 mM ATP, pH 7.5.
ChIP Lysis Buffer: 10 mM Tris-HCl pH 8.0, 10 mM NaCl, 0.2 % NP40.
ChIP Buffer: 1% Triton X-100, 2 mM EDTA, 150 mM NaCl, 20 mM Tris-HCl pH 8.0.

**Supplemental References:**

Mittal, V. (2004). Improving the efficiency of RNA interference in mammals. Nat. Rev. Genet. 5, 355–365.

Vascotto C, Fantini D, Romanello M, Cesaratto L, Deganuto M et al. (2009) APE1/Ref-1 interacts with NPM1 within nucleoli and plays a role in the rRNA quality control process. Mol Cell Biol 29, 1834-1854.

| |
|---|
| **Table S1. Complete list of DNA oligonucleotides used for PCR.** |
| **Table S2. Inhibition of BER during repair reduces transcription and increases methylation of the repaired DNA.** |
| **Table S3. Summary of the analyzed sequences.** |
| **Figure S1. Recruitment of RNA polymerase II large subunit (A) and the apurinic nuclease APE1 (B) along the GFP gene in H and L cells.** |
| **Figure S2. Silencing APE1, TDG or inactivating OGG1 reduces GFP expression.** |
| **Figure S3. Rescue of GFP expression in cells in which the activity of OGG1 and APE1 has been altered by expressing the dominant negative mutants.** |
| **Statistical Table 1. Statistical analysis of data presented in Figure 1, panel B** |
| **Statistical Table 2. Statistical analysis of data presented in Figure 1, panel C** |
| **Statistical Table 3. Statistical analysis of data presented in Figure 2, panel B** |
| **Statistical Table 4. Statistical analysis of data presented in Figure 2, panel C** |

## Table S1

| ID | PRIMERS | Locus |
|---|---|---|
| 3C Primer a | 5'-GGCGGGGCGAGGCGGAGA-3' | pDR-GFP |
| 3C Primer b | 5'-GAGTCGCTGCGTTGCCTTC-3' | pDR-GFP |
| 3C/ChIP Primer c | 5'-CGCCCGCAGCGCTCACAGC-3' | pDR-GFP |
| 3C/ChIP Primer d | 5'-ACGTGCTGGTTATTGTGCTGTC-3' | pDR-GFP |
| 3C/ChIP Primer e | 5'-TCCTGCTCCTGGGCTTCTCG-3' | pDR-GFP |
| 3C Primer f | 5'-AAGATGGTGCGCTCCTGGACGTA-3' | pDR-GFP |
| 3C Primer g | 5'-GGTGAAGTTCGAGGGCGACAC-3' | pDR-GFP |
| 3C/ChIP Primer h | 5'-TGCACGCTGCCGTCCTCG-3' | pDR-GFP |
| Primer Rec2 | 5'-CGGCGGCGGTCACGAACTC-3' | pDR-GFP |
| 3C Primer i | 5'-GCTGATCTCGTTCTTCAGGC-3' | pDR-GFP |
| 3C Primer l | 5'-GGTACTCTGTTCTCACCCTTC-3' | pDR-GFP |
| 3C Primer m | 5'-GAAAGCGAAGGAGCAAAGCTG-3' | pDR-GFP |
| ChIP Primer a | 5'-CGTTACTCCCACAGGTGAGC-3' | pDR-GFP |
| SS3/ChIP Primer r | 5'-AGTTCATCTGCACCACCG-3' | pDR-GFP |
| ChIP Primer p | 5'-GGCGTAAATTGTAAGCGTTA-3' | pDR-GFP |
| ChIP Primer q | 5'-GCCTGAAGAACGAGATCAGC-3' | pDR-GFP |
| SS2/ChIP Primer s | 5'-CAGGGTAATACCTACGGC-3' | pDR-GFP |
| SS1/ChIP Primer t | 5'-AACGGCCACAAGTTCAGC-3' | pDR-GFP |
| AS3/ChIP Primer u | 5'-ATGAACTTCAGGGTCAGC-3' | pDR-GFP |
| AS2/ChIP Primer v | 5'-ATCCCTAGCCGGACACGC-3' | pDR-GFP |
| AS1/ChIP Primer z | 5'-TTGTGGCCGTTTACGTCG-3' | pDR-GFP |
| Primer I-SceI | 5'-GCTAGGGATAACAGGGTAAT-3' | pDR-GFP |
| Primer Bcg | 5'-GAGGGCGAGGGCGATGCC-3' | pDR-GFP |
| Bisulfite E01F | 5'-GTGTGATTGGTGGTTTTAGAGT-3' | pDR-GFP |
| Bisulfite E02R | 5'-CCATCCTCAATATTATAACAAAT-3' | pDR-GFP |
| Bisulfite EN01F | 5'-GGAGTTGTTTATTGGGGTGGTGTTTATTTTGGT-3' | pDR-GFP |
| Bisulfite EN02R | 5'-GTTTGTGTTTTAGGATGTTGTTG-3' | pDR-GFP |
| Minus/Bisulfite E3F | 5'-GTATTTTAGTTTGTGTTTTAGGATG -3' | pDR-GFP |
| Minus/Bisulfite E4R | 5'-CACCTAAAACTAAAACACT-3' | pDR-GFP |
| Plus/Bisulfite E5F | 5'-AGGAGGTATTTGGAGTTGAGGTA-3' | pDR-GFP |
| Plus/Bisulfite E6R | 5'-TACTCCAACTTATACCCCAAAATAT -3' | pDR-GFP |
| MEDIP I F | 5'-TGTTTTGGTTGGCGTAAGGC-3' | Mouse Rosa26 |
| MEDIP I R | 5'-CGGCGGGTAAAACGACTCC-3' | Mouse Rosa26 |
| MEDIP II F | 5'-AGAAAACTGGCCCTTGCCAT-3' | Mouse Rosa26 |
| MEDIP II R | 5'-TTGAGGCAACTCAAGTCGGAA-3' | Mouse Rosa26 |
| 18S F | 5'-TCCCCATGAACGAGGAATTC-3' | h18S |
| 18S R | 5'-GTGTACAAAGGGCAGGGACTT-3' | h18S |

**Supplementary Table S1. Complete list of DNA oligonucleotides used for PCR**. On the left is shown the primer identification tag and the specific experiments (ID); on the center, the DNA sequence; on the right, the genes or loci corresponding to the specific primers.

# Tab. S2

**Matched Pairs Days= 5 d**

**Difference: OGG1 DN−OGG1 DN+wt**

| | | | |
|---|---|---|---|
| OGG1 DN | 122.889 | t−Ratio | −10.2524 |
| OGG1 DN+wt | 227.333 | DF | 8 |
| Mean Difference | −104.44 | Prob > \|t\| | <.0001* |
| Std Error | 10.1873 | Prob > t | 1.0000 |
| Upper95% | −80.953 | Prob < t | <.0001* |
| Lower95% | −127.94 | | |
| N | 9 | | |
| Correlation | −0.4037 | | |

**Difference: CTRL−OGG1 DN+wt**

| | | | |
|---|---|---|---|
| CTRL | 231.111 | t−Ratio | 0.40279 |
| OGG1 DN+wt | 227.333 | DF | 8 |
| Mean Difference | 3.77778 | Prob > \|t\| | 0.6977 |
| Std Error | 9.37902 | Prob > t | 0.3488 |
| Upper95% | 25.4058 | Prob < t | 0.6512 |
| Lower95% | −17.85 | | |
| N | 9 | | |
| Correlation | −0.1658 | | |

**Difference: CTRL−OGG1 DN**

| | | | |
|---|---|---|---|
| CTRL | 231.111 | t−Ratio | 10.52973 |
| OGG1 DN | 122.889 | DF | 8 |
| Mean Difference | 108.222 | Prob > \|t\| | <.0001* |
| Std Error | 10.2778 | Prob > t | <.0001* |
| Upper95% | 131.923 | Prob < t | 1.0000 |
| Lower95% | 84.5216 | | |
| N | 9 | | |
| Correlation | −0.0179 | | |

**Matched Pairs Days= 7 d**

**Difference: OGG1 DN−OGG1 DN+wt**

| | | | |
|---|---|---|---|
| OGG1 DN | 156.8 | t−Ratio | −9.92706 |
| OGG1 DN+wt | 266.2 | DF | 9 |
| Mean Difference | −109.4 | Prob > \|t\| | <.0001* |
| Std Error | 11.0204 | Prob > t | 1.0000 |
| Upper95% | −84.47 | Prob < t | <.0001* |
| Lower95% | −134.33 | | |
| N | 10 | | |
| Correlation | 0.34277 | | |

**Difference: CTRL−OGG1 DN+wt**

| | | | |
|---|---|---|---|
| CTRL | 256.4 | t−Ratio | −0.93338 |
| OGG1 DN+wt | 266.2 | DF | 9 |
| Mean Difference | −9.8 | Prob > \|t\| | 0.3750 |
| Std Error | 10.4995 | Prob > t | 0.8125 |
| Upper95% | 13.9516 | Prob < t | 0.1875 |
| Lower95% | −33.552 | | |
| N | 10 | | |
| Correlation | 0.51943 | | |

**Difference: CTRL−OGG1 DN**

| | | | |
|---|---|---|---|
| CTRL | 256.4 | t−Ratio | 15.29114 |
| OGG1 DN | 156.8 | DF | 9 |
| Mean Difference | 99.6 | Prob > \|t\| | <.0001* |
| Std Error | 6.51358 | Prob > t | <.0001* |
| Upper95% | 114.335 | Prob < t | 1.0000 |
| Lower95% | 84.8653 | | |
| N | 10 | | |
| Correlation | 0.74403 | | |

**Difference: OGG1 DN+wt−OGG1 DN+AZA**

| | | | |
|---|---|---|---|
| OGG1 DN+wt | 266.2 | t−Ratio | 0.597845 |
| OGG1 DN+AZA | 259 | DF | 9 |
| Mean Difference | 7.2 | Prob > \|t\| | 0.5647 |
| Std Error | 12.0433 | Prob > t | 0.2823 |
| Upper95% | 34.4437 | Prob < t | 0.7177 |
| Lower95% | −20.044 | | |
| N | 10 | | |
| Correlation | 0.27873 | | |

**Difference: OGG1 DN−OGG1 DN+AZA**

| | | | |
|---|---|---|---|
| OGG1 DN | 156.8 | t−Ratio | −11.6538 |
| OGG1 DN+AZA | 259 | DF | 9 |
| Mean Difference | −102.2 | Prob > \|t\| | <.0001* |
| Std Error | 8.76964 | Prob > t | 1.0000 |
| Upper95% | −82.362 | Prob < t | <.0001* |
| Lower95% | −122.04 | | |
| N | 10 | | |
| Correlation | 0.20697 | | |

**Difference: CTRL−OGG1 DN+AZA**

| | | | |
|---|---|---|---|
| CTRL | 256.4 | t−Ratio | −0.29417 |
| OGG1 DN+AZA | 259 | DF | 9 |
| Mean Difference | −2.6 | Prob > \|t\| | 0.7753 |
| Std Error | 8.8383 | Prob > t | 0.6124 |
| Upper95% | 17.3936 | Prob < t | 0.3876 |
| Lower95% | −22.594 | | |
| N | 10 | | |
| Correlation | 0.49977 | | |

**Matched Pairs Days=14 d**

**Difference: OGG1 DN−OGG1 DN+wt**

| | | | |
|---|---|---|---|
| OGG1 DN | 103.667 | t−Ratio | −15.3342 |
| OGG1 DN+wt | 273 | DF | 8 |
| Mean Difference | −169.33 | Prob > \|t\| | <.0001* |
| Std Error | 11.0428 | Prob > t | 1.0000 |
| Upper95% | −143.87 | Prob < t | <.0001* |
| Lower95% | −194.8 | | |
| N | 9 | | |
| Correlation | 0.20163 | | |

**Difference: CTRL−OGG1 DN+wt**

| | | | |
|---|---|---|---|
| CTRL | 260.222 | t−Ratio | −0.79638 |
| OGG1 DN+wt | 273 | DF | 8 |
| Mean Difference | −12.778 | Prob > \|t\| | 0.4488 |
| Std Error | 16.0449 | Prob > t | 0.7756 |
| Upper95% | 24.2218 | Prob < t | 0.2244 |
| Lower95% | −49.777 | | |
| N | 9 | | |
| Correlation | −0.6602 | | |

**Difference: CTRL−OGG1 DN**

| | | | |
|---|---|---|---|
| CTRL | 260.222 | t−Ratio | 19.6073 |
| OGG1 DN | 103.667 | DF | 8 |
| Mean Difference | 156.556 | Prob > \|t\| | <.0001* |
| Std Error | 7.98455 | Prob > t | <.0001* |
| Upper95% | 174.968 | Prob < t | 1.0000 |
| Lower95% | 138.143 | | |
| N | 9 | | |
| Correlation | 0.18311 | | |

**Difference: OGG1 DN+wt−OGG1 DN+AZA**

| | | | |
|---|---|---|---|
| OGG1 DN+wt | 273 | t−Ratio | 0.158416 |
| OGG1 DN+AZA | 270.333 | DF | 8 |
| Mean Difference | 2.66667 | Prob > \|t\| | 0.8781 |
| Std Error | 16.8333 | Prob > t | 0.4390 |
| Upper95% | 41.4844 | Prob < t | 0.5610 |
| Lower95% | −36.151 | | |
| N | 9 | | |
| Correlation | −0.1769 | | |

**Difference: OGG1 DN−OGG1 DN+AZA**

| | | | |
|---|---|---|---|
| OGG1 DN | 103.667 | t−Ratio | −13.8171 |
| OGG1 DN+AZA | 270.333 | DF | 8 |
| Mean Difference | −166.67 | Prob > \|t\| | <.0001* |
| Std Error | 12.0623 | Prob > t | 1.0000 |
| Upper95% | −138.85 | Prob < t | <.0001* |
| Lower95% | −194.48 | | |
| N | 9 | | |
| Correlation | 0.09455 | | |

**Difference: CTRL−OGG1 DN+AZA**

| | | | |
|---|---|---|---|
| CTRL | 260.222 | t−Ratio | −1.20104 |
| OGG1 DN+AZA | 270.333 | DF | 8 |
| Mean Difference | −10.111 | Prob > \|t\| | 0.2641 |
| Std Error | 8.41864 | Prob > t | 0.8680 |
| Upper95% | 9.3023 | Prob < t | 0.1320 |
| Lower95% | −29.525 | | |
| N | 9 | | |
| Correlation | 0.66404 | | |

**Matched Pairs Days= 5 d**

**Difference: shAPE1-shAPE1+wt**

| | | | |
|---|---|---|---|
| APE1 DN | 104.111 | t−Ratio | −15.5904 |
| APE1 DN+wt | 229.778 | DF | 8 |
| Mean Difference | −125.67 | Prob > \|t\| | <.0001* |
| Std Error | 8.06053 | Prob > t | 1.0000 |
| Upper95% | −107.08 | Prob < t | <.0001* |
| Lower95% | −144.25 | | |
| N | 9 | | |
| Correlation | 0.23618 | | |

**Difference: ctrl-shAPE1+wt**

| | | | |
|---|---|---|---|
| CTRL | 231.111 | t−Ratio | 0.110919 |
| APE1 DN+wt | 229.778 | DF | 8 |
| Mean Difference | 1.33333 | Prob > \|t\| | 0.9144 |
| Std Error | 12.0208 | Prob > t | 0.4572 |
| Upper95% | 29.0534 | Prob < t | 0.5428 |
| Lower95% | −26.387 | | |
| N | 9 | | |
| Correlation | −0.2593 | | |

**Difference: ctrl-shAPE1**

| | | | |
|---|---|---|---|
| CTRL | 231.111 | t−Ratio | 11.56805 |
| APE1 DN | 104.111 | DF | 8 |
| Mean Difference | 127 | Prob > \|t\| | <.0001* |
| Std Error | 10.9785 | Prob > t | <.0001* |
| Upper95% | 152.316 | Prob < t | 1.0000 |
| Lower95% | 101.684 | | |
| N | 9 | | |
| Correlation | −0.8002 | | |

**Matched Pairs Days= 7 d**

**Difference: shAPE1-shAPE1+wt**

| | | | |
|---|---|---|---|
| APE1 DN | 75.4 | t−Ratio | −19.0401 |
| APE1 DN+wt | 264.4 | DF | 9 |
| Mean Difference | −189 | Prob > \|t\| | <.0001* |
| Std Error | 9.9264 | Prob > t | 1.0000 |
| Upper95% | −166.54 | Prob < t | <.0001* |
| Lower95% | −211.46 | | |
| N | 10 | | |
| Correlation | 0.56666 | | |

**Difference: ctrl-shAPE1+wt**

| | | | |
|---|---|---|---|
| CTRL | 256.4 | t−Ratio | −0.80521 |
| APE1 DN+wt | 264.4 | DF | 9 |
| Mean Difference | −8 | Prob > \|t\| | 0.4415 |
| Std Error | 9.93535 | Prob > t | 0.7793 |
| Upper95% | 14.4753 | Prob < t | 0.2207 |
| Lower95% | −30.475 | | |
| N | 10 | | |
| Correlation | 0.58801 | | |

**Difference: ctrl-shAPE1**

| | | | |
|---|---|---|---|
| CTRL | 256.4 | t−Ratio | 16.12618 |
| APE1 DN | 75.4 | DF | 9 |
| Mean Difference | 181 | Prob > \|t\| | <.0001* |
| Std Error | 11.224 | Prob > t | <.0001* |
| Upper95% | 206.39 | Prob < t | 1.0000 |
| Lower95% | 155.61 | | |
| N | 10 | | |
| Correlation | −0.1029 | | |

**Difference: shAPE1-shAPE1+wt -AZA**

| | | | |
|---|---|---|---|
| APE1 DN+wt | 264.4 | t−Ratio | −0.16374 |
| APE1 DN+AZA | 265 | DF | 9 |
| Mean Difference | −0.6 | Prob > \|t\| | 0.8736 |
| Std Error | 3.66424 | Prob > t | 0.5632 |
| Upper95% | 7.68909 | Prob < t | 0.4368 |
| Lower95% | −8.8891 | | |
| N | 10 | | |
| Correlation | 0.95124 | | |

**Difference: ctrl-shAPE1+wt A**

| | | | |
|---|---|---|---|
| APE1 DN | 75.4 | t−Ratio | −18.0409 |
| APE1 DN+AZA | 265 | DF | 9 |
| Mean Difference | −189.6 | Prob > \|t\| | <.0001* |
| Std Error | 10.5095 | Prob > t | 1.0000 |
| Upper95% | −165.83 | Prob < t | <.0001* |
| Lower95% | −213.37 | | |
| N | 10 | | |
| Correlation | 0.40383 | | |

**Difference: ctrl-shAPE1**

| | | | |
|---|---|---|---|
| CTRL | 256.4 | t−Ratio | −0.86094 |
| APE1 DN+AZA | 265 | DF | 9 |
| Mean Difference | −8.6 | Prob > \|t\| | 0.4116 |
| Std Error | 9.98911 | Prob > t | 0.7942 |
| Upper95% | 13.9969 | Prob < t | 0.2058 |
| Lower95% | −31.197 | | |
| N | 10 | | |
| Correlation | 0.5621 | | |

**Matched Pairs Days=14 d**

**Difference: shAPE1-shAPE1+wt**

| | | | |
|---|---|---|---|
| APE1 DN | 65.1111 | t−Ratio | −13.3976 |
| APE1 DN+wt | 273.222 | DF | 8 |
| Mean Difference | −208.11 | Prob > \|t\| | <.0001* |
| Std Error | 15.5335 | Prob > t | 1.0000 |
| Upper95% | −172.29 | Prob < t | <.0001* |
| Lower95% | −243.93 | | |
| N | 9 | | |
| Correlation | −0.6172 | | |

**Difference: ctrl-shAPE1+wt**

| | | | |
|---|---|---|---|
| CTRL | 260.222 | t−Ratio | −0.7595 |
| APE1 DN+wt | 273.222 | DF | 8 |
| Mean Difference | −13 | Prob > \|t\| | 0.4693 |
| Std Error | 17.1164 | Prob > t | 0.7653 |
| Upper95% | 26.4706 | Prob < t | 0.2347 |
| Lower95% | −52.471 | | |
| N | 9 | | |
| Correlation | −0.5867 | | |

**Difference: ctrl-shAPE1**

| | | | |
|---|---|---|---|
| CTRL | 260.222 | t−Ratio | 22.21317 |
| APE1 DN | 65.1111 | DF | 8 |
| Mean Difference | 195.111 | Prob > \|t\| | <.0001* |
| Std Error | 8.78358 | Prob > t | <.0001* |
| Upper95% | 215.366 | Prob < t | 1.0000 |
| Lower95% | 174.856 | | |
| N | 9 | | |
| Correlation | −0.1412 | | |

**Difference: shAPE1-shAPE1+wt -AZA**

| | | | |
|---|---|---|---|
| APE1 DN+wt | 273.222 | t−Ratio | 0.088475 |
| APE1 DN+AZA | 272.889 | DF | 8 |
| Mean Difference | 0.33333 | Prob > \|t\| | 0.9317 |
| Std Error | 3.76755 | Prob > t | 0.4658 |
| Upper95% | 9.02132 | Prob < t | 0.5342 |
| Lower95% | −8.3547 | | |
| N | 9 | | |
| Correlation | 0.95503 | | |

**Difference: ctrl-shAPE1+wt A**

| | | | |
|---|---|---|---|
| APE1 DN | 65.1111 | t−Ratio | −13.4153 |
| APE1 DN+AZA | 272.889 | DF | 8 |
| Mean Difference | −207.78 | Prob > \|t\| | <.0001* |
| Std Error | 15.4881 | Prob > t | 1.0000 |
| Upper95% | −172.06 | Prob < t | <.0001* |
| Lower95% | −243.49 | | |
| N | 9 | | |
| Correlation | −0.4774 | | |

**Difference: ctrl-shAPE1**

| | | | |
|---|---|---|---|
| CTRL | 260.222 | t−Ratio | −0.69451 |
| APE1 DN+AZA | 272.889 | DF | 8 |
| Mean Difference | −12.667 | Prob > \|t\| | 0.5070 |
| Std Error | 18.2384 | Prob > t | 0.7465 |
| Upper95% | 29.3911 | Prob < t | 0.2535 |
| Lower95% | −54.724 | | |
| N | 9 | | |
| Correlation | −0.7219 | | |

**Supplementary Table S2. Inhibition of BER during repair reduces transcription and increases methylation of the repaired DNA.** Cytofluorimetric analysis of GFP expression in cells transfected with I-SceI and OGG1 dominant negative variant or shRNA targeting APE1 (figure S4B and S4C). 5azadC treatment was performed as indicated in Materials and Methods. Rec H and Rec L are indicated in Fig. S4.

| | | # of reads | | | |
|---|---|---|---|---|---|
| **Samples** | **Strand** | **Total** | **BcgI** | **I-SceI** | **% BcgI** |
| **CTRL** | *PLUS* | 54,210 | 28,174 | 26,036 | 52.0 |
| **I-SceI** | *PLUS* | 13,642 | 8,567 | 5,075 | 62.8 |
| **I-SceI +shAPE1** | *PLUS* | 7,268 | 5,606 | 1,662 | 77.1 |
| **I-SceI +shAPE1+wt** | *PLUS* | 2,389 | 1,812 | 577 | 75.8 |
| **CTRL** | *MINUS* | 81,923 | 39,508 | 42,415 | 48.2 |
| **I-SceI** | *MINUS* | 29,002 | 16,945 | 12,057 | 58.4 |
| **I-SceI +shAPE1** | *MINUS* | 10,929 | 7,722 | 3,207 | 70.7 |
| **I-SceI +shAPE1+wt** | *MINUS* | 5,500 | 3,657 | 1,843 | 66.5 |

**# of reads (Both Strands)**

| **Samples** | **Total** | **BcgI** | **I-SceI** | **% BcgI** | **Recombination index** |
|---|---|---|---|---|---|
| **CTRL** | 136,133 | 67,682 | 68,451 | 49.7 | 0 % |
| **I-SceI** | 42,644 | 25,512 | 17,132 | 59.8 | 17 % |
| **I-SceI +shAPE1** | 18,197 | 13,328 | 4,869 | 73.2 | 32 % |
| **I-SceI +shAPE1+wt** | 7,889 | 5,469 | 2,420 | 69.3 | 28 % |

**Supplementary Table S3. Summary of the analyzed sequences.** Bisulfite-DNA derived from the samples shown on the left column were subjected to deep sequence analysis of the DSB region (Table S1) 7 days after the transfection with the various constructs indicated. BcgI and I-SceI represent the number of sequences corresponding to the recombinant (BcgI) and unrecombinant (I-SceI) cassette. Hela cells contain a single copy of DRGFP integrated randomly in the genome and 1 copy of the I-SceI and BcgI cassettes (50% in the Control). The DSB (I-SceI), repaired by HR generates new BcgI cassettes. The increase of the fraction of sequences containing the BcgI represents an accurate measure of the recombination index. Methylation analysis expressed as percent methylated CpG when compared to control cells has been normalized to the recombination index.

**Supplementary Figure 1. Recruitment of RNA polymerase II large subunit and APE1 at the GFP gene in Rec H and Rec L cells.**

ChIP analysis with specific antibodies to the large fragment of RNA polymerase II (A) or APE1 (B) was carried out as described in Methods in control or H and L clones. GFP regions indicated as Prom, ATG, GFP3' and Poly-A correspond to the primers shown in Fig. 1A. The brackets and the * represent the comparison between different groups. *p<0.01 (*Wilcoxon rank-sum test*).

**Supplementary Figure 2. Silencing APE1, TDG or inactivating OGG1 reduces GFP expression and changes the Rec H/Rec L ratio. A and B.**

Cytofluorimetric analysis of cells exposed to OGG1 dominant negative expression vector, shRNA targeting APE1 or siRNA targeting TDG with or without a wild type TDG expressing vector, 2 days after transfection with I-SceI vector. The analysis was performed at days 5, 7, 14 after transfection with the constructs indicated. Aliquots taken at day 14 had been exposed 2 days prior ($12^{th}$ day following silencing) to 5-azadC as described in Methods. The fraction of RecH and RecL cells is shown by horizontal lines in each panel. The histograms on the right show the fraction of Rec H and Rec L clones in cells expressing the dominant-negative OGG1 or shAPE1. Statistical significant differences are indicated by horizontal lines using Student's matched pairs *t* test: *p<0.01 (*Wilcoxon rank-sum test*).

**C.** Immunoblot with antibodies recognizing human TDG or APE1 in total cell extracts from samples (indicated at the top of the panel) 48 h or 14 days after transfection with I-SceI and the various constructs indicated.

**Supplementary Figure 3. OGG1 and APE1 expression vectors restore GFP expression reduced by OGG1 or APE1 dominant negative variants.**

**A.** Cytofluorimetric analysis of cells exposed to human OGG1 or APE1 dominant-negative mutants with or without vectors expressing the wild type proteins, 2 days after exposure to I-SceI. The analysis was performed at days 5, 7, 14 after OGG1 or APE1 transfection. Aliquots taken at day 12 after silencing were exposed to 5-azadC as described in Methods. The fraction of RecH and RecL cells is shown by horizontal lines in each panel

**B and C.** Quantitative analysis of the intensity of fluorescence derived from A. The brackets and the * represent the comparison between different groups. *p<0.01 (*Wilcoxon rank-sum test*).

# Fig. S1

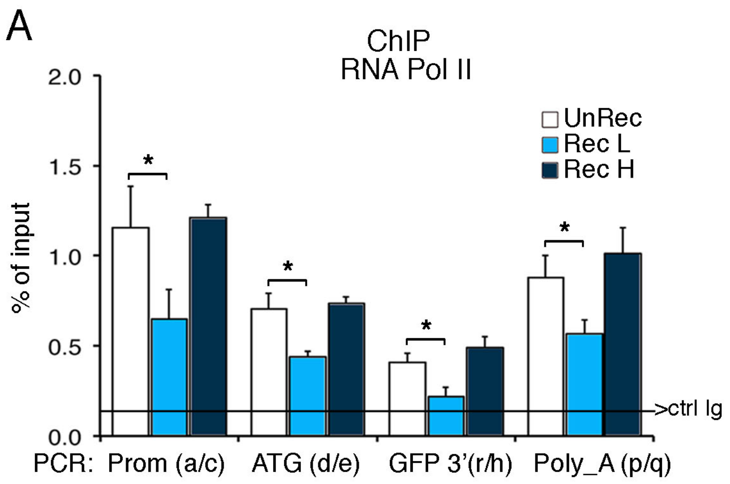
A
ChIP
RNA Pol II
UnRec
Rec L
Rec H
% of input
2.0
1.5
1.0
0.5
0.0
*
*
*
*
>ctrl Ig
PCR: Prom (a/c)
ATG (d/e)
GFP 3'(r/h)
Poly_A (p/q)

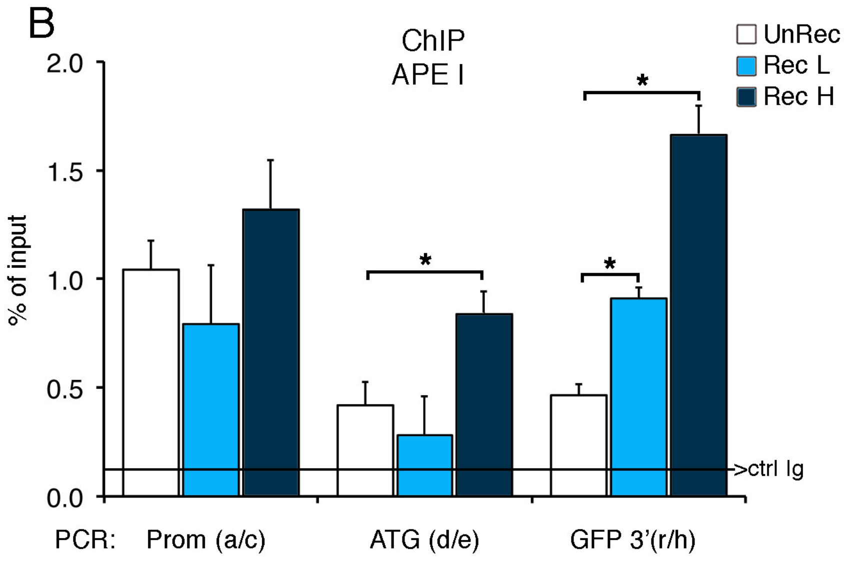
B
ChIP
APE I
UnRec
Rec L
Rec H
% of input
2.0
1.5
1.0
0.5
0.0
*
*
*
>ctrl Ig
PCR: Prom (a/c)
ATG (d/e)
GFP 3'(r/h)

# Fig. S2

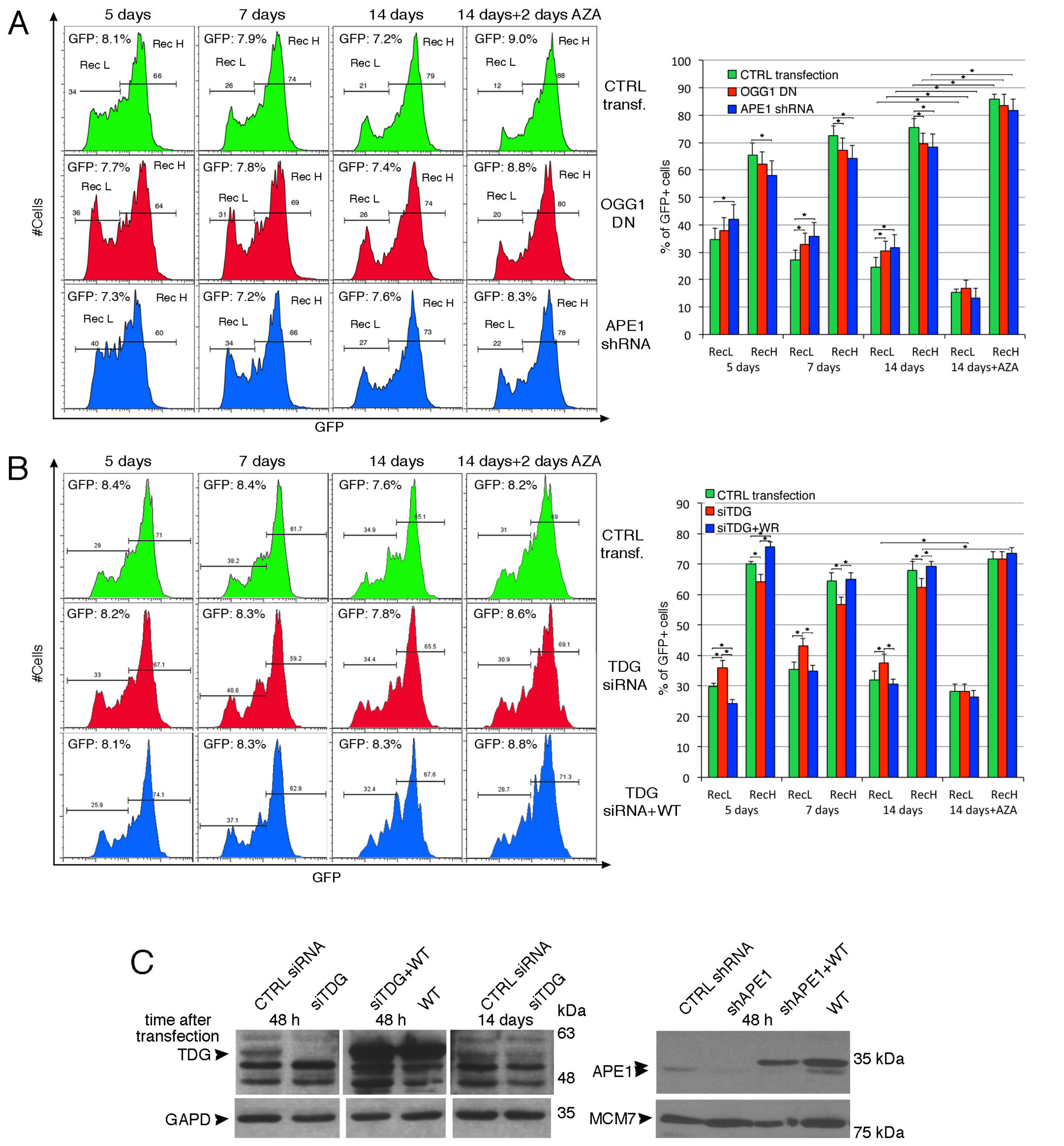
A
5 days
7 days
14 days
14 days+2 days AZA
CTRL transf.
OGG1 DN
APE1 shRNA
#Cells
GFP
CTRL transfection
OGG1 DN
APE1 shRNA
% of GFP+ cells
RecL
RecH
5 days
7 days
14 days
14 days+AZA
B
CTRL transf.
TDG siRNA
TDG siRNA+WT
CTRL transfection
siTDG
siTDG+WR
C
time after transfection
CTRL siRNA
siTDG
siTDG+WT
WT
48 h
14 days
kDa
TDG
GAPD
CTRL shRNA
shAPE1
shAPE1+WT
APE1
MCM7
35 kDa
75 kDa

## Fig. S3

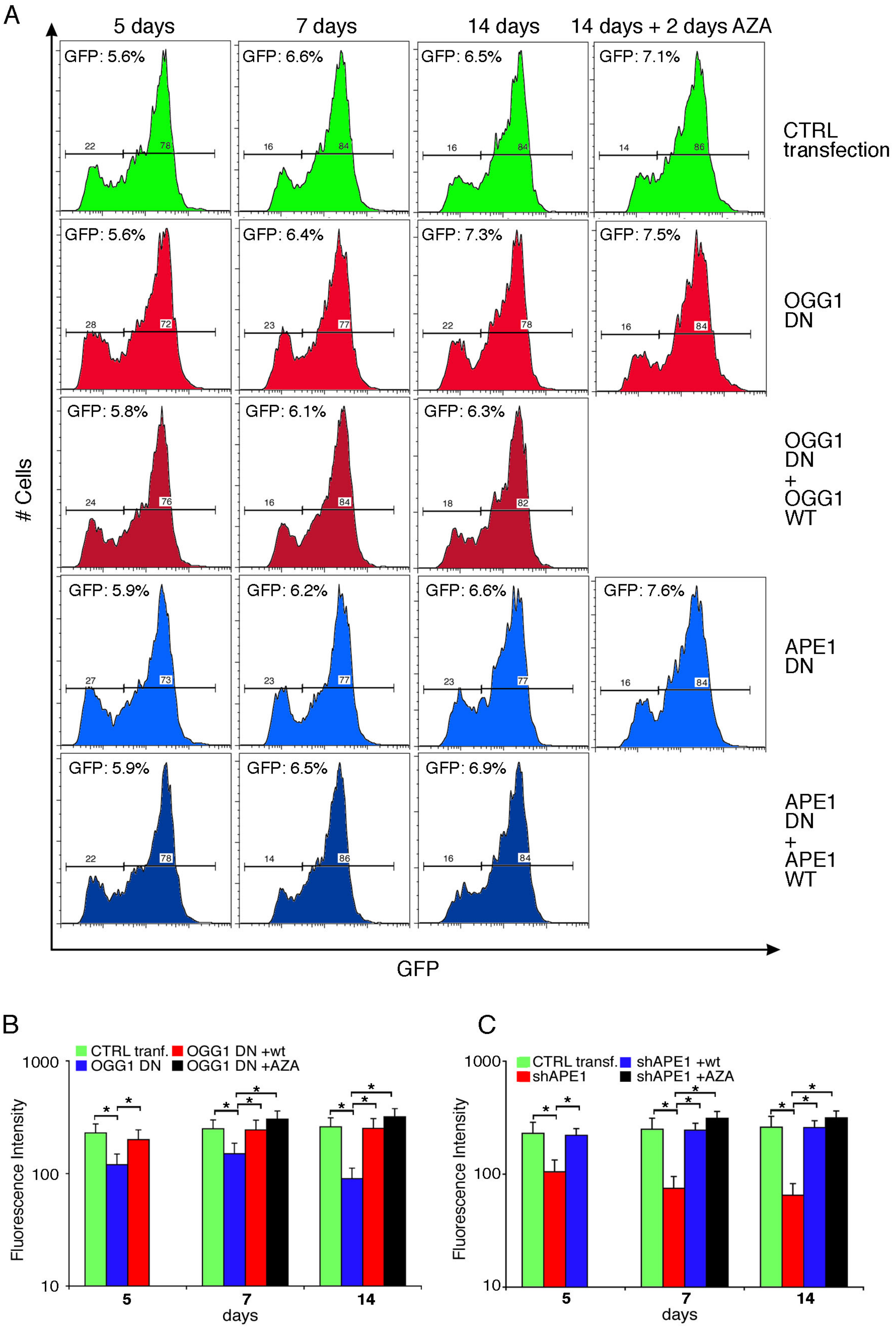
A
5 days
7 days
14 days
14 days + 2 days AZA
# Cells
GFP
CTRL transfection
OGG1 DN
OGG1 DN + OGG1 WT
APE1 DN
APE1 DN + APE1 WT
GFP: 5.6%
GFP: 6.6%
GFP: 6.5%
GFP: 7.1%
GFP: 5.6%
GFP: 6.4%
GFP: 7.3%
GFP: 7.5%
GFP: 5.8%
GFP: 6.1%
GFP: 6.3%
GFP: 5.9%
GFP: 6.2%
GFP: 6.6%
GFP: 7.6%
GFP: 5.9%
GFP: 6.5%
GFP: 6.9%
B
CTRL tranf.
OGG1 DN
OGG1 DN +wt
OGG1 DN +AZA
Fluorescence Intensity
1000
100
10
5
7
14
days
C
CTRL transf.
shAPE1
shAPE1 +wt
shAPE1 +AZA
Fluorescence Intensity
1000
100
10
5
7
14
days

Statistical Table 1.
Statistical analysis of data presented in Figure 1, panel B

## Fit Y by X Group

### Oneway Analysis of Ig-CTRL By TIME PCR=d/e

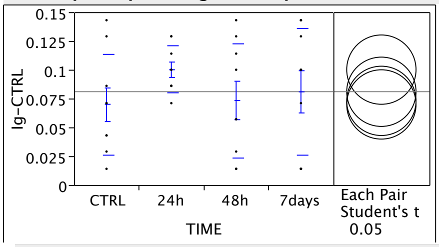


#### Means and Std Deviations

| Level | Number | Mean | Std Dev | Std Err Mean | Lower 95% | Upper 95% |
|---|---|---|---|---|---|---|
| CTRL | 9 | 0.069889 | 0.043793 | 0.01460 | 0.03623 | 0.10355 |
| 24h | 9 | 0.100111 | 0.020331 | 0.00678 | 0.08448 | 0.11574 |
| 48h | 9 | 0.073000 | 0.049820 | 0.01661 | 0.03471 | 0.11129 |
| 7days | 9 | 0.080889 | 0.055092 | 0.01836 | 0.03854 | 0.12324 |

#### Wilcoxon / Kruskal-Wallis Tests (Rank Sums)

| Level | Count | Score Sum | Score Mean | (Mean-Mean0)/Std0 |
|---|---|---|---|---|
| CTRL | 9 | 142.000 | 15.7778 | -0.883 |
| 24h | 9 | 202.500 | 22.5000 | 1.306 |
| 48h | 9 | 151.500 | 16.8333 | -0.533 |
| 7days | 9 | 170.000 | 18.8889 | 0.110 |

##### 1-way Test, ChiSquare Approximation

| ChiSquare | DF | Prob>ChiSq |
|---|---|---|
| 2.1660 | 3 | 0.5387 |

#### Means Comparisons

##### Comparisons for each pair using Student's t

| Level | - Level | Difference | Lower CL | Upper CL | p-Value |
|---|---|---|---|---|---|
| 24h | CTRL | 0.0302222 | -0.012311 | 0.0727555 | 0.1575 |
| 24h | 48h | 0.0271111 | -0.015422 | 0.0696444 | 0.2034 |
| 24h | 7days | 0.0192222 | -0.023311 | 0.0617555 | 0.3642 |
| 7days | CTRL | 0.0110000 | -0.031533 | 0.0535333 | 0.6020 |
| 7days | 48h | 0.0078889 | -0.034644 | 0.0504222 | 0.7081 |
| 48h | CTRL | 0.0031111 | -0.039422 | 0.0456444 | 0.8825 |

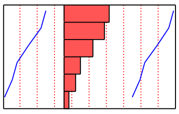

#### Power Details

Test TIME

##### Power

| α | σ | δ | Number | Power |
|---|---|---|---|---|
| 0.0500 | 0.044295 | 0.011755 | 36 | 0.2124 |

### Oneway Analysis of H3 By TIME PCR=d/e

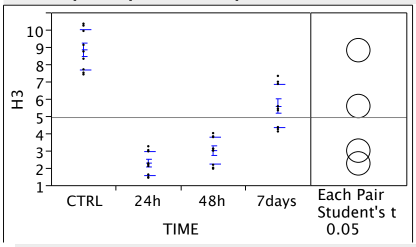


#### Means and Std Deviations

| Level | Number | Mean | Std Dev | Std Err Mean | Lower 95% | Upper 95% |
|---|---|---|---|---|---|---|
| CTRL | 9 | 8.81422 | 1.16470 | 0.38823 | 7.9190 | 9.7095 |
| 24h | 9 | 2.26211 | 0.69051 | 0.23017 | 1.7313 | 2.7929 |
| 48h | 9 | 3.00067 | 0.79748 | 0.26583 | 2.3877 | 3.6137 |
| 7days | 9 | 5.58200 | 1.23262 | 0.41087 | 4.6345 | 6.5295 |

#### Wilcoxon / Kruskal-Wallis Tests (Rank Sums)

| Level | Count | Score Sum | Score Mean | (Mean-Mean0)/Std0 |
|---|---|---|---|---|
| CTRL | 9 | 288.000 | 32.0000 | 4.421 |
| 24h | 9 | 66.000 | 7.3333 | -3.653 |
| 48h | 9 | 105.000 | 11.6667 | -2.229 |
| 7days | 9 | 207.000 | 23.0000 | 1.461 |

##### 1-way Test, ChiSquare Approximation

| ChiSquare | DF | Prob>ChiSq |
|---|---|---|
| 30.3153 | 3 | <.0001* |

#### Means Comparisons

##### Comparisons for each pair using Student's t

| Level | - Level | Difference | Lower CL | Upper CL | p-Value |
|---|---|---|---|---|---|
| CTRL | 24h | 6.552111 | 5.59325 | 7.510970 | <.0001* |
| CTRL | 48h | 5.813556 | 4.85470 | 6.772415 | <.0001* |
| 7days | 24h | 3.319889 | 2.36103 | 4.278748 | <.0001* |
| CTRL | 7days | 3.232222 | 2.27336 | 4.191082 | <.0001* |
| 7days | 48h | 2.581333 | 1.62247 | 3.540193 | <.0001* |
| 48h | 24h | 0.738556 | -0.22030 | 1.697415 | 0.1265 |

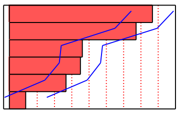

#### Power Details

Test TIME

##### Power

| α | σ | δ | Number | Power |
|---|---|---|---|---|
| 0.0500 | 0.998583 | 2.566677 | 36 | 1.0000 |

Statistical Table 1.
Statistical analysis of data presented in Figure 1, panel B

## Fit Y by X Group

### Oneway Analysis of H3K9m3 By TIME PCR=d/e

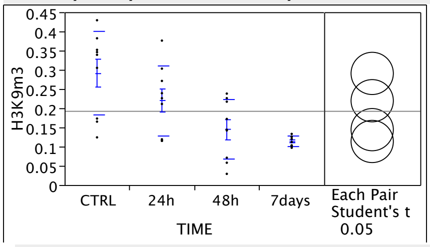


#### Means and Std Deviations

| Level | Number | Mean | Std Dev | Std Err Mean | Lower 95% | Upper 95% |
|---|---|---|---|---|---|---|
| CTRL | 9 | 0.290556 | 0.108546 | 0.03618 | 0.20712 | 0.37399 |
| 24h | 9 | 0.219444 | 0.091209 | 0.03040 | 0.14934 | 0.28955 |
| 48h | 9 | 0.144111 | 0.077158 | 0.02572 | 0.08480 | 0.20342 |
| 7days | 9 | 0.113333 | 0.013048 | 0.00435 | 0.10330 | 0.12336 |

#### Wilcoxon / Kruskal-Wallis Tests (Rank Sums)

| Level | Count | Score Sum | Score Mean | (Mean-Mean0)/Std0 |
|---|---|---|---|---|
| CTRL | 9 | 250.000 | 27.7778 | 3.033 |
| 24h | 9 | 194.500 | 21.6111 | 1.005 |
| 48h | 9 | 134.500 | 14.9444 | -1.151 |
| 7days | 9 | 87.000 | 9.6667 | -2.886 |

#### 1-way Test, ChiSquare Approximation

| ChiSquare | DF | Prob>ChiSq |
|---|---|---|
| 15.1195 | 3 | 0.0017* |

#### Means Comparisons

##### Comparisons for each pair using Student's t

| Level | - Level | Difference | Lower CL | Upper CL | p-Value |
|---|---|---|---|---|---|
| CTRL | 7days | 0.1772222 | 0.099473 | 0.2549717 | <.0001* |
| CTRL | 48h | 0.1464444 | 0.068695 | 0.2241939 | 0.0006* |
| 24h | 7days | 0.1061111 | 0.028362 | 0.1838606 | 0.0090* |
| 24h | 48h | 0.0753333 | -0.002416 | 0.1530828 | 0.0571 |
| CTRL | 24h | 0.0711111 | -0.006638 | 0.1488606 | 0.0717 |
| 48h | 7days | 0.0307778 | -0.046972 | 0.1085273 | 0.4260 |

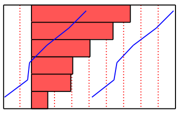

#### Power Details

Test TIME

##### Power

| α | σ | δ | Number | Power |
|---|---|---|---|---|
| 0.0500 | 0.080971 | 0.068826 | 36 | 0.9884 |

### Oneway Analysis of H3K9m2 By TIME PCR=d/e

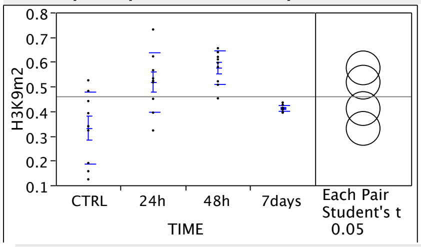


#### Means and Std Deviations

| Level | Number | Mean | Std Dev | Std Err Mean | Lower 95% | Upper 95% |
|---|---|---|---|---|---|---|
| CTRL | 9 | 0.330222 | 0.145518 | 0.04851 | 0.21837 | 0.44208 |
| 24h | 9 | 0.517556 | 0.121014 | 0.04034 | 0.42454 | 0.61057 |
| 48h | 9 | 0.575444 | 0.067888 | 0.02263 | 0.52326 | 0.62763 |
| 7days | 9 | 0.410667 | 0.013086 | 0.00436 | 0.40061 | 0.42073 |

#### Wilcoxon / Kruskal-Wallis Tests (Rank Sums)

| Level | Count | Score Sum | Score Mean | (Mean-Mean0)/Std0 |
|---|---|---|---|---|
| CTRL | 9 | 88.500 | 9.8333 | -2.832 |
| 24h | 9 | 204.000 | 22.6667 | 1.352 |
| 48h | 9 | 257.000 | 28.5556 | 3.289 |
| 7days | 9 | 116.500 | 12.9444 | -1.809 |

#### 1-way Test, ChiSquare Approximation

| ChiSquare | DF | Prob>ChiSq |
|---|---|---|
| 18.2057 | 3 | 0.0004* |

#### Means Comparisons

##### Comparisons for each pair using Student's t

| Level | - Level | Difference | Lower CL | Upper CL | p-Value |
|---|---|---|---|---|---|
| 48h | CTRL | 0.2452222 | 0.148483 | 0.3419614 | <.0001* |
| 24h | CTRL | 0.1873333 | 0.090594 | 0.2840725 | 0.0004* |
| 48h | 7days | 0.1647778 | 0.068039 | 0.2615169 | 0.0015* |
| 24h | 7days | 0.1068889 | 0.010150 | 0.2036280 | 0.0314* |
| 7days | CTRL | 0.0804444 | -0.016295 | 0.1771836 | 0.1000 |
| 48h | 24h | 0.0578889 | -0.038850 | 0.1546280 | 0.2318 |

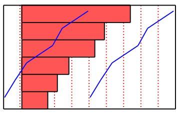

#### Power Details

Test TIME

##### Power

| α | σ | δ | Number | Power |
|---|---|---|---|---|
| 0.0500 | 0.100747 | 0.094745 | 36 | 0.9971 |

Statistical Table 1.
Statistical analysis of data presented in Figure 1, panel B

## Fit Y by X Group

### Oneway Analysis of H3K4m3 By TIME PCR=d/e

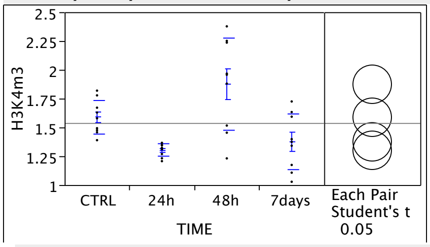


#### Means and Std Deviations

| Level | Number | Mean | Std Dev | Std Err Mean | Lower 95% | Upper 95% |
|---|---|---|---|---|---|---|
| CTRL | 9 | 1.58800 | 0.148401 | 0.04947 | 1.4739 | 1.7021 |
| 24h | 9 | 1.30122 | 0.054295 | 0.01810 | 1.2595 | 1.3430 |
| 48h | 9 | 1.87433 | 0.396826 | 0.13228 | 1.5693 | 2.1794 |
| 7days | 9 | 1.37478 | 0.242618 | 0.08087 | 1.1883 | 1.5613 |

#### Wilcoxon / Kruskal-Wallis Tests (Rank Sums)

| Level | Count | Score Sum | Score Mean | (Mean-Mean0)/Std0 |
|---|---|---|---|---|
| CTRL | 9 | 210.000 | 23.3333 | 1.571 |
| 24h | 9 | 83.000 | 9.2222 | -3.032 |
| 48h | 9 | 246.000 | 27.3333 | 2.886 |
| 7days | 9 | 127.000 | 14.1111 | -1.425 |

#### 1-way Test, ChiSquare Approximation

| ChiSquare | DF | Prob>ChiSq |
|---|---|---|
| 16.7618 | 3 | 0.0008* |

#### Means Comparisons

##### Comparisons for each pair using Student's t

| Level | - Level | Difference | Lower CL | Upper CL | p-Value |
|---|---|---|---|---|---|
| 48h | 24h | 0.5731111 | 0.337268 | 0.8089546 | <.0001* |
| 48h | 7days | 0.4995556 | 0.263712 | 0.7353990 | 0.0001* |
| CTRL | 24h | 0.2867778 | 0.050934 | 0.5226212 | 0.0187* |
| 48h | CTRL | 0.2863333 | 0.050490 | 0.5221768 | 0.0189* |
| CTRL | 7days | 0.2132222 | -0.022621 | 0.4490657 | 0.0748 |
| 7days | 24h | 0.0735556 | -0.162288 | 0.3093990 | 0.5298 |

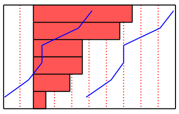

#### Power Details

Test TIME

##### Power

| α | σ | δ | Number | Power |
|---|---|---|---|---|
| 0.0500 | 0.245614 | 0.222642 | 36 | 0.9950 |

### Oneway Analysis of H3K4m2 By TIME PCR=d/e

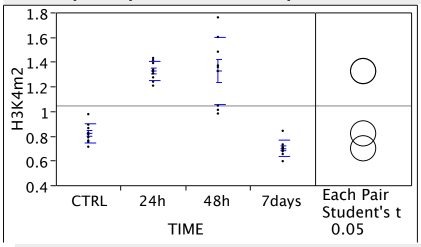


#### Means and Std Deviations

| Level | Number | Mean | Std Dev | Std Err Mean | Lower 95% | Upper 95% |
|---|---|---|---|---|---|---|
| CTRL | 9 | 0.82022 | 0.080526 | 0.02684 | 0.7583 | 0.8821 |
| 24h | 9 | 1.32422 | 0.077320 | 0.02577 | 1.2648 | 1.3837 |
| 48h | 9 | 1.32600 | 0.271242 | 0.09041 | 1.1175 | 1.5345 |
| 7days | 9 | 0.69944 | 0.066099 | 0.02203 | 0.6486 | 0.7503 |

#### Wilcoxon / Kruskal-Wallis Tests (Rank Sums)

| Level | Count | Score Sum | Score Mean | (Mean-Mean0)/Std0 |
|---|---|---|---|---|
| CTRL | 9 | 119.000 | 13.2222 | -1.717 |
| 24h | 9 | 244.000 | 27.1111 | 2.813 |
| 48h | 9 | 251.000 | 27.8889 | 3.069 |
| 7days | 9 | 52.000 | 5.7778 | -4.165 |

#### 1-way Test, ChiSquare Approximation

| ChiSquare | DF | Prob>ChiSq |
|---|---|---|
| 28.5415 | 3 | <.0001* |

#### Means Comparisons

##### Comparisons for each pair using Student's t

| Level | - Level | Difference | Lower CL | Upper CL | p-Value |
|---|---|---|---|---|---|
| 48h | 7days | 0.6265556 | 0.482200 | 0.7709115 | <.0001* |
| 24h | 7days | 0.6247778 | 0.480422 | 0.7691338 | <.0001* |
| 48h | CTRL | 0.5057778 | 0.361422 | 0.6501338 | <.0001* |
| 24h | CTRL | 0.5040000 | 0.359644 | 0.6483560 | <.0001* |
| CTRL | 7days | 0.1207778 | -0.023578 | 0.2651338 | 0.0980 |
| 48h | 24h | 0.0017778 | -0.142578 | 0.1461338 | 0.9801 |

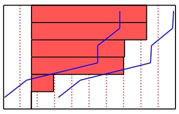

#### Power Details

Test TIME

##### Power

| α | σ | δ | Number | Power |
|---|---|---|---|---|
| 0.0500 | 0.150336 | 0.285847 | 36 | 1.0000 |

## Fit Y by X Group

Statistical Table 1.
Statistical analysis of data presented in Figure 1, panel B

## Fit Y by X Group

### Oneway Analysis of Ig-CTRL By TIME PCR=p/q

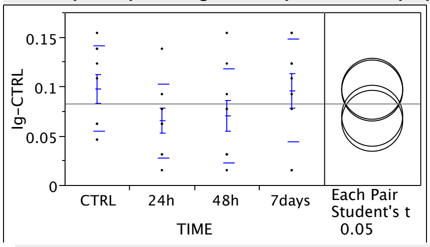


#### Means and Std Deviations

| Level | Number | Mean | Std Dev | Std Err Mean | Lower 95% | Upper 95% |
|---|---|---|---|---|---|---|
| CTRL | 9 | 0.097444 | 0.043385 | 0.01446 | 0.06410 | 0.13079 |
| 24h | 9 | 0.065000 | 0.037376 | 0.01246 | 0.03627 | 0.09373 |
| 48h | 9 | 0.070111 | 0.047480 | 0.01583 | 0.03361 | 0.10661 |
| 7days | 9 | 0.095667 | 0.052235 | 0.01741 | 0.05552 | 0.13582 |

#### Wilcoxon / Kruskal-Wallis Tests (Rank Sums)

| Level | Count | Score Sum | Score Mean | (Mean-Mean0)/Std0 |
|---|---|---|---|---|
| CTRL | 9 | 196.500 | 21.8333 | 1.084 |
| 24h | 9 | 132.500 | 14.7222 | -1.231 |
| 48h | 9 | 143.500 | 15.9444 | -0.827 |
| 7days | 9 | 193.500 | 21.5000 | 0.974 |

#### 1-way Test, ChiSquare Approximation

| ChiSquare | DF | Prob>ChiSq |
|---|---|---|
| 3.3562 | 3 | 0.3399 |

#### Means Comparisons

##### Comparisons for each pair using Student's t

| Level | - Level | Difference | Lower CL | Upper CL | p-Value |
|---|---|---|---|---|---|
| CTRL | 24h | 0.0324444 | -0.011196 | 0.0760847 | 0.1397 |
| 7days | 24h | 0.0306667 | -0.012974 | 0.0743069 | 0.1620 |
| CTRL | 48h | 0.0273333 | -0.016307 | 0.0709735 | 0.2112 |
| 7days | 48h | 0.0255556 | -0.018085 | 0.0691958 | 0.2417 |
| 48h | 24h | 0.0051111 | -0.038529 | 0.0487513 | 0.8130 |
| CTRL | 7days | 0.0017778 | -0.041862 | 0.0454180 | 0.9344 |

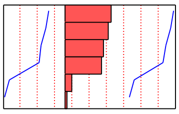

#### Power Details Dialog

TIME
Click and Enter 1, 2 or a sequence of values for each:

| | α | σ | δ | Number |
|---|---|---|---|---|
| From: | 0.050 | 0.045448 | 0.014626 | 36 |
| To: | . | . | . | . |
| By | . | . | . | . |

- ☐ Solve for Power
- ☐ Solve for Least Significant Number
- ☐ Solve for Least Significant Value
- ☐ Adjusted Power and Confidence Interval

Done Cancel Help

Calculations will be done on all combinations of sequences.

### Oneway Analysis of H3 By TIME PCR=p/q

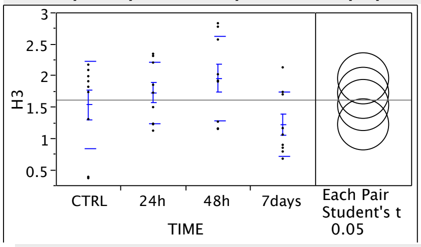


#### Means and Std Deviations

| Level | Number | Mean | Std Dev | Std Err Mean | Lower 95% | Upper 95% |
|---|---|---|---|---|---|---|
| CTRL | 9 | 1.52589 | 0.699425 | 0.23314 | 0.9883 | 2.0635 |
| 24h | 9 | 1.71967 | 0.485177 | 0.16173 | 1.3467 | 2.0926 |
| 48h | 9 | 1.95044 | 0.670150 | 0.22338 | 1.4353 | 2.4656 |
| 7days | 9 | 1.21811 | 0.509412 | 0.16980 | 0.8265 | 1.6097 |

#### Wilcoxon / Kruskal-Wallis Tests (Rank Sums)

| Level | Count | Score Sum | Score Mean | (Mean-Mean0)/Std0 |
|---|---|---|---|---|
| CTRL | 9 | 166.000 | 18.4444 | -0.000 |
| 24h | 9 | 185.000 | 20.5556 | 0.658 |
| 48h | 9 | 213.000 | 23.6667 | 1.681 |
| 7days | 9 | 102.000 | 11.3333 | -2.338 |

#### 1-way Test, ChiSquare Approximation

| ChiSquare | DF | Prob>ChiSq |
|---|---|---|
| 6.6717 | 3 | 0.0831 |

#### Means Comparisons

##### Comparisons for each pair using Student's t

| Level | - Level | Difference | Lower CL | Upper CL | p-Value |
|---|---|---|---|---|---|
| 48h | 7days | 0.7323333 | 0.157565 | 1.307102 | 0.0141* |
| 24h | 7days | 0.5015556 | -0.073213 | 1.076324 | 0.0850 |
| 48h | CTRL | 0.4245556 | -0.150213 | 0.999324 | 0.1422 |
| CTRL | 7days | 0.3077778 | -0.266991 | 0.882546 | 0.2835 |
| 48h | 24h | 0.2307778 | -0.343991 | 0.805546 | 0.4195 |
| 24h | CTRL | 0.1937778 | -0.380991 | 0.768546 | 0.4972 |

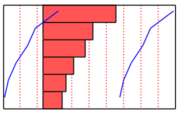

#### Power Details Dialog

TIME
Click and Enter 1, 2 or a sequence of values for each:

| | α | σ | δ | Number |
|---|---|---|---|---|
| From: | 0.050 | 0.59858 | 0.268521 | 36 |
| To: | . | . | . | . |
| By | . | . | . | . |

- ☐ Solve for Power
- ☐ Solve for Least Significant Number
- ☐ Solve for Least Significant Value
- ☐ Adjusted Power and Confidence Interval

Done Cancel Help

Calculations will be done on all combinations of sequences.

Statistical Table 1.

Statistical analysis of data presented in Figure 1, panel B

## Fit Y by X Group

### Oneway Analysis of H3K9m3 By TIME PCR=p/q

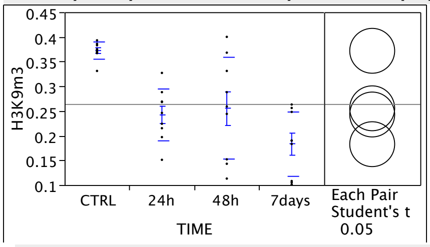


#### Means and Std Deviations

| Level | Number | Mean | Std Dev | Std Err Mean | Lower 95% | Upper 95% |
|---|---|---|---|---|---|---|
| CTRL | 9 | 0.371778 | 0.017655 | 0.00588 | 0.35821 | 0.38535 |
| 24h | 9 | 0.242778 | 0.052578 | 0.01753 | 0.20236 | 0.28319 |
| 48h | 9 | 0.255333 | 0.102538 | 0.03418 | 0.17652 | 0.33415 |
| 7days | 9 | 0.182667 | 0.065654 | 0.02188 | 0.13220 | 0.23313 |

#### Wilcoxon / Kruskal-Wallis Tests (Rank Sums)

| Level | Count | Score Sum | Score Mean | (Mean-Mean0)/Std0 |
|---|---|---|---|---|
| CTRL | 9 | 275.500 | 30.6111 | 3.965 |
| 24h | 9 | 144.500 | 16.0556 | -0.786 |
| 48h | 9 | 161.000 | 17.8889 | -0.183 |
| 7days | 9 | 85.000 | 9.4444 | -2.960 |

#### 1-way Test, ChiSquare Approximation

| ChiSquare | DF | Prob>ChiSq |
|---|---|---|
| 19.0639 | 3 | 0.0003* |

#### Means Comparisons

##### Comparisons for each pair using Student's t

| Level | - Level | Difference | Lower CL | Upper CL | p-Value |
|---|---|---|---|---|---|
| CTRL | 7days | 0.1891111 | 0.124876 | 0.2533466 | <.0001* |
| CTRL | 24h | 0.1290000 | 0.064765 | 0.1932355 | 0.0003* |
| CTRL | 48h | 0.1164444 | 0.052209 | 0.1806799 | 0.0008* |
| 48h | 7days | 0.0726667 | 0.008431 | 0.1369021 | 0.0278* |
| 24h | 7days | 0.0601111 | -0.004124 | 0.1243466 | 0.0656 |
| 48h | 24h | 0.0125556 | -0.051680 | 0.0767910 | 0.6932 |

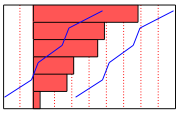

#### Power Details Dialog

TIME
Click and Enter 1, 2 or a sequence of values for each:

| | α | σ | δ | Number |
|---|---|---|---|---|
| From: | 0.050 | 0.066897 | 0.068472 | 36 |
| To: | . | . | . | . |
| By | . | . | . | . |

- ☐ Solve for Power
- ☐ Solve for Least Significant Number
- ☐ Solve for Least Significant Value
- ☐ Adjusted Power and Confidence Interval

Done Cancel Help

Calculations will be done on all combinations of sequences.

### Oneway Analysis of H3K9m2 By TIME PCR=p/q

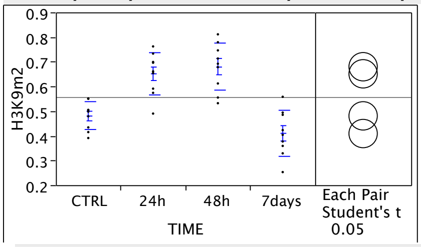


#### Means and Std Deviations

| Level | Number | Mean | Std Dev | Std Err Mean | Lower 95% | Upper 95% |
|---|---|---|---|---|---|---|
| CTRL | 9 | 0.480778 | 0.056705 | 0.01890 | 0.43719 | 0.52437 |
| 24h | 9 | 0.651111 | 0.085829 | 0.02861 | 0.58514 | 0.71709 |
| 48h | 9 | 0.679778 | 0.096636 | 0.03221 | 0.60550 | 0.75406 |
| 7days | 9 | 0.409111 | 0.093329 | 0.03111 | 0.33737 | 0.48085 |

#### Wilcoxon / Kruskal-Wallis Tests (Rank Sums)

| Level | Count | Score Sum | Score Mean | (Mean-Mean0)/Std0 |
|---|---|---|---|---|
| CTRL | 9 | 113.000 | 12.5556 | -1.936 |
| 24h | 9 | 233.000 | 25.8889 | 2.411 |
| 48h | 9 | 251.000 | 27.8889 | 3.069 |
| 7days | 9 | 69.000 | 7.6667 | -3.544 |

#### 1-way Test, ChiSquare Approximation

| ChiSquare | DF | Prob>ChiSq |
|---|---|---|
| 23.9550 | 3 | <.0001* |

#### Means Comparisons

##### Comparisons for each pair using Student's t

| Level | - Level | Difference | Lower CL | Upper CL | p-Value |
|---|---|---|---|---|---|
| 48h | 7days | 0.2706667 | 0.189429 | 0.3519044 | <.0001* |
| 24h | 7days | 0.2420000 | 0.160762 | 0.3232377 | <.0001* |
| 48h | CTRL | 0.1990000 | 0.117762 | 0.2802377 | <.0001* |
| 24h | CTRL | 0.1703333 | 0.089096 | 0.2515711 | 0.0002* |
| CTRL | 7days | 0.0716667 | -0.009571 | 0.1529044 | 0.0818 |
| 48h | 24h | 0.0286667 | -0.052571 | 0.1099044 | 0.4775 |

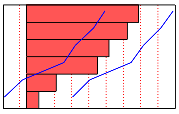

#### Power Details Dialog

TIME
Click and Enter 1, 2 or a sequence of values for each:

| | α | σ | δ | Number |
|---|---|---|---|---|
| From: | 0.050 | 0.084603 | 0.113577 | 36 |
| To: | . | . | . | . |
| By | . | . | . | . |

- ☐ Solve for Power
- ☐ Solve for Least Significant Number
- ☐ Solve for Least Significant Value
- ☐ Adjusted Power and Confidence Interval

Done Cancel Help

Calculations will be done on all combinations of sequences.

Statistical Table 1.
Statistical analysis of data presented in Figure 1, panel B

## Fit Y by X Group

### Oneway Analysis of H3K4m3 By TIME PCR=p/q

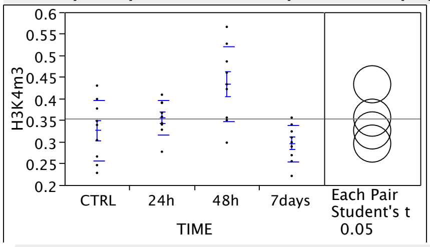


#### Means and Std Deviations

| Level | Number | Mean | Std Dev | Std Err Mean | Lower 95% | Upper 95% |
|---|---|---|---|---|---|---|
| CTRL | 9 | 0.326333 | 0.070255 | 0.02342 | 0.27233 | 0.38034 |
| 24h | 9 | 0.355889 | 0.039945 | 0.01332 | 0.32518 | 0.38659 |
| 48h | 9 | 0.433111 | 0.087483 | 0.02916 | 0.36587 | 0.50036 |
| 7days | 9 | 0.295889 | 0.042733 | 0.01424 | 0.26304 | 0.32874 |

#### Wilcoxon / Kruskal-Wallis Tests (Rank Sums)

| Level | Count | Score Sum | Score Mean | (Mean-Mean0)/Std0 |
|---|---|---|---|---|
| CTRL | 9 | 139.000 | 15.4444 | -0.987 |
| 24h | 9 | 184.500 | 20.5000 | 0.639 |
| 48h | 9 | 250.500 | 27.8333 | 3.051 |
| 7days | 9 | 92.000 | 10.2222 | -2.704 |

#### 1-way Test, ChiSquare Approximation

| ChiSquare | DF | Prob>ChiSq |
|---|---|---|
| 13.7037 | 3 | 0.0033* |

#### Means Comparisons

##### Comparisons for each pair using Student's t

| Level | - Level | Difference | Lower CL | Upper CL | p-Value |
|---|---|---|---|---|---|
| 48h | 7days | 0.1372222 | 0.076472 | 0.1979724 | <.0001* |
| 48h | CTRL | 0.1067778 | 0.046028 | 0.1675280 | 0.0011* |
| 48h | 24h | 0.0772222 | 0.016472 | 0.1379724 | 0.0144* |
| 24h | 7days | 0.0600000 | -0.000750 | 0.1207502 | 0.0527 |
| CTRL | 7days | 0.0304444 | -0.030306 | 0.0911946 | 0.3150 |
| 24h | CTRL | 0.0295556 | -0.031195 | 0.0903057 | 0.3291 |

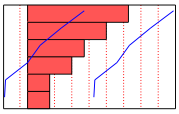

#### Power Details

Test TIME

##### Power

| α | σ | δ | Number | Power |
|---|---|---|---|---|
| 0.0500 | 0.063267 | 0.050987 | 36 | 0.9788 |

### Oneway Analysis of H3K4m2 By TIME PCR=p/q

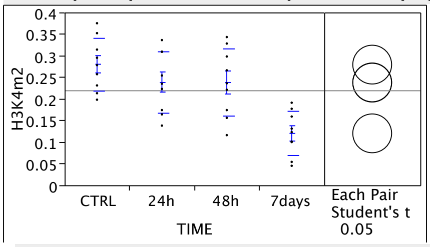


#### Means and Std Deviations

| Level | Number | Mean | Std Dev | Std Err Mean | Lower 95% | Upper 95% |
|---|---|---|---|---|---|---|
| CTRL | 9 | 0.279222 | 0.060963 | 0.02032 | 0.23236 | 0.32608 |
| 24h | 9 | 0.238000 | 0.070488 | 0.02350 | 0.18382 | 0.29218 |
| 48h | 9 | 0.237556 | 0.078664 | 0.02622 | 0.17709 | 0.29802 |
| 7days | 9 | 0.120111 | 0.050846 | 0.01695 | 0.08103 | 0.15920 |

#### Wilcoxon / Kruskal-Wallis Tests (Rank Sums)

| Level | Count | Score Sum | Score Mean | (Mean-Mean0)/Std0 |
|---|---|---|---|---|
| CTRL | 9 | 232.000 | 25.7778 | 2.375 |
| 24h | 9 | 186.500 | 20.7222 | 0.712 |
| 48h | 9 | 185.500 | 20.6111 | 0.676 |
| 7days | 9 | 62.000 | 6.8889 | -3.800 |

#### 1-way Test, ChiSquare Approximation

| ChiSquare | DF | Prob>ChiSq |
|---|---|---|
| 15.9916 | 3 | 0.0011* |

#### Means Comparisons

##### Comparisons for each pair using Student's t

| Level | - Level | Difference | Lower CL | Upper CL | p-Value |
|---|---|---|---|---|---|
| CTRL | 7days | 0.1591111 | 0.095674 | 0.2225480 | <.0001* |
| 24h | 7days | 0.1178889 | 0.054452 | 0.1813258 | 0.0006* |
| 48h | 7days | 0.1174444 | 0.054008 | 0.1808813 | 0.0007* |
| CTRL | 48h | 0.0416667 | -0.021770 | 0.1051036 | 0.1904 |
| CTRL | 24h | 0.0412222 | -0.022215 | 0.1046591 | 0.1950 |
| 24h | 48h | 0.0004444 | -0.062992 | 0.0638813 | 0.9887 |

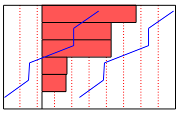

#### Power Details

Test TIME

##### Power

| α | σ | δ | Number | Power |
|---|---|---|---|---|
| 0.0500 | 0.066065 | 0.059394 | 36 | 0.9944 |

## Fit Y by X Group

Statistical Table 1.
Statistical analysis of data presented in Figure 1, panel B

## Fit Y by X Group

### Oneway Analysis of Ig-CTRL By TIME PCR=r/h

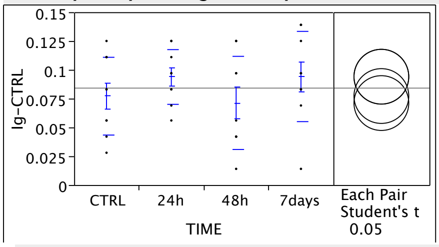


#### Means and Std Deviations

| Level | Number | Mean | Std Dev | Std Err Mean | Lower 95% | Upper 95% |
|---|---|---|---|---|---|---|
| CTRL | 9 | 0.077222 | 0.033903 | 0.01130 | 0.05116 | 0.10328 |
| 24h | 9 | 0.094000 | 0.023822 | 0.00794 | 0.07569 | 0.11231 |
| 48h | 9 | 0.071111 | 0.040591 | 0.01353 | 0.03991 | 0.10231 |
| 7days | 9 | 0.094000 | 0.039205 | 0.01307 | 0.06386 | 0.12414 |

#### Wilcoxon / Kruskal-Wallis Tests (Rank Sums)

| Level | Count | Score Sum | Score Mean | (Mean-Mean0)/Std0 |
|---|---|---|---|---|
| CTRL | 9 | 146.000 | 16.2222 | -0.737 |
| 24h | 9 | 190.000 | 21.1111 | 0.847 |
| 48h | 9 | 135.500 | 15.0556 | -1.124 |
| 7days | 9 | 194.500 | 21.6111 | 1.013 |

#### 1-way Test, ChiSquare Approximation

| ChiSquare | DF | Prob>ChiSq |
|---|---|---|
| 2.7672 | 3 | 0.4289 |

#### Means Comparisons

##### Comparisons for each pair using Student's t

| Level | - Level | Difference | Lower CL | Upper CL | p-Value |
|---|---|---|---|---|---|
| 7days | 48h | 0.0228889 | -0.010724 | 0.0565019 | 0.1750 |
| 24h | 48h | 0.0228889 | -0.010724 | 0.0565019 | 0.1750 |
| 7days | CTRL | 0.0167778 | -0.016835 | 0.0503908 | 0.3169 |
| 24h | CTRL | 0.0167778 | -0.016835 | 0.0503908 | 0.3169 |
| CTRL | 48h | 0.0061111 | -0.027502 | 0.0397241 | 0.7136 |
| 7days | 24h | 1.388e-17 | -0.033613 | 0.0336130 | 1.0000 |

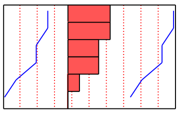

#### Power Details

Test TIME

##### Power

| α | σ | δ | Number | Power |
|---|---|---|---|---|
| 0.0500 | 0.035006 | 0.010149 | 36 | 0.2483 |

### Oneway Analysis of H3 By TIME PCR=r/h

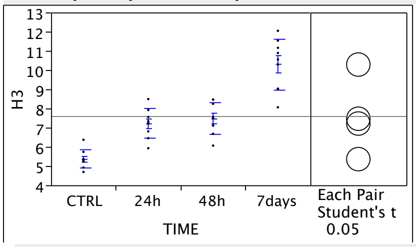


#### Means and Std Deviations

| Level | Number | Mean | Std Dev | Std Err Mean | Lower 95% | Upper 95% |
|---|---|---|---|---|---|---|
| CTRL | 9 | 5.3603 | 0.47795 | 0.15932 | 4.9930 | 5.728 |
| 24h | 9 | 7.2130 | 0.76281 | 0.25427 | 6.6267 | 7.799 |
| 48h | 9 | 7.4640 | 0.82288 | 0.27429 | 6.8315 | 8.097 |
| 7days | 9 | 10.2850 | 1.32165 | 0.44055 | 9.2691 | 11.301 |

#### Wilcoxon / Kruskal-Wallis Tests (Rank Sums)

| Level | Count | Score Sum | Score Mean | (Mean-Mean0)/Std0 |
|---|---|---|---|---|
| CTRL | 9 | 47.000 | 5.2222 | -4.347 |
| 24h | 9 | 162.000 | 18.0000 | -0.146 |
| 48h | 9 | 173.000 | 19.2222 | 0.219 |
| 7days | 9 | 284.000 | 31.5556 | 4.274 |

#### 1-way Test, ChiSquare Approximation

| ChiSquare | DF | Prob>ChiSq |
|---|---|---|
| 28.1772 | 3 | <.0001* |

#### Means Comparisons

##### Comparisons for each pair using Student's t

| Level | - Level | Difference | Lower CL | Upper CL | p-Value |
|---|---|---|---|---|---|
| 7days | CTRL | 4.924667 | 4.06124 | 5.788091 | <.0001* |
| 7days | 24h | 3.072000 | 2.20858 | 3.935424 | <.0001* |
| 7days | 48h | 2.821000 | 1.95758 | 3.684424 | <.0001* |
| 48h | CTRL | 2.103667 | 1.24024 | 2.967091 | <.0001* |
| 24h | CTRL | 1.852667 | 0.98924 | 2.716091 | 0.0001* |
| 48h | 24h | 0.251000 | -0.61242 | 1.114424 | 0.5579 |

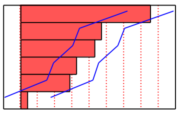

#### Power Details

Test TIME

##### Power

| α | σ | δ | Number | Power |
|---|---|---|---|---|
| 0.0500 | 0.899195 | 1.76012 | 36 | 1.0000 |

Statistical Table 1.
Statistical analysis of data presented in Figure 1, panel B

## Fit Y by X Group

### Oneway Analysis of H3K9m3 By TIME PCR=r/h

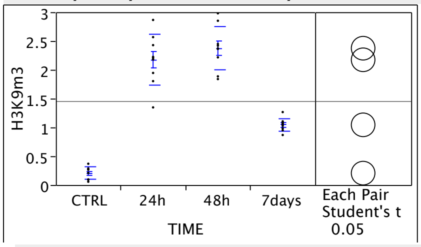


#### Means and Std Deviations

| Level | Number | Mean | Std Dev | Std Err Mean | Lower 95% | Upper 95% |
|---|---|---|---|---|---|---|
| CTRL | 9 | 0.20600 | 0.105134 | 0.03504 | 0.1252 | 0.2868 |
| 24h | 9 | 2.17356 | 0.443556 | 0.14785 | 1.8326 | 2.5145 |
| 48h | 9 | 2.37478 | 0.379631 | 0.12654 | 2.0830 | 2.6666 |
| 7days | 9 | 1.04444 | 0.111220 | 0.03707 | 0.9590 | 1.1299 |

#### Wilcoxon / Kruskal-Wallis Tests (Rank Sums)

| Level | Count | Score Sum | Score Mean | (Mean-Mean0)/Std0 |
|---|---|---|---|---|
| CTRL | 9 | 45.000 | 5.0000 | -4.421 |
| 24h | 9 | 237.000 | 26.3333 | 2.557 |
| 48h | 9 | 258.000 | 28.6667 | 3.325 |
| 7days | 9 | 126.000 | 14.0000 | -1.461 |

#### 1-way Test, ChiSquare Approximation

| ChiSquare | DF | Prob>ChiSq |
|---|---|---|
| 29.7748 | 3 | <.0001* |

#### Means Comparisons

##### Comparisons for each pair using Student's t

| Level | - Level | Difference | Lower CL | Upper CL | p-Value |
|---|---|---|---|---|---|
| 48h | CTRL | 2.168778 | 1.87900 | 2.458553 | <.0001* |
| 24h | CTRL | 1.967556 | 1.67778 | 2.257331 | <.0001* |
| 48h | 7days | 1.330333 | 1.04056 | 1.620108 | <.0001* |
| 24h | 7days | 1.129111 | 0.83934 | 1.418886 | <.0001* |
| 7days | CTRL | 0.838444 | 0.54867 | 1.128219 | <.0001* |
| 48h | 24h | 0.201222 | -0.08855 | 0.490997 | 0.1669 |

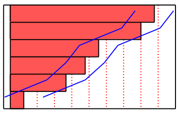

#### Power Details

Test TIME

##### Power

| α | σ | δ | Number | Power |
|---|---|---|---|---|
| 0.0500 | 0.30178 | 0.879028 | 36 | 1.0000 |

### Oneway Analysis of H3K9m2 By TIME PCR=r/h

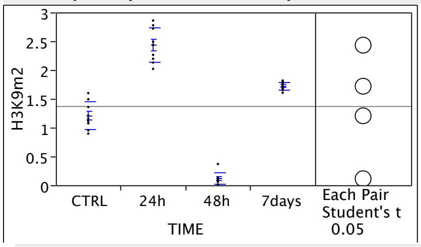


#### Means and Std Deviations

| Level | Number | Mean | Std Dev | Std Err Mean | Lower 95% | Upper 95% |
|---|---|---|---|---|---|---|
| CTRL | 9 | 1.20578 | 0.236716 | 0.07891 | 1.0238 | 1.3877 |
| 24h | 9 | 2.43189 | 0.302574 | 0.10086 | 2.1993 | 2.6645 |
| 48h | 9 | 0.11289 | 0.101718 | 0.03391 | 0.0347 | 0.1911 |
| 7days | 9 | 1.71922 | 0.070892 | 0.02363 | 1.6647 | 1.7737 |

#### Wilcoxon / Kruskal-Wallis Tests (Rank Sums)

| Level | Count | Score Sum | Score Mean | (Mean-Mean0)/Std0 |
|---|---|---|---|---|
| CTRL | 9 | 126.000 | 14.0000 | -1.461 |
| 24h | 9 | 288.000 | 32.0000 | 4.421 |
| 48h | 9 | 45.000 | 5.0000 | -4.421 |
| 7days | 9 | 207.000 | 23.0000 | 1.461 |

#### 1-way Test, ChiSquare Approximation

| ChiSquare | DF | Prob>ChiSq |
|---|---|---|
| 32.8421 | 3 | <.0001* |

#### Means Comparisons

##### Comparisons for each pair using Student's t

| Level | - Level | Difference | Lower CL | Upper CL | p-Value |
|---|---|---|---|---|---|
| 24h | 48h | 2.319000 | 2.125189 | 2.512811 | <.0001* |
| 7days | 48h | 1.606333 | 1.412522 | 1.800144 | <.0001* |
| 24h | CTRL | 1.226111 | 1.032300 | 1.419922 | <.0001* |
| CTRL | 48h | 1.092889 | 0.899078 | 1.286700 | <.0001* |
| 24h | 7days | 0.712667 | 0.518856 | 0.906478 | <.0001* |
| 7days | CTRL | 0.513444 | 0.319633 | 0.707256 | <.0001* |

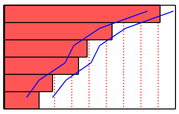

#### Power Details

Test TIME

##### Power

| α | σ | δ | Number | Power |
|---|---|---|---|---|
| 0.0500 | 0.20184 | 0.845109 | 36 | 1.0000 |

Statistical Table 1.
Statistical analysis of data presented in Figure 1, panel B

## Fit Y by X Group

### Oneway Analysis of H3K4m3 By TIME PCR=r/h

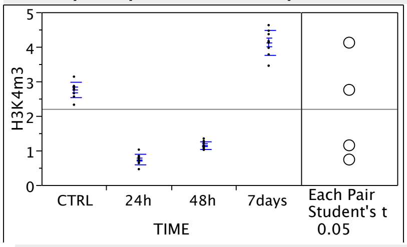


#### Means and Std Deviations

| Level | Number | Mean | Std Dev | Std Err Mean | Lower 95% | Upper 95% |
|---|---|---|---|---|---|---|
| CTRL | 9 | 2.75267 | 0.225884 | 0.07529 | 2.5790 | 2.9263 |
| 24h | 9 | 0.73600 | 0.156503 | 0.05217 | 0.6157 | 0.8563 |
| 48h | 9 | 1.14833 | 0.111155 | 0.03705 | 1.0629 | 1.2338 |
| 7days | 9 | 4.11833 | 0.357359 | 0.11912 | 3.8436 | 4.3930 |

#### Wilcoxon / Kruskal-Wallis Tests (Rank Sums)

| Level | Count | Score Sum | Score Mean | (Mean-Mean0)/Std0 |
|---|---|---|---|---|
| CTRL | 9 | 207.000 | 23.0000 | 1.461 |
| 24h | 9 | 46.000 | 5.1111 | -4.384 |
| 48h | 9 | 125.000 | 13.8889 | -1.498 |
| 7days | 9 | 288.000 | 32.0000 | 4.421 |

#### 1-way Test, ChiSquare Approximation

| ChiSquare | DF | Prob>ChiSq |
|---|---|---|
| 32.6777 | 3 | <.0001* |

#### Means Comparisons

##### Comparisons for each pair using Student's t

| Level | - Level | Difference | Lower CL | Upper CL | p-Value |
|---|---|---|---|---|---|
| 7days | 24h | 3.382333 | 3.159416 | 3.605250 | <.0001* |
| 7days | 48h | 2.970000 | 2.747083 | 3.192917 | <.0001* |
| CTRL | 24h | 2.016667 | 1.793750 | 2.239584 | <.0001* |
| CTRL | 48h | 1.604333 | 1.381416 | 1.827250 | <.0001* |
| 7days | CTRL | 1.365667 | 1.142750 | 1.588584 | <.0001* |
| 48h | 24h | 0.412333 | 0.189416 | 0.635250 | 0.0007* |

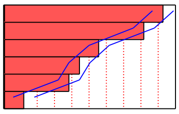

#### Power Details

Test TIME

##### Power

| α | σ | δ | Number | Power |
|---|---|---|---|---|
| 0.0500 | 0.232152 | 1.344827 | 36 | 1.0000 |

### Oneway Analysis of H3K4m2 By TIME PCR=r/h

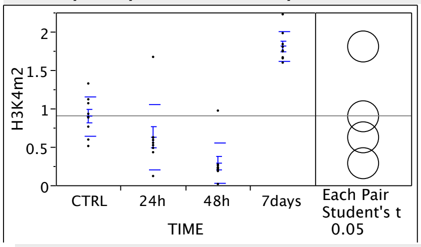


#### Means and Std Deviations

| Level | Number | Mean | Std Dev | Std Err Mean | Lower 95% | Upper 95% |
|---|---|---|---|---|---|---|
| CTRL | 9 | 0.89789 | 0.255258 | 0.08509 | 0.7017 | 1.0941 |
| 24h | 9 | 0.62400 | 0.421840 | 0.14061 | 0.2997 | 0.9483 |
| 48h | 9 | 0.28689 | 0.267571 | 0.08919 | 0.0812 | 0.4926 |
| 7days | 9 | 1.80700 | 0.194031 | 0.06468 | 1.6579 | 1.9561 |

#### Wilcoxon / Kruskal-Wallis Tests (Rank Sums)

| Level | Count | Score Sum | Score Mean | (Mean-Mean0)/Std0 |
|---|---|---|---|---|
| CTRL | 9 | 185.000 | 20.5556 | 0.658 |
| 24h | 9 | 130.000 | 14.4444 | -1.315 |
| 48h | 9 | 66.000 | 7.3333 | -3.653 |
| 7days | 9 | 285.000 | 31.6667 | 4.311 |

#### 1-way Test, ChiSquare Approximation

| ChiSquare | DF | Prob>ChiSq |
|---|---|---|
| 25.8428 | 3 | <.0001* |

#### Means Comparisons

##### Comparisons for each pair using Student's t

| Level | - Level | Difference | Lower CL | Upper CL | p-Value |
|---|---|---|---|---|---|
| 7days | 48h | 1.520111 | 1.23512 | 1.805099 | <.0001* |
| 7days | 24h | 1.183000 | 0.89801 | 1.467988 | <.0001* |
| 7days | CTRL | 0.909111 | 0.62412 | 1.194099 | <.0001* |
| CTRL | 48h | 0.611000 | 0.32601 | 0.895988 | 0.0001* |
| 24h | 48h | 0.337111 | 0.05212 | 0.622099 | 0.0219* |
| CTRL | 24h | 0.273889 | -0.01110 | 0.558877 | 0.0590 |

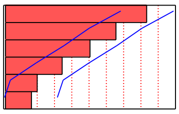

#### Power Details

Test TIME

##### Power

| α | σ | δ | Number | Power |
|---|---|---|---|---|
| 0.0500 | 0.296794 | 0.564507 | 36 | 1.0000 |

## Fit Y by X Group

Statistical Table 1.
Statistical analysis of data presented in Figure 1, panel B

## Fit Y by X Group

### Oneway Analysis of Ig-CTRL By TIME PCR=Tf/Tr

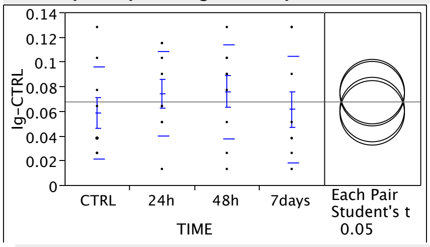


**Means and Std Deviations**

| Level | Number | Mean | Std Dev | Std Err Mean | Lower 95% | Upper 95% |
|---|---|---|---|---|---|---|
| CTRL | 9 | 0.058444 | 0.037404 | 0.01247 | 0.02969 | 0.08720 |
| 24h | 9 | 0.074000 | 0.034347 | 0.01145 | 0.04760 | 0.10040 |
| 48h | 9 | 0.075667 | 0.038158 | 0.01272 | 0.04634 | 0.10500 |
| 7days | 9 | 0.061111 | 0.043352 | 0.01445 | 0.02779 | 0.09443 |

**Wilcoxon / Kruskal-Wallis Tests (Rank Sums)**

| Level | Count | Score Sum | Score Mean | (Mean-Mean0)/Std0 |
|---|---|---|---|---|
| CTRL | 9 | 143.500 | 15.9444 | -0.827 |
| 24h | 9 | 187.000 | 20.7778 | 0.735 |
| 48h | 9 | 185.500 | 20.6111 | 0.680 |
| 7days | 9 | 150.000 | 16.6667 | -0.588 |

**1-way Test, ChiSquare Approximation**

| ChiSquare | DF | Prob>ChiSq |
|---|---|---|
| 1.6026 | 3 | 0.6588 |

**Means Comparisons**

**Comparisons for each pair using Student's t**

| Level | - Level | Difference | Lower CL | Upper CL | p-Value |
|---|---|---|---|---|---|
| 48h | CTRL | 0.0172222 | -0.019700 | 0.0541443 | 0.3492 |
| 24h | CTRL | 0.0155556 | -0.021367 | 0.0524776 | 0.3972 |
| 48h | 7days | 0.0145556 | -0.022367 | 0.0514776 | 0.4279 |
| 24h | 7days | 0.0128889 | -0.024033 | 0.0498110 | 0.4822 |
| 7days | CTRL | 0.0026667 | -0.034255 | 0.0395888 | 0.8840 |
| 48h | 24h | 0.0016667 | -0.035255 | 0.0385888 | 0.9273 |

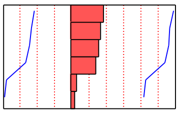

**Power Details**

Test TIME

**Power**

| α | σ | δ | Number | Power |
|---|---|---|---|---|
| 0.0500 | 0.038452 | 0.007609 | 36 | 0.1341 |

### Oneway Analysis of H3 By TIME PCR=Tf/Tr

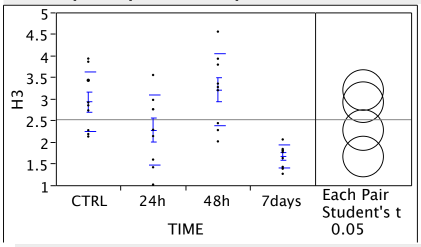


**Means and Std Deviations**

| Level | Number | Mean | Std Dev | Std Err Mean | Lower 95% | Upper 95% |
|---|---|---|---|---|---|---|
| CTRL | 9 | 2.92289 | 0.688267 | 0.22942 | 2.3938 | 3.4519 |
| 24h | 9 | 2.27722 | 0.821222 | 0.27374 | 1.6460 | 2.9085 |
| 48h | 9 | 3.20344 | 0.835733 | 0.27858 | 2.5610 | 3.8458 |
| 7days | 9 | 1.66478 | 0.258868 | 0.08629 | 1.4658 | 1.8638 |

**Wilcoxon / Kruskal-Wallis Tests (Rank Sums)**

| Level | Count | Score Sum | Score Mean | (Mean-Mean0)/Std0 |
|---|---|---|---|---|
| CTRL | 9 | 215.000 | 23.8889 | 1.754 |
| 24h | 9 | 147.000 | 16.3333 | -0.694 |
| 48h | 9 | 236.000 | 26.2222 | 2.521 |
| 7days | 9 | 68.000 | 7.5556 | -3.580 |

**1-way Test, ChiSquare Approximation**

| ChiSquare | DF | Prob>ChiSq |
|---|---|---|
| 17.2823 | 3 | 0.0006* |

**Means Comparisons**

**Comparisons for each pair using Student's t**

| Level | - Level | Difference | Lower CL | Upper CL | p-Value |
|---|---|---|---|---|---|
| 48h | 7days | 1.538667 | 0.874520 | 2.202813 | <.0001* |
| CTRL | 7days | 1.258111 | 0.593965 | 1.922257 | 0.0005* |
| 48h | 24h | 0.926222 | 0.262076 | 1.590369 | 0.0078* |
| CTRL | 24h | 0.645667 | -0.018480 | 1.309813 | 0.0563 |
| 24h | 7days | 0.612444 | -0.051702 | 1.276591 | 0.0695 |
| 48h | CTRL | 0.280556 | -0.383591 | 0.944702 | 0.3959 |

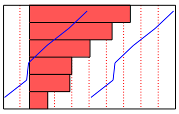

**Power Details**

Test TIME

**Power**

| α | σ | δ | Number | Power |
|---|---|---|---|---|
| 0.0500 | 0.691661 | 0.595762 | 36 | 0.9901 |

Statistical Table 1.
Statistical analysis of data presented in Figure 1, panel B

## Fit Y by X Group

### Oneway Analysis of H3K9m3 By TIME PCR=Tf/Tr

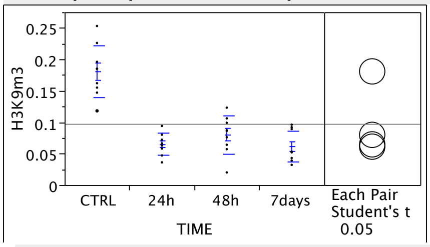


#### Means and Std Deviations

| Level | Number | Mean | Std Dev | Std Err Mean | Lower 95% | Upper 95% |
|---|---|---|---|---|---|---|
| CTRL | 9 | 0.180778 | 0.041273 | 0.01376 | 0.14905 | 0.21250 |
| 24h | 9 | 0.064667 | 0.017081 | 0.00569 | 0.05154 | 0.07780 |
| 48h | 9 | 0.079778 | 0.030355 | 0.01012 | 0.05644 | 0.10311 |
| 7days | 9 | 0.061000 | 0.024566 | 0.00819 | 0.04212 | 0.07988 |

#### Wilcoxon / Kruskal-Wallis Tests (Rank Sums)

| Level | Count | Score Sum | Score Mean | (Mean-Mean0)/Std0 |
|---|---|---|---|---|
| CTRL | 9 | 287.000 | 31.8889 | 4.385 |
| 24h | 9 | 118.000 | 13.1111 | -1.754 |
| 48h | 9 | 159.000 | 17.6667 | -0.256 |
| 7days | 9 | 102.000 | 11.3333 | -2.339 |

#### 1-way Test, ChiSquare Approximation

| ChiSquare | DF | Prob>ChiSq |
|---|---|---|
| 21.1183 | 3 | <.0001* |

#### Means Comparisons

##### Comparisons for each pair using Student's t

| Level | - Level | Difference | Lower CL | Upper CL | p-Value |
|---|---|---|---|---|---|
| CTRL | 7days | 0.1197778 | 0.091292 | 0.1482631 | <.0001* |
| CTRL | 24h | 0.1161111 | 0.087626 | 0.1445964 | <.0001* |
| CTRL | 48h | 0.1010000 | 0.072515 | 0.1294853 | <.0001* |
| 48h | 7days | 0.0187778 | -0.009708 | 0.0472631 | 0.1888 |
| 48h | 24h | 0.0151111 | -0.013374 | 0.0435964 | 0.2880 |
| 24h | 7days | 0.0036667 | -0.024819 | 0.0321519 | 0.7949 |

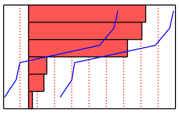

#### Power Details

Test TIME

##### Power

| α | σ | δ | Number | Power |
|---|---|---|---|---|
| 0.0500 | 0.029665 | 0.049132 | 36 | 1.0000 |

### Oneway Analysis of H3K9m2 By TIME PCR=Tf/Tr

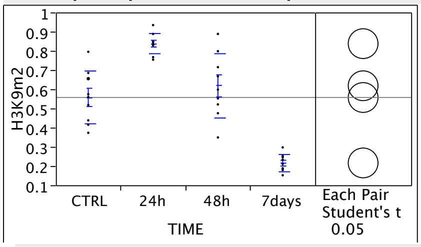


#### Means and Std Deviations

| Level | Number | Mean | Std Dev | Std Err Mean | Lower 95% | Upper 95% |
|---|---|---|---|---|---|---|
| CTRL | 9 | 0.556889 | 0.138124 | 0.04604 | 0.45072 | 0.66306 |
| 24h | 9 | 0.837444 | 0.054850 | 0.01828 | 0.79528 | 0.87961 |
| 48h | 9 | 0.618111 | 0.167364 | 0.05579 | 0.48946 | 0.74676 |
| 7days | 9 | 0.216222 | 0.045047 | 0.01502 | 0.18160 | 0.25085 |

#### Wilcoxon / Kruskal-Wallis Tests (Rank Sums)

| Level | Count | Score Sum | Score Mean | (Mean-Mean0)/Std0 |
|---|---|---|---|---|
| CTRL | 9 | 159.000 | 17.6667 | -0.256 |
| 24h | 9 | 276.500 | 30.7222 | 4.001 |
| 48h | 9 | 185.500 | 20.6111 | 0.676 |
| 7days | 9 | 45.000 | 5.0000 | -4.421 |

#### 1-way Test, ChiSquare Approximation

| ChiSquare | DF | Prob>ChiSq |
|---|---|---|
| 27.3103 | 3 | <.0001* |

#### Means Comparisons

##### Comparisons for each pair using Student's t

| Level | - Level | Difference | Lower CL | Upper CL | p-Value |
|---|---|---|---|---|---|
| 24h | 7days | 0.6212222 | 0.511607 | 0.7308374 | <.0001* |
| 48h | 7days | 0.4018889 | 0.292274 | 0.5115041 | <.0001* |
| CTRL | 7days | 0.3406667 | 0.231051 | 0.4502818 | <.0001* |
| 24h | CTRL | 0.2805556 | 0.170940 | 0.3901707 | <.0001* |
| 24h | 48h | 0.2193333 | 0.109718 | 0.3289485 | 0.0003* |
| 48h | CTRL | 0.0612222 | -0.048393 | 0.1708374 | 0.2637 |

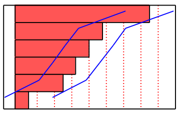

#### Power Details

Test TIME

##### Power

| α | σ | δ | Number | Power |
|---|---|---|---|---|
| 0.0500 | 0.114156 | 0.222774 | 36 | 1.0000 |

Statistical Table 1.
Statistical analysis of data presented in Figure 1, panel B

## Fit Y by X Group

### Oneway Analysis of H3K4m3 By TIME PCR=Tf/Tr

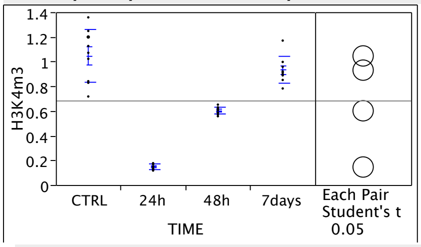


#### Means and Std Deviations

| Level | Number | Mean | Std Dev | Std Err Mean | Lower 95% | Upper 95% |
|---|---|---|---|---|---|---|
| CTRL | 9 | 1.04556 | 0.214169 | 0.07139 | 0.88093 | 1.2102 |
| 24h | 9 | 0.14567 | 0.022130 | 0.00738 | 0.12866 | 0.1627 |
| 48h | 9 | 0.60133 | 0.029576 | 0.00986 | 0.57860 | 0.6241 |
| 7days | 9 | 0.93056 | 0.108550 | 0.03618 | 0.84712 | 1.0140 |

#### Wilcoxon / Kruskal-Wallis Tests (Rank Sums)

| Level | Count | Score Sum | Score Mean | (Mean-Mean0)/Std0 |
|---|---|---|---|---|
| CTRL | 9 | 260.000 | 28.8889 | 3.398 |
| 24h | 9 | 45.000 | 5.0000 | -4.421 |
| 48h | 9 | 126.000 | 14.0000 | -1.461 |
| 7days | 9 | 235.000 | 26.1111 | 2.484 |

#### 1-way Test, ChiSquare Approximation

| ChiSquare | DF | Prob>ChiSq |
|---|---|---|
| 29.8669 | 3 | <.0001* |

#### Means Comparisons

##### Comparisons for each pair using Student's t

| Level | - Level | Difference | Lower CL | Upper CL | p-Value |
|---|---|---|---|---|---|
| CTRL | 24h | 0.8998889 | 0.783255 | 1.016523 | <.0001* |
| 7days | 24h | 0.7848889 | 0.668255 | 0.901523 | <.0001* |
| 48h | 24h | 0.4556667 | 0.339033 | 0.572301 | <.0001* |
| CTRL | 48h | 0.4442222 | 0.327588 | 0.560856 | <.0001* |
| 7days | 48h | 0.3292222 | 0.212588 | 0.445856 | <.0001* |
| CTRL | 7days | 0.1150000 | -0.001634 | 0.231634 | 0.0531 |

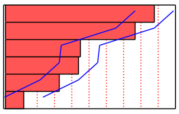

#### Power Details

Test TIME

##### Power

| α | σ | δ | Number | Power |
|---|---|---|---|---|
| 0.0500 | 0.121466 | 0.349323 | 36 | 1.0000 |

### Oneway Analysis of H3K4m2 By TIME PCR=Tf/Tr

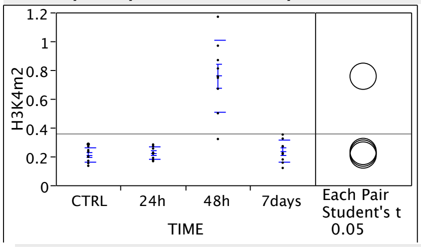


#### Means and Std Deviations

| Level | Number | Mean | Std Dev | Std Err Mean | Lower 95% | Upper 95% |
|---|---|---|---|---|---|---|
| CTRL | 9 | 0.209778 | 0.052002 | 0.01733 | 0.16981 | 0.24975 |
| 24h | 9 | 0.223111 | 0.041205 | 0.01374 | 0.19144 | 0.25478 |
| 48h | 9 | 0.756889 | 0.248699 | 0.08290 | 0.56572 | 0.94806 |
| 7days | 9 | 0.234778 | 0.076684 | 0.02556 | 0.17583 | 0.29372 |

#### Wilcoxon / Kruskal-Wallis Tests (Rank Sums)

| Level | Count | Score Sum | Score Mean | (Mean-Mean0)/Std0 |
|---|---|---|---|---|
| CTRL | 9 | 112.500 | 12.5000 | -1.955 |
| 24h | 9 | 130.000 | 14.4444 | -1.315 |
| 48h | 9 | 286.000 | 31.7778 | 4.348 |
| 7days | 9 | 137.500 | 15.2778 | -1.041 |

#### 1-way Test, ChiSquare Approximation

| ChiSquare | DF | Prob>ChiSq |
|---|---|---|
| 19.3914 | 3 | 0.0002* |

#### Means Comparisons

##### Comparisons for each pair using Student's t

| Level | - Level | Difference | Lower CL | Upper CL | p-Value |
|---|---|---|---|---|---|
| 48h | CTRL | 0.5471111 | 0.418165 | 0.6760576 | <.0001* |
| 48h | 24h | 0.5337778 | 0.404831 | 0.6627242 | <.0001* |
| 48h | 7days | 0.5221111 | 0.393165 | 0.6510576 | <.0001* |
| 7days | CTRL | 0.0250000 | -0.103946 | 0.1539465 | 0.6955 |
| 24h | CTRL | 0.0133333 | -0.115613 | 0.1422798 | 0.8345 |
| 7days | 24h | 0.0116667 | -0.117280 | 0.1406131 | 0.8549 |

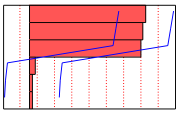

#### Power Details

Test TIME

##### Power

| α | σ | δ | Number | Power |
|---|---|---|---|---|
| 0.0500 | 0.134289 | 0.231542 | 36 | 1.0000 |

Statistical Table 2.
Statistical analysis of data presented in Figure 1, panel C

## Fit Y by X Group

### Oneway Analysis of ctrl Ig By Treat

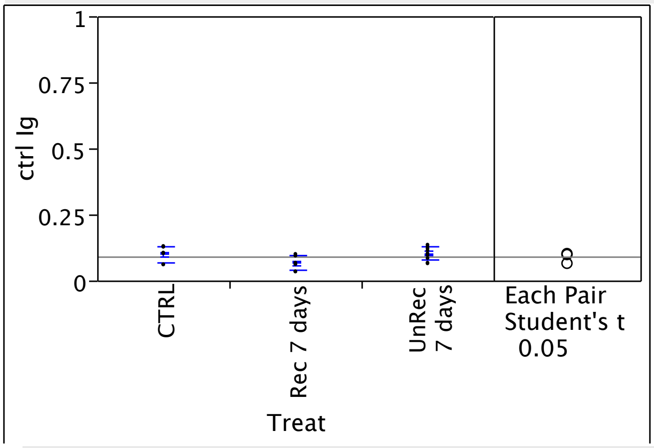


### Means and Std Deviations

| Level | Number | Mean | Std Dev | Std Err Mean | Lower 95% | Upper 95% |
|---|---|---|---|---|---|---|
| CTRL | 9 | 0.098333 | 0.029707 | 0.00990 | 0.07550 | 0.12117 |
| Rec 7 days | 9 | 0.065222 | 0.026971 | 0.00899 | 0.04449 | 0.08595 |
| UnRec 7 days | 9 | 0.102000 | 0.025690 | 0.00856 | 0.08225 | 0.12175 |

### Wilcoxon / Kruskal-Wallis Tests (Rank Sums)

| Level | Count | Score Sum | Score Mean | (Mean-Mean0)/Std0 |
|---|---|---|---|---|
| CTRL | 9 | 145.000 | 16.1111 | 0.952 |
| Rec 7 days | 9 | 74.000 | 8.2222 | -2.651 |
| UnRec 7 days | 9 | 159.000 | 17.6667 | 1.673 |

#### 1-way Test, ChiSquare Approximation

| ChiSquare | DF | Prob>ChiSq |
|---|---|---|
| 7.3397 | 2 | 0.0255* |

### Means Comparisons

#### Comparisons for each pair using Student's t

| Level | - Level | Difference | Lower CL | Upper CL | p-Value |
|---|---|---|---|---|---|
| UnRec 7 days | Rec 7 days | 0.0367778 | 0.010015 | 0.0635404 | 0.0091* |
| CTRL | Rec 7 days | 0.0331111 | 0.006348 | 0.0598738 | 0.0174* |
| UnRec 7 days | CTRL | 0.0036667 | -0.023096 | 0.0304293 | 0.7798 |

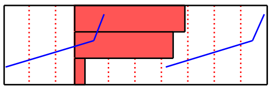

### Power Details

Test Treat

#### Power

| α | σ | δ | Number | Power |
|---|---|---|---|---|
| 0.0500 | 0.027507 | 0.016541 | 27 | 0.7503 |

### Median Test (Number of Points Above Median)

| Level | Count | Score Sum | Score Mean | (Mean-Mean0)/Std0 |
|---|---|---|---|---|
| CTRL | 9 | 6.000 | 0.666667 | 1.336 |
| Rec 7 days | 9 | 1.000 | 0.111111 | -2.673 |
| UnRec 7 days | 9 | 6.000 | 0.666667 | 1.336 |

#### 1-way Test, ChiSquare Approximation

| ChiSquare | DF | Prob>ChiSq |
|---|---|---|
| 7.1429 | 2 | 0.0281* |

## Fit Y by X Group

### Oneway Analysis of H3K9m3 By Treat

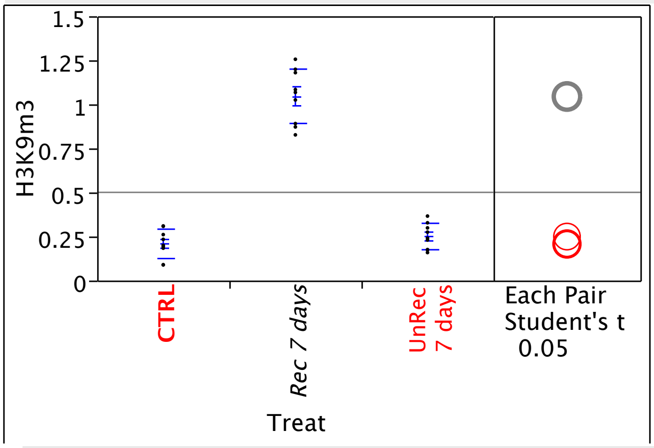


Statistical Table 2.
Statistical analysis of data presented in Figure 1, panel C

### Means and Std Deviations

| Level | Number | Mean | Std Dev | Std Err Mean | Lower 95% | Upper 95% |
|---|---|---|---|---|---|---|
| CTRL | 9 | 0.20767 | 0.081270 | 0.02709 | 0.14520 | 0.2701 |
| Rec 7 days | 9 | 1.04467 | 0.154143 | 0.05138 | 0.92618 | 1.1632 |
| UnRec 7 days | 9 | 0.24978 | 0.072955 | 0.02432 | 0.19370 | 0.3059 |

### Wilcoxon / Kruskal-Wallis Tests (Rank Sums)

| Level | Count | Score Sum | Score Mean | (Mean-Mean0)/Std0 |
|---|---|---|---|---|
| CTRL | 9 | 77.000 | 8.5556 | -2.495 |
| Rec 7 days | 9 | 207.000 | 23.0000 | 4.140 |
| UnRec 7 days | 9 | 94.000 | 10.4444 | -1.620 |

#### 1-way Test, ChiSquare Approximation

| ChiSquare | DF | Prob>ChiSq |
|---|---|---|
| 17.6120 | 2 | 0.0001* |

### Means Comparisons

#### Comparisons for each pair using Student's t

| Level | - Level | Difference | Lower CL | Upper CL | p-Value |
|---|---|---|---|---|---|
| Rec 7 days | CTRL | 0.8370000 | 0.730885 | 0.9431152 | <.0001* |
| Rec 7 days | UnRec 7 days | 0.7948889 | 0.688774 | 0.9010041 | <.0001* |
| UnRec 7 days | CTRL | 0.0421111 | -0.064004 | 0.1482263 | 0.4208 |

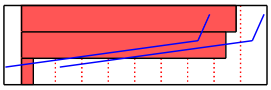

### Power Details

Test Treat

#### Power

| α | σ | δ | Number | Power |
|---|---|---|---|---|
| 0.0500 | 0.109068 | 0.385024 | 27 | 1.0000 |

### Median Test (Number of Points Above Median)

| Level | Count | Score Sum | Score Mean | (Mean-Mean0)/Std0 |
|---|---|---|---|---|
| CTRL | 9 | 2.000 | 0.22222 | -1.871 |
| Rec 7 days | 9 | 9.000 | 1.00000 | 3.742 |
| UnRec 7 days | 9 | 2.000 | 0.22222 | -1.871 |

#### 1-way Test, ChiSquare Approximation

| ChiSquare | DF | Prob>ChiSq |
|---|---|---|
| 14.0000 | 2 | 0.0009* |

## Fit Y by X Group

### Oneway Analysis of H3K9m2 By Treat

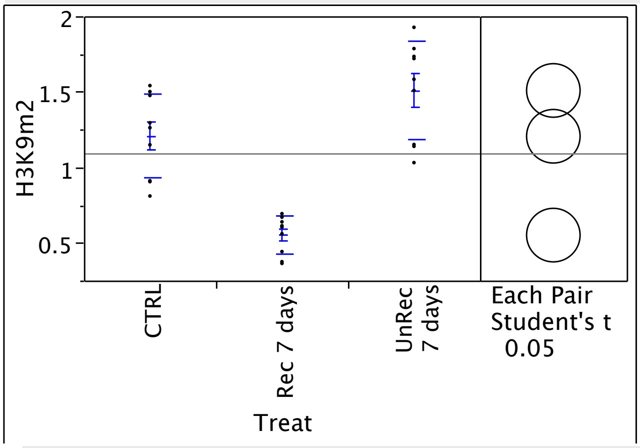


Statistical Table 2.
Statistical analysis of data presented in Figure 1, panel C

### Means and Std Deviations

| Level | Number | Mean | Std Dev | Std Err Mean | Lower 95% | Upper 95% |
|---|---|---|---|---|---|---|
| CTRL | 9 | 1.20556 | 0.278589 | 0.09286 | 0.9914 | 1.4197 |
| Rec 7 days | 9 | 0.55111 | 0.125812 | 0.04194 | 0.4544 | 0.6478 |
| UnRec 7 days | 9 | 1.50889 | 0.325120 | 0.10837 | 1.2590 | 1.7588 |

### Wilcoxon / Kruskal-Wallis Tests (Rank Sums)

| Level | Count | Score Sum | Score Mean | (Mean-Mean0)/Std0 |
|---|---|---|---|---|
| CTRL | 9 | 144.000 | 16.0000 | 0.900 |
| Rec 7 days | 9 | 45.000 | 5.0000 | -4.140 |
| UnRec 7 days | 9 | 189.000 | 21.0000 | 3.215 |

#### 1-way Test, ChiSquare Approximation

| ChiSquare | DF | Prob>ChiSq |
|---|---|---|
| 19.1429 | 2 | <.0001* |

### Means Comparisons

#### Comparisons for each pair using Student's t

| Level | - Level | Difference | Lower CL | Upper CL | p-Value |
|---|---|---|---|---|---|
| UnRec 7 days | Rec 7 days | 0.9577778 | 0.7071065 | 1.208449 | <.0001* |
| CTRL | Rec 7 days | 0.6544444 | 0.4037732 | 0.905116 | <.0001* |
| UnRec 7 days | CTRL | 0.3033333 | 0.0526621 | 0.554005 | 0.0198* |

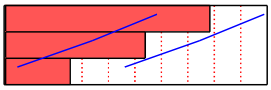

### Power Details

Test Treat

#### Power

| α | σ | δ | Number | Power |
|---|---|---|---|---|
| 0.0500 | 0.257645 | 0.399673 | 27 | 1.0000 |

### Median Test (Number of Points Above Median)

| Level | Count | Score Sum | Score Mean | (Mean-Mean0)/Std0 |
|---|---|---|---|---|
| CTRL | 9 | 6.000 | 0.666667 | 1.336 |
| Rec 7 days | 9 | 0.000 | 0.000000 | -3.474 |
| UnRec 7 days | 9 | 7.000 | 0.777778 | 2.138 |

#### 1-way Test, ChiSquare Approximation

| ChiSquare | DF | Prob>ChiSq |
|---|---|---|
| 12.2857 | 2 | 0.0021* |

## Fit Y by X Group

### Oneway Analysis of H3K4m3 By Treat

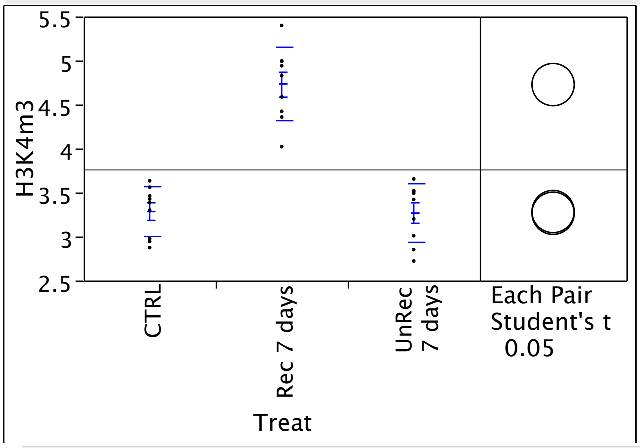


Statistical Table 2.
Statistical analysis of data presented in Figure 1, panel C

### Means and Std Deviations

| Level | Number | Mean | Std Dev | Std Err Mean | Lower 95% | Upper 95% |
|---|---|---|---|---|---|---|
| CTRL | 9 | 3.28433 | 0.282587 | 0.09420 | 3.0671 | 3.5015 |
| Rec 7 days | 9 | 4.72667 | 0.417515 | 0.13917 | 4.4057 | 5.0476 |
| UnRec 7 days | 9 | 3.26433 | 0.333211 | 0.11107 | 3.0082 | 3.5205 |

### Wilcoxon / Kruskal-Wallis Tests (Rank Sums)

| Level | Count | Score Sum | Score Mean | (Mean-Mean0)/Std0 |
|---|---|---|---|---|
| CTRL | 9 | 85.000 | 9.4444 | -2.083 |
| Rec 7 days | 9 | 207.000 | 23.0000 | 4.140 |
| UnRec 7 days | 9 | 86.000 | 9.5556 | -2.032 |

#### 1-way Test, ChiSquare Approximation

| ChiSquare | DF | Prob>ChiSq |
|---|---|---|
| 17.3580 | 2 | 0.0002* |

### Means Comparisons

#### Comparisons for each pair using Student's t

| Level | - Level | Difference | Lower CL | Upper CL | p-Value |
|---|---|---|---|---|---|
| Rec 7 days | UnRec 7 days | 1.462333 | 1.12287 | 1.801794 | <.0001* |
| Rec 7 days | CTRL | 1.442333 | 1.10287 | 1.781794 | <.0001* |
| CTRL | UnRec 7 days | 0.020000 | -0.31946 | 0.359460 | 0.9042 |

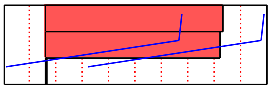

### Power Details

Test Treat

#### Power

| $\alpha$ | $\sigma$ | $\delta$ | Number | Power |
|---|---|---|---|---|
| 0.0500 | 0.348905 | 0.684685 | 27 | 1.0000 |

### Median Test (Number of Points Above Median)

| Level | Count | Score Sum | Score Mean | (Mean-Mean0)/Std0 |
|---|---|---|---|---|
| CTRL | 9 | 2.000 | 0.22222 | -1.871 |
| Rec 7 days | 9 | 9.000 | 1.00000 | 3.742 |
| UnRec 7 days | 9 | 2.000 | 0.22222 | -1.871 |

#### 1-way Test, ChiSquare Approximation

| ChiSquare | DF | Prob>ChiSq |
|---|---|---|
| 14.0000 | 2 | 0.0009* |

## Fit Y by X Group

### Oneway Analysis of H3K4m2 By Treat

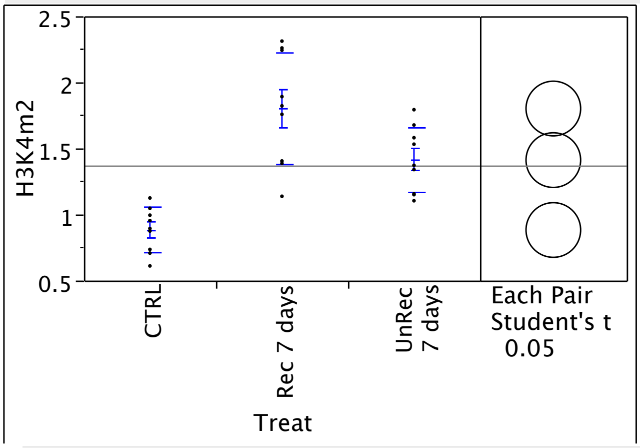


Statistical Table 2.
Statistical analysis of data presented in Figure 1, panel C

### Means and Std Deviations

| Level | Number | Mean | Std Dev | Std Err Mean | Lower 95% | Upper 95% |
|---|---|---|---|---|---|---|
| CTRL | 9 | 0.88278 | 0.169354 | 0.05645 | 0.7526 | 1.0130 |
| Rec 7 days | 9 | 1.80189 | 0.425075 | 0.14169 | 1.4751 | 2.1286 |
| UnRec 7 days | 9 | 1.41167 | 0.248539 | 0.08285 | 1.2206 | 1.6027 |

### Wilcoxon / Kruskal-Wallis Tests (Rank Sums)

| Level | Count | Score Sum | Score Mean | (Mean-Mean0)/Std0 |
|---|---|---|---|---|
| CTRL | 9 | 46.000 | 5.1111 | -4.089 |
| Rec 7 days | 9 | 190.000 | 21.1111 | 3.266 |
| UnRec 7 days | 9 | 142.000 | 15.7778 | 0.797 |

#### 1-way Test, ChiSquare Approximation

| ChiSquare | DF | Prob>ChiSq |
|---|---|---|
| 18.9630 | 2 | <.0001* |

### Means Comparisons

#### Comparisons for each pair using Student's t

| Level | - Level | Difference | Lower CL | Upper CL | p-Value |
|---|---|---|---|---|---|
| Rec 7 days | CTRL | 0.9191111 | 0.6266154 | 1.211607 | <.0001* |
| UnRec 7 days | CTRL | 0.5288889 | 0.2363932 | 0.821385 | 0.0010* |
| Rec 7 days | UnRec 7 days | 0.3902222 | 0.0977265 | 0.682718 | 0.0111* |

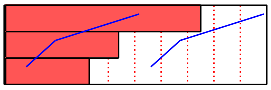

### Power Details

Test Treat

#### Power

| α | σ | δ | Number | Power |
|---|---|---|---|---|
| 0.0500 | 0.300634 | 0.376646 | 27 | 0.9999 |

### Median Test (Number of Points Above Median)

| Level | Count | Score Sum | Score Mean | (Mean-Mean0)/Std0 |
|---|---|---|---|---|
| CTRL | 9 | 0.000 | 0.000000 | -3.474 |
| Rec 7 days | 9 | 8.000 | 0.888889 | 2.940 |
| UnRec 7 days | 9 | 5.000 | 0.555556 | 0.535 |

#### 1-way Test, ChiSquare Approximation

| ChiSquare | DF | Prob>ChiSq |
|---|---|---|
| 14.0000 | 2 | 0.0009* |

Statistical Table 3.
Statistical analysis of data presented in Figure 2, panel B

## Fit Y by X Group

### Oneway Analysis of Ig CTRL By TREAT PCR=a/c

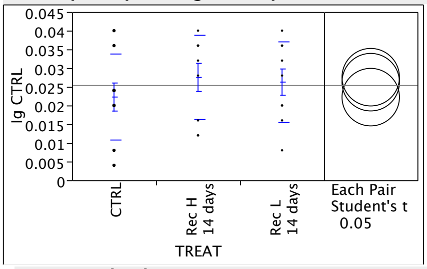


#### Means and Std Deviations

| Level | Number | Mean | Std Dev | Std Err Mean | Lower 95% | Upper 95% |
|---|---|---|---|---|---|---|
| CTRL | 9 | 0.022222 | 0.011508 | 0.00384 | 0.01338 | 0.03107 |
| Rec H 14 days | 9 | 0.027556 | 0.011215 | 0.00374 | 0.01893 | 0.03618 |
| Rec L 14 days | 9 | 0.026222 | 0.010791 | 0.00360 | 0.01793 | 0.03452 |

#### Wilcoxon / Kruskal-Wallis Tests (Rank Sums)

| Level | Count | Score Sum | Score Mean | (Mean-Mean0)/Std0 |
|---|---|---|---|---|
| CTRL | 9 | 109.000 | 12.1111 | -0.856 |
| Rec H 14 days | 9 | 139.000 | 15.4444 | 0.649 |
| Rec L 14 days | 9 | 130.000 | 14.4444 | 0.182 |

#### 1-way Test, ChiSquare Approximation

| ChiSquare | DF | Prob>ChiSq |
|---|---|---|
| 0.8510 | 2 | 0.6534 |

#### Means Comparisons

##### Comparisons for each pair using Student's t

| Level | - Level | Difference | Lower CL | Upper CL | p-Value |
|---|---|---|---|---|---|
| Rec H 14 days | CTRL | 0.0053333 | -0.005540 | 0.0162062 | 0.3215 |
| Rec L 14 days | CTRL | 0.0040000 | -0.006873 | 0.0148729 | 0.4551 |
| Rec H 14 days | Rec L 14 days | 0.0013333 | -0.009540 | 0.0122062 | 0.8024 |

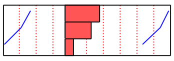

### Oneway Analysis of H3K9m3 By TREAT PCR=a/c

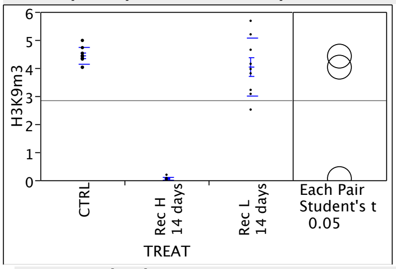


#### Means and Std Deviations

| Level | Number | Mean | Std Dev | Std Err Mean | Lower 95% | Upper 95% |
|---|---|---|---|---|---|---|
| CTRL | 9 | 4.42367 | 0.30581 | 0.10194 | 4.1886 | 4.6587 |
| Rec H 14 days | 9 | 0.05756 | 0.05778 | 0.01926 | 0.0131 | 0.1020 |
| Rec L 14 days | 9 | 4.03044 | 1.02458 | 0.34153 | 3.2429 | 4.8180 |

#### Wilcoxon / Kruskal-Wallis Tests (Rank Sums)

| Level | Count | Score Sum | Score Mean | (Mean-Mean0)/Std0 |
|---|---|---|---|---|
| CTRL | 9 | 180.000 | 20.0000 | 2.752 |
| Rec H 14 days | 9 | 45.000 | 5.0000 | -4.140 |
| Rec L 14 days | 9 | 153.000 | 17.0000 | 1.363 |

#### 1-way Test, ChiSquare Approximation

| ChiSquare | DF | Prob>ChiSq |
|---|---|---|
| 18.0000 | 2 | 0.0001* |

#### Means Comparisons

##### Comparisons for each pair using Student's t

| Level | - Level | Difference | Lower CL | Upper CL | p-Value |
|---|---|---|---|---|---|
| CTRL | Rec H 14 days | 4.366111 | 3.76462 | 4.967603 | <.0001* |
| Rec L 14 days | Rec H 14 days | 3.972889 | 3.37140 | 4.574381 | <.0001* |
| CTRL | Rec L 14 days | 0.393222 | -0.20827 | 0.994715 | 0.1898 |

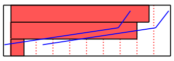

### Oneway Analysis of H3K9m2 By TREAT PCR=a/c

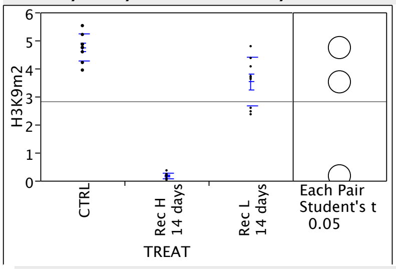


#### Means and Std Deviations

| Level | Number | Mean | Std Dev | Std Err Mean | Lower 95% | Upper 95% |
|---|---|---|---|---|---|---|
| CTRL | 9 | 4.74556 | 0.475057 | 0.15835 | 4.3804 | 5.1107 |
| Rec H 14 days | 9 | 0.17433 | 0.103056 | 0.03435 | 0.0951 | 0.2535 |
| Rec L 14 days | 9 | 3.52411 | 0.867069 | 0.28902 | 2.8576 | 4.1906 |

#### Wilcoxon / Kruskal-Wallis Tests (Rank Sums)

| Level | Count | Score Sum | Score Mean | (Mean-Mean0)/Std0 |
|---|---|---|---|---|
| CTRL | 9 | 199.000 | 22.1111 | 3.729 |
| Rec H 14 days | 9 | 45.000 | 5.0000 | -4.140 |
| Rec L 14 days | 9 | 134.000 | 14.8889 | 0.386 |

#### 1-way Test, ChiSquare Approximation

| ChiSquare | DF | Prob>ChiSq |
|---|---|---|
| 21.0829 | 2 | <.0001* |

#### Means Comparisons

##### Comparisons for each pair using Student's t

| Level | - Level | Difference | Lower CL | Upper CL | p-Value |
|---|---|---|---|---|---|
| CTRL | Rec H 14 days | 4.571222 | 4.012850 | 5.129594 | <.0001* |
| Rec L 14 days | Rec H 14 days | 3.349778 | 2.791406 | 3.908150 | <.0001* |
| CTRL | Rec L 14 days | 1.221444 | 0.663072 | 1.779817 | 0.0001* |

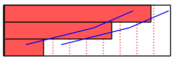

Statistical Table 3.
Statistical analysis of data presented in Figure 2, panel B

## Fit Y by X Group

### Oneway Analysis of H3K4m3 By TREAT PCR=a/c

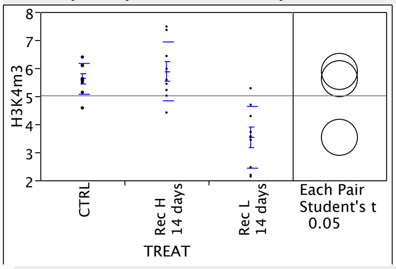


#### Means and Std Deviations

| Level | Number | Mean | Std Dev | Std Err Mean | Lower 95% | Upper 95% |
|---|---|---|---|---|---|---|
| CTRL | 9 | 5.62322 | 0.54547 | 0.18182 | 5.2039 | 6.0425 |
| Rec H 14 days | 9 | 5.88267 | 1.04094 | 0.34698 | 5.0825 | 6.6828 |
| Rec L 14 days | 9 | 3.53300 | 1.11258 | 0.37086 | 2.6778 | 4.3882 |

#### Wilcoxon / Kruskal-Wallis Tests (Rank Sums)

| Level | Count | Score Sum | Score Mean | (Mean-Mean0)/Std0 |
|---|---|---|---|---|
| CTRL | 9 | 162.000 | 18.0000 | 1.826 |
| Rec H 14 days | 9 | 164.000 | 18.2222 | 1.929 |
| Rec L 14 days | 9 | 52.000 | 5.7778 | -3.780 |

##### 1-way Test, ChiSquare Approximation

| ChiSquare | DF | Prob>ChiSq |
|---|---|---|
| 14.4903 | 2 | 0.0007* |

#### Means Comparisons

##### Comparisons for each pair using Student's t

| Level | - Level | Difference | Lower CL | Upper CL | p-Value |
|---|---|---|---|---|---|
| Rec H 14 days | Rec L 14 days | 2.349667 | 1.44062 | 3.258710 | <.0001* |
| CTRL | Rec L 14 days | 2.090222 | 1.18118 | 2.999265 | <.0001* |
| Rec H 14 days | CTRL | 0.259444 | -0.64960 | 1.168488 | 0.5613 |

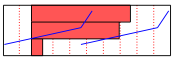

### Oneway Analysis of H3K4m2 By TREAT PCR=a/c

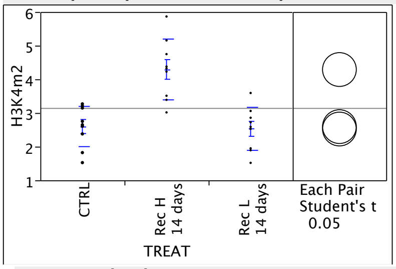


#### Means and Std Deviations

| Level | Number | Mean | Std Dev | Std Err Mean | Lower 95% | Upper 95% |
|---|---|---|---|---|---|---|
| CTRL | 9 | 2.59444 | 0.607542 | 0.20251 | 2.1274 | 3.0614 |
| Rec H 14 days | 9 | 4.28989 | 0.900818 | 0.30027 | 3.5975 | 4.9823 |
| Rec L 14 days | 9 | 2.52267 | 0.644924 | 0.21497 | 2.0269 | 3.0184 |

#### Wilcoxon / Kruskal-Wallis Tests (Rank Sums)

| Level | Count | Score Sum | Score Mean | (Mean-Mean0)/Std0 |
|---|---|---|---|---|
| CTRL | 9 | 93.000 | 10.3333 | -1.672 |
| Rec H 14 days | 9 | 200.000 | 22.2222 | 3.780 |
| Rec L 14 days | 9 | 85.000 | 9.4444 | -2.083 |

##### 1-way Test, ChiSquare Approximation

| ChiSquare | DF | Prob>ChiSq |
|---|---|---|
| 14.5432 | 2 | 0.0007* |

#### Means Comparisons

##### Comparisons for each pair using Student's t

| Level | - Level | Difference | Lower CL | Upper CL | p-Value |
|---|---|---|---|---|---|
| Rec H 14 days | Rec L 14 days | 1.767222 | 1.05747 | 2.476975 | <.0001* |
| Rec H 14 days | CTRL | 1.695444 | 0.98569 | 2.405197 | <.0001* |
| CTRL | Rec L 14 days | 0.071778 | -0.63797 | 0.781531 | 0.8364 |

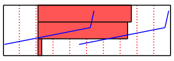

## Fit Y by X Group

### Oneway Analysis of Ig CTRL By TREAT PCR=p/q

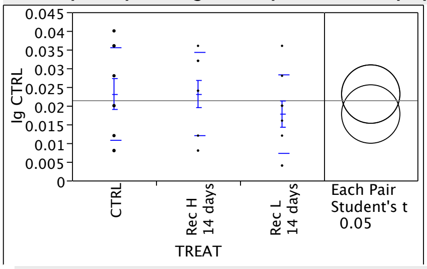


#### Means and Std Deviations

| Level | Number | Mean | Std Dev | Std Err Mean | Lower 95% | Upper 95% |
|---|---|---|---|---|---|---|
| CTRL | 9 | 0.023111 | 0.012454 | 0.00415 | 0.01354 | 0.03268 |
| Rec H 14 days | 9 | 0.023111 | 0.011096 | 0.00370 | 0.01458 | 0.03164 |
| Rec L 14 days | 9 | 0.017778 | 0.010414 | 0.00347 | 0.00977 | 0.02578 |

#### Wilcoxon / Kruskal-Wallis Tests (Rank Sums)

| Level | Count | Score Sum | Score Mean | (Mean-Mean0)/Std0 |
|---|---|---|---|---|
| CTRL | 9 | 137.500 | 15.2778 | 0.570 |
| Rec H 14 days | 9 | 137.500 | 15.2778 | 0.570 |
| Rec L 14 days | 9 | 103.000 | 11.4444 | -1.166 |

##### 1-way Test, ChiSquare Approximation

| ChiSquare | DF | Prob>ChiSq |
|---|---|---|
| 1.4216 | 2 | 0.4913 |

#### Means Comparisons

##### Comparisons for each pair using Student's t

| Level | - Level | Difference | Lower CL | Upper CL | p-Value |
|---|---|---|---|---|---|
| CTRL | Rec L 14 days | 0.0053333 | -0.005712 | 0.0163789 | 0.3289 |
| Rec H 14 days | Rec L 14 days | 0.0053333 | -0.005712 | 0.0163789 | 0.3289 |
| Rec H 14 days | CTRL | 0.0000000 | -0.011046 | 0.0110456 | 1.0000 |

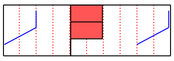

Statistical Table 3.
Statistical analysis of data presented in Figure 2, panel B

## Fit Y by X Group

### Oneway Analysis of H3K9m3 By TREAT PCR=p/q

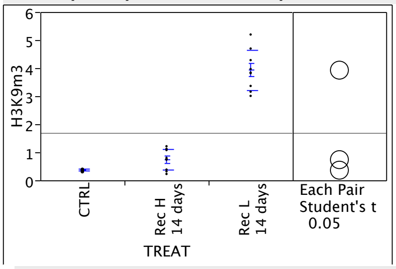


#### Means and Std Deviations

| Level | Number | Mean | Std Dev | Std Err Mean | Lower 95% | Upper 95% |
|---|---|---|---|---|---|---|
| CTRL | 9 | 0.36167 | 0.033619 | 0.01121 | 0.3358 | 0.3875 |
| Rec H 14 days | 9 | 0.73711 | 0.363637 | 0.12121 | 0.4576 | 1.0166 |
| Rec L 14 days | 9 | 3.92733 | 0.716216 | 0.23874 | 3.3768 | 4.4779 |

#### Wilcoxon / Kruskal-Wallis Tests (Rank Sums)

| Level | Count | Score Sum | Score Mean | (Mean-Mean0)/Std0 |
|---|---|---|---|---|
| CTRL | 9 | 64.000 | 7.1111 | -3.163 |
| Rec H 14 days | 9 | 107.000 | 11.8889 | -0.952 |
| Rec L 14 days | 9 | 207.000 | 23.0000 | 4.140 |

#### 1-way Test, ChiSquare Approximation

| ChiSquare | DF | Prob>ChiSq |
|---|---|---|
| 18.9877 | 2 | <.0001* |

#### Means Comparisons

##### Comparisons for each pair using Student's t

| Level | - Level | Difference | Lower CL | Upper CL | p-Value |
|---|---|---|---|---|---|
| Rec L 14 days | CTRL | 3.565667 | 3.11407 | 4.017260 | <.0001* |
| Rec L 14 days | Rec H 14 days | 3.190222 | 2.73863 | 3.641816 | <.0001* |
| Rec H 14 days | CTRL | 0.375444 | -0.07615 | 0.827038 | 0.0991 |

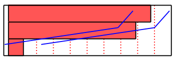

### Oneway Analysis of H3K9m2 By TREAT PCR=p/q

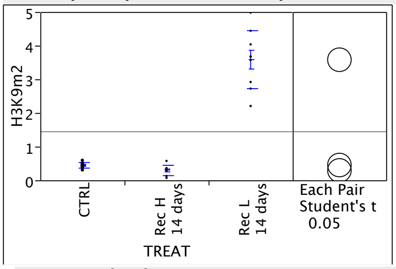


#### Means and Std Deviations

| Level | Number | Mean | Std Dev | Std Err Mean | Lower 95% | Upper 95% |
|---|---|---|---|---|---|---|
| CTRL | 9 | 0.45378 | 0.084269 | 0.02809 | 0.3890 | 0.5186 |
| Rec H 14 days | 9 | 0.29278 | 0.142598 | 0.04753 | 0.1832 | 0.4024 |
| Rec L 14 days | 9 | 3.58133 | 0.865235 | 0.28841 | 2.9163 | 4.2464 |

#### Wilcoxon / Kruskal-Wallis Tests (Rank Sums)

| Level | Count | Score Sum | Score Mean | (Mean-Mean0)/Std0 |
|---|---|---|---|---|
| CTRL | 9 | 116.000 | 12.8889 | -0.489 |
| Rec H 14 days | 9 | 55.000 | 6.1111 | -3.627 |
| Rec L 14 days | 9 | 207.000 | 23.0000 | 4.142 |

#### 1-way Test, ChiSquare Approximation

| ChiSquare | DF | Prob>ChiSq |
|---|---|---|
| 20.6511 | 2 | <.0001* |

#### Means Comparisons

##### Comparisons for each pair using Student's t

| Level | - Level | Difference | Lower CL | Upper CL | p-Value |
|---|---|---|---|---|---|
| Rec L 14 days | Rec H 14 days | 3.288556 | 2.79371 | 3.783403 | <.0001* |
| Rec L 14 days | CTRL | 3.127556 | 2.63271 | 3.622403 | <.0001* |
| CTRL | Rec H 14 days | 0.161000 | -0.33385 | 0.655847 | 0.5083 |

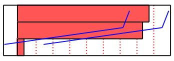

### Oneway Analysis of H3K4m3 By TREAT PCR=p/q

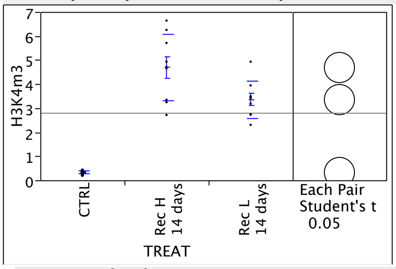


#### Means and Std Deviations

| Level | Number | Mean | Std Dev | Std Err Mean | Lower 95% | Upper 95% |
|---|---|---|---|---|---|---|
| CTRL | 9 | 0.32333 | 0.06231 | 0.02077 | 0.2754 | 0.3712 |
| Rec H 14 days | 9 | 4.68933 | 1.37593 | 0.45864 | 3.6317 | 5.7470 |
| Rec L 14 days | 9 | 3.35467 | 0.77162 | 0.25721 | 2.7615 | 3.9478 |

#### Wilcoxon / Kruskal-Wallis Tests (Rank Sums)

| Level | Count | Score Sum | Score Mean | (Mean-Mean0)/Std0 |
|---|---|---|---|---|
| CTRL | 9 | 45.000 | 5.0000 | -4.140 |
| Rec H 14 days | 9 | 186.000 | 20.6667 | 3.060 |
| Rec L 14 days | 9 | 147.000 | 16.3333 | 1.054 |

#### 1-way Test, ChiSquare Approximation

| ChiSquare | DF | Prob>ChiSq |
|---|---|---|
| 18.6984 | 2 | <.0001* |

#### Means Comparisons

##### Comparisons for each pair using Student's t

| Level | - Level | Difference | Lower CL | Upper CL | p-Value |
|---|---|---|---|---|---|
| Rec H 14 days | CTRL | 4.366000 | 3.479177 | 5.252823 | <.0001* |
| Rec L 14 days | CTRL | 3.031333 | 2.144510 | 3.918156 | <.0001* |
| Rec H 14 days | Rec L 14 days | 1.334667 | 0.447844 | 2.221490 | 0.0048* |

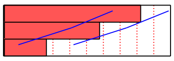

Statistical Table 3.
Statistical analysis of data presented in Figure 2, panel B

## Fit Y by X Group

### Oneway Analysis of H3K4m2 By TREAT PCR=p/q

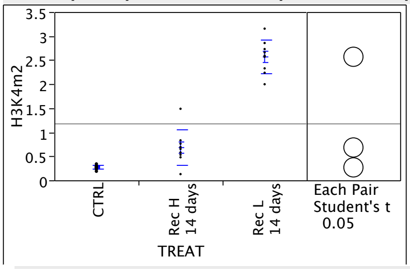


#### Means and Std Deviations

| Level | Number | Mean | Std Dev | Std Err Mean | Lower 95% | Upper 95% |
|---|---|---|---|---|---|---|
| CTRL | 9 | 0.26878 | 0.044192 | 0.01473 | 0.2348 | 0.3027 |
| Rec H 14 days | 9 | 0.68311 | 0.363880 | 0.12129 | 0.4034 | 0.9628 |
| Rec L 14 days | 9 | 2.56911 | 0.345104 | 0.11503 | 2.3038 | 2.8344 |

#### Wilcoxon / Kruskal-Wallis Tests (Rank Sums)

| Level | Count | Score Sum | Score Mean | (Mean-Mean0)/Std0 |
|---|---|---|---|---|
| CTRL | 9 | 54.000 | 6.0000 | -3.678 |
| Rec H 14 days | 9 | 117.000 | 13.0000 | -0.437 |
| Rec L 14 days | 9 | 207.000 | 23.0000 | 4.141 |

#### 1-way Test, ChiSquare Approximation

| ChiSquare | DF | Prob>ChiSq |
|---|---|---|
| 20.8635 | 2 | <.0001* |

#### Means Comparisons

##### Comparisons for each pair using Student's t

| Level | - Level | Difference | Lower CL | Upper CL | p-Value |
|---|---|---|---|---|---|
| Rec L 14 days | CTRL | 2.300333 | 2.017536 | 2.583131 | <.0001* |
| Rec L 14 days | Rec H 14 days | 1.886000 | 1.603203 | 2.168797 | <.0001* |
| Rec H 14 days | CTRL | 0.414333 | 0.131536 | 0.697131 | 0.0059* |

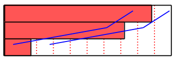

## Fit Y by X Group

### Oneway Analysis of Ig CTRL By TREAT PCR=r/h

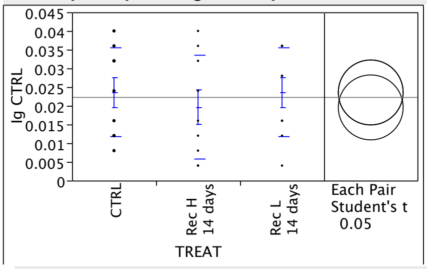


#### Means and Std Deviations

| Level | Number | Mean | Std Dev | Std Err Mean | Lower 95% | Upper 95% |
|---|---|---|---|---|---|---|
| CTRL | 9 | 0.023556 | 0.011907 | 0.00397 | 0.01440 | 0.03271 |
| Rec H 14 days | 9 | 0.019556 | 0.013920 | 0.00464 | 0.00886 | 0.03026 |
| Rec L 14 days | 9 | 0.023556 | 0.011907 | 0.00397 | 0.01440 | 0.03271 |

#### Wilcoxon / Kruskal-Wallis Tests (Rank Sums)

| Level | Count | Score Sum | Score Mean | (Mean-Mean0)/Std0 |
|---|---|---|---|---|
| CTRL | 9 | 133.000 | 14.7778 | 0.337 |
| Rec H 14 days | 9 | 110.500 | 12.2778 | -0.778 |
| Rec L 14 days | 9 | 134.500 | 14.9444 | 0.415 |

#### 1-way Test, ChiSquare Approximation

| ChiSquare | DF | Prob>ChiSq |
|---|---|---|
| 0.6478 | 2 | 0.7233 |

#### Means Comparisons

##### Comparisons for each pair using Student's t

| Level | - Level | Difference | Lower CL | Upper CL | p-Value |
|---|---|---|---|---|---|
| CTRL | Rec H 14 days | 0.0040000 | -0.008272 | 0.0162725 | 0.5076 |
| Rec L 14 days | Rec H 14 days | 0.0040000 | -0.008272 | 0.0162725 | 0.5076 |
| CTRL | Rec L 14 days | 3.469e-18 | -0.012272 | 0.0122725 | 1.0000 |

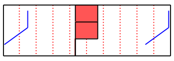

### Oneway Analysis of H3K9m3 By TREAT PCR=r/h

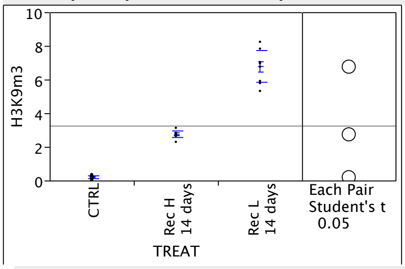


#### Means and Std Deviations

| Level | Number | Mean | Std Dev | Std Err Mean | Lower 95% | Upper 95% |
|---|---|---|---|---|---|---|
| CTRL | 9 | 0.19867 | 0.105235 | 0.03508 | 0.1178 | 0.2796 |
| Rec H 14 days | 9 | 2.74400 | 0.216147 | 0.07205 | 2.5779 | 2.9101 |
| Rec L 14 days | 9 | 6.76411 | 0.952182 | 0.31739 | 6.0322 | 7.4960 |

#### Wilcoxon / Kruskal-Wallis Tests (Rank Sums)

| Level | Count | Score Sum | Score Mean | (Mean-Mean0)/Std0 |
|---|---|---|---|---|
| CTRL | 9 | 45.000 | 5.0000 | -4.140 |
| Rec H 14 days | 9 | 126.000 | 14.0000 | 0.000 |
| Rec L 14 days | 9 | 207.000 | 23.0000 | 4.140 |

#### 1-way Test, ChiSquare Approximation

| ChiSquare | DF | Prob>ChiSq |
|---|---|---|
| 23.1429 | 2 | <.0001* |

#### Means Comparisons

##### Comparisons for each pair using Student's t

| Level | - Level | Difference | Lower CL | Upper CL | p-Value |
|---|---|---|---|---|---|
| Rec L 14 days | CTRL | 6.565444 | 6.013799 | 7.117090 | <.0001* |
| Rec L 14 days | Rec H 14 days | 4.020111 | 3.468466 | 4.571757 | <.0001* |
| Rec H 14 days | CTRL | 2.545333 | 1.993688 | 3.096979 | <.0001* |

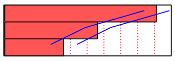

Statistical Table 3.
Statistical analysis of data presented in Figure 2, panel B

## Fit Y by X Group

### Oneway Analysis of H3K9m2 By TREAT PCR=r/h

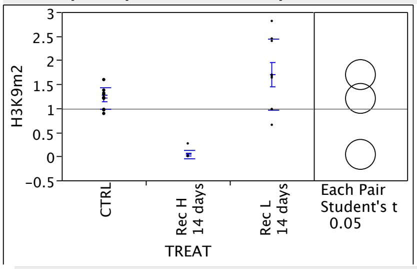


#### Means and Std Deviations

| Level | Number | Mean | Std Dev | Std Err Mean | Lower 95% | Upper 95% |
|---|---|---|---|---|---|---|
| CTRL | 9 | 1.20378 | 0.226046 | 0.07535 | 1.030 | 1.3775 |
| Rec H 14 days | 9 | 0.03956 | 0.085766 | 0.02859 | −0.026 | 0.1055 |
| Rec L 14 days | 9 | 1.69833 | 0.743538 | 0.24785 | 1.127 | 2.2699 |

#### Wilcoxon / Kruskal-Wallis Tests (Rank Sums)

| Level | Count | Score Sum | Score Mean | (Mean-Mean0)/Std0 |
|---|---|---|---|---|
| CTRL | 9 | 147.000 | 16.3333 | 1.054 |
| Rec H 14 days | 9 | 45.000 | 5.0000 | −4.140 |
| Rec L 14 days | 9 | 186.000 | 20.6667 | 3.060 |

#### 1-way Test, ChiSquare Approximation

| ChiSquare | DF | Prob>ChiSq |
|---|---|---|
| 18.6984 | 2 | <.0001* |

#### Means Comparisons

##### Comparisons for each pair using Student's t

| Level | - Level | Difference | Lower CL | Upper CL | p-Value |
|---|---|---|---|---|---|
| Rec L 14 days | Rec H 14 days | 1.658778 | 1.219591 | 2.097965 | <.0001* |
| CTRL | Rec H 14 days | 1.164222 | 0.725035 | 1.603409 | <.0001* |
| Rec L 14 days | CTRL | 0.494556 | 0.055369 | 0.933742 | 0.0289* |

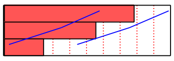

### Oneway Analysis of H3K4m3 By TREAT PCR=r/h

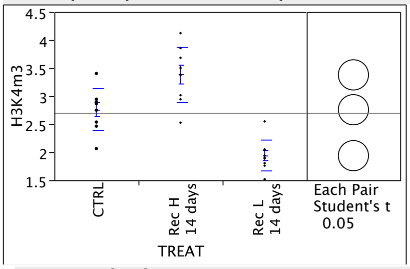


#### Means and Std Deviations

| Level | Number | Mean | Std Dev | Std Err Mean | Lower 95% | Upper 95% |
|---|---|---|---|---|---|---|
| CTRL | 9 | 2.75789 | 0.373277 | 0.12443 | 2.4710 | 3.0448 |
| Rec H 14 days | 9 | 3.37889 | 0.491986 | 0.16400 | 3.0007 | 3.7571 |
| Rec L 14 days | 9 | 1.93900 | 0.279319 | 0.09311 | 1.7243 | 2.1537 |

#### Wilcoxon / Kruskal-Wallis Tests (Rank Sums)

| Level | Count | Score Sum | Score Mean | (Mean-Mean0)/Std0 |
|---|---|---|---|---|
| CTRL | 9 | 135.000 | 15.0000 | 0.437 |
| Rec H 14 days | 9 | 194.000 | 21.5556 | 3.472 |
| Rec L 14 days | 9 | 49.000 | 5.4444 | −3.935 |

#### 1-way Test, ChiSquare Approximation

| ChiSquare | DF | Prob>ChiSq |
|---|---|---|
| 18.7549 | 2 | <.0001* |

#### Means Comparisons

##### Comparisons for each pair using Student's t

| Level | - Level | Difference | Lower CL | Upper CL | p-Value |
|---|---|---|---|---|---|
| Rec H 14 days | Rec L 14 days | 1.439889 | 1.059157 | 1.820621 | <.0001* |
| CTRL | Rec L 14 days | 0.818889 | 0.438157 | 1.199621 | 0.0002* |
| Rec H 14 days | CTRL | 0.621000 | 0.240268 | 1.001732 | 0.0026* |

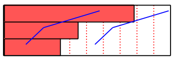

### Oneway Analysis of H3K4m2 By TREAT PCR=r/h

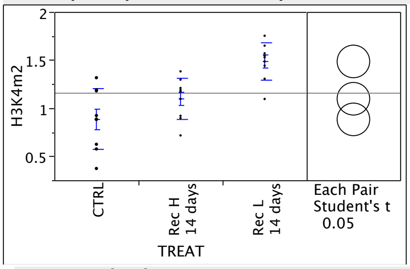


#### Means and Std Deviations

| Level | Number | Mean | Std Dev | Std Err Mean | Lower 95% | Upper 95% |
|---|---|---|---|---|---|---|
| CTRL | 9 | 0.88289 | 0.315166 | 0.10506 | 0.6406 | 1.1251 |
| Rec H 14 days | 9 | 1.09733 | 0.213400 | 0.07113 | 0.9333 | 1.2614 |
| Rec L 14 days | 9 | 1.48522 | 0.192653 | 0.06422 | 1.3371 | 1.6333 |

#### Wilcoxon / Kruskal-Wallis Tests (Rank Sums)

| Level | Count | Score Sum | Score Mean | (Mean-Mean0)/Std0 |
|---|---|---|---|---|
| CTRL | 9 | 73.000 | 8.1111 | −2.701 |
| Rec H 14 days | 9 | 109.000 | 12.1111 | −0.849 |
| Rec L 14 days | 9 | 196.000 | 21.7778 | 3.575 |

#### 1-way Test, ChiSquare Approximation

| ChiSquare | DF | Prob>ChiSq |
|---|---|---|
| 14.1101 | 2 | 0.0009* |

#### Means Comparisons

##### Comparisons for each pair using Student's t

| Level | - Level | Difference | Lower CL | Upper CL | p-Value |
|---|---|---|---|---|---|
| Rec L 14 days | CTRL | 0.6023333 | 0.362705 | 0.8419618 | <.0001* |
| Rec L 14 days | Rec H 14 days | 0.3878889 | 0.148260 | 0.6275174 | 0.0027* |
| Rec H 14 days | CTRL | 0.2144444 | −0.025184 | 0.4540729 | 0.0771 |

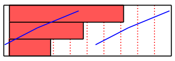

Statistical Table 4.
Statistical analysis of data presented in Figure 2, panel C

**Matched Pairs PCR=a/c, Ig=H3K9m2, Cell=Rec H**

**Difference: +AZA-Basal**

| | | | |
|---|---|---|---|
| +AZA | 0.10156 | t-Ratio | 0.404291 |
| Basal | 0.091 | DF | 8 |
| Mean Difference | 0.01056 | Prob > \|t\| | 0.6966 |
| Std Error | 0.02611 | Prob > t | 0.3483 |
| Upper95% | 0.07076 | Prob < t | 0.6517 |
| Lower95% | -0.0497 | | |
| N | 9 | | |
| Correlation | 0.77192 | | |

**Matched Pairs PCR=a/c, Ig=H3K9m2, Cell=Rec L**

**Difference: +AZA-Basal**

| | | | |
|---|---|---|---|
| +AZA | 0.05256 | t-Ratio | -11.7517 |
| Basal | 3.52233 | DF | 8 |
| Mean Difference | -3.4698 | Prob > \|t\| | <.0001* |
| Std Error | 0.29526 | Prob > t | 1.0000 |
| Upper95% | -2.7889 | Prob < t | <.0001* |
| Lower95% | -4.1506 | | |
| N | 9 | | |
| Correlation | 0.62601 | | |

**Matched Pairs PCR=a/c, Ig=H3K9m3, Cell=Rec H**

**Difference: +AZA-Basal**

| | | | |
|---|---|---|---|
| +AZA | 0.04856 | t-Ratio | -1.39535 |
| Basal | 0.05856 | DF | 8 |
| Mean Difference | -0.01 | Prob > \|t\| | 0.2004 |
| Std Error | 0.00717 | Prob > t | 0.8998 |
| Upper95% | 0.00653 | Prob < t | 0.1002 |
| Lower95% | -0.0265 | | |
| N | 9 | | |
| Correlation | 0.99643 | | |

**Matched Pairs PCR=a/c, Ig=H3K9m3, Cell=Rec L**

**Difference: +AZA-Basal**

| | | | |
|---|---|---|---|
| +AZA | 1.78922 | t-Ratio | -9.29174 |
| Basal | 4.04567 | DF | 8 |
| Mean Difference | -2.2564 | Prob > \|t\| | <.0001* |
| Std Error | 0.24284 | Prob > t | 1.0000 |
| Upper95% | -1.6964 | Prob < t | <.0001* |
| Lower95% | -2.8164 | | |
| N | 9 | | |
| Correlation | 0.80001 | | |

**Matched Pairs PCR=a/c, Ig=Ig-CTRL, Cell=Rec H**

**Difference: +AZA-Basal**

| | | | |
|---|---|---|---|
| +AZA | 0.01056 | t-Ratio | -0.05707 |
| Basal | 0.01067 | DF | 8 |
| Mean Difference | -0.0001 | Prob > \|t\| | 0.9559 |
| Std Error | 0.00195 | Prob > t | 0.5221 |
| Upper95% | 0.00438 | Prob < t | 0.4779 |
| Lower95% | -0.0046 | | |
| N | 9 | | |
| Correlation | -0.4376 | | |

**Matched Pairs PCR=a/c, Ig=Ig-CTRL, Cell=Rec L**

**Difference: +AZA-Basal**

| | | | |
|---|---|---|---|
| +AZA | 0.00911 | t-Ratio | -0.09035 |
| Basal | 0.00922 | DF | 8 |
| Mean Difference | -0.0001 | Prob > \|t\| | 0.9302 |
| Std Error | 0.00123 | Prob > t | 0.5349 |
| Upper95% | 0.00272 | Prob < t | 0.4651 |
| Lower95% | -0.0029 | | |
| N | 9 | | |
| Correlation | 0.43208 | | |

**Matched Pairs PCR=p/q, Ig=H3K9m2, Cell=Rec H**

**Difference: +AZA-Basal**

| | | | |
|---|---|---|---|
| +AZA | 0.29411 | t-Ratio | 0.682803 |
| Basal | 0.28167 | DF | 8 |
| Mean Difference | 0.01244 | Prob > \|t\| | 0.5140 |
| Std Error | 0.01823 | Prob > t | 0.2570 |
| Upper95% | 0.05447 | Prob < t | 0.7430 |
| Lower95% | -0.0296 | | |
| N | 9 | | |
| Correlation | 0.98657 | | |

Statistical Table 4.
Statistical analysis of data presented in Figure 2, panel C

### Matched Pairs PCR=p/q, Ig=H3K9m2, Cell=Rec L

**Difference: +AZA-Basal**

| | | | |
|---|---|---|---|
| +AZA | 0.34089 | t-Ratio | -13.3869 |
| Basal | 3.59022 | DF | 8 |
| Mean Difference | -3.2493 | Prob > \|t\| | <.0001* |
| Std Error | 0.24273 | Prob > t | 1.0000 |
| Upper95% | -2.6896 | Prob < t | <.0001* |
| Lower95% | -3.8091 | | |
| N | 9 | | |
| Correlation | 0.88252 | | |

### Matched Pairs PCR=p/q, Ig=H3K9m3, Cell=Rec H

**Difference: +AZA-Basal**

| | | | |
|---|---|---|---|
| +AZA | 0.91167 | t-Ratio | 8.654662 |
| Basal | 0.734 | DF | 8 |
| Mean Difference | 0.17767 | Prob > \|t\| | <.0001* |
| Std Error | 0.02053 | Prob > t | <.0001* |
| Upper95% | 0.22501 | Prob < t | 1.0000 |
| Lower95% | 0.13033 | | |
| N | 9 | | |
| Correlation | 0.99943 | | |

### Matched Pairs PCR=p/q, Ig=H3K9m3, Cell=Rec L

**Difference: +AZA-Basal**

| | | | |
|---|---|---|---|
| +AZA | 1.97689 | t-Ratio | -12.3681 |
| Basal | 3.94711 | DF | 8 |
| Mean Difference | -1.9702 | Prob > \|t\| | <.0001* |
| Std Error | 0.1593 | Prob > t | 1.0000 |
| Upper95% | -1.6029 | Prob < t | <.0001* |
| Lower95% | -2.3376 | | |
| N | 9 | | |
| Correlation | 0.86646 | | |

### Matched Pairs PCR=p/q, Ig=Ig-CTRL, Cell=Rec H

**Difference: +AZA-Basal**

| | | | |
|---|---|---|---|
| +AZA | 0.01211 | t-Ratio | 1.370315 |
| Basal | 0.01 | DF | 8 |
| Mean Difference | 0.00211 | Prob > \|t\| | 0.2078 |
| Std Error | 0.00154 | Prob > t | 0.1039 |
| Upper95% | 0.00566 | Prob < t | 0.8961 |
| Lower95% | -0.0014 | | |
| N | 9 | | |
| Correlation | -0.3947 | | |

### Matched Pairs PCR=p/q, Ig=Ig-CTRL, Cell=Rec L

**Difference: +AZA-Basal**

| | | | |
|---|---|---|---|
| +AZA | 0.01189 | t-Ratio | 0.862976 |
| Basal | 0.01011 | DF | 8 |
| Mean Difference | 0.00178 | Prob > \|t\| | 0.4133 |
| Std Error | 0.00206 | Prob > t | 0.2066 |
| Upper95% | 0.00653 | Prob < t | 0.7934 |
| Lower95% | -0.003 | | |
| N | 9 | | |
| Correlation | 0.02634 | | |

### Matched Pairs PCR=r/h, Ig=H3K9m2, Cell=Rec H

**Difference: +AZA-Basal**

| | | | |
|---|---|---|---|
| +AZA | 0.05567 | t-Ratio | 1.533677 |
| Basal | 0.01778 | DF | 8 |
| Mean Difference | 0.03789 | Prob > \|t\| | 0.1637 |
| Std Error | 0.0247 | Prob > t | 0.0818 |
| Upper95% | 0.09486 | Prob < t | 0.9182 |
| Lower95% | -0.0191 | | |
| N | 9 | | |
| Correlation | 0.7957 | | |

### Matched Pairs PCR=r/h, Ig=H3K9m2, Cell=Rec L

**Difference: +AZA-Basal**

| | | | |
|---|---|---|---|
| +AZA | 0.07433 | t-Ratio | -8.56859 |
| Basal | 1.69844 | DF | 8 |
| Mean Difference | -1.6241 | Prob > \|t\| | <.0001* |
| Std Error | 0.18954 | Prob > t | 1.0000 |
| Upper95% | -1.187 | Prob < t | <.0001* |
| Lower95% | -2.0612 | | |
| N | 9 | | |
| Correlation | 0.62913 | | |

Statistical Table 4.
Statistical analysis of data presented in Figure 2, panel C

### Matched Pairs PCR=r/h, Ig=H3K9m3, Cell=Rec H

#### Difference: +AZA-Basal

| | | | |
|---|---|---|---|
| +AZA | 2.18767 | t-Ratio | -7.73435 |
| Basal | 2.78011 | DF | 8 |
| Mean Difference | -0.5924 | Prob > \|t\| | <.0001* |
| Std Error | 0.0766 | Prob > t | 1.0000 |
| Upper95% | -0.4158 | Prob < t | <.0001* |
| Lower95% | -0.7691 | | |
| N | 9 | | |
| Correlation | 0.72727 | | |

### Matched Pairs PCR=r/h, Ig=H3K9m3, Cell=Rec L

#### Difference: +AZA-Basal

| | | | |
|---|---|---|---|
| +AZA | 2.70956 | t-Ratio | -26.3437 |
| Basal | 6.75311 | DF | 8 |
| Mean Difference | -4.0436 | Prob > \|t\| | <.0001* |
| Std Error | 0.15349 | Prob > t | 1.0000 |
| Upper95% | -3.6896 | Prob < t | <.0001* |
| Lower95% | -4.3975 | | |
| N | 9 | | |
| Correlation | 0.85039 | | |

### Matched Pairs PCR=r/h, Ig=Ig-CTRL, Cell=Rec H

#### Difference: +AZA-Basal

| | | | |
|---|---|---|---|
| +AZA | 0.01056 | t-Ratio | -0.17933 |
| Basal | 0.011 | DF | 8 |
| Mean Difference | -0.0004 | Prob > \|t\| | 0.8621 |
| Std Error | 0.00248 | Prob > t | 0.5689 |
| Upper95% | 0.00527 | Prob < t | 0.4311 |
| Lower95% | -0.0062 | | |
| N | 9 | | |
| Correlation | -0.4214 | | |

### Matched Pairs PCR=r/h, Ig=Ig-CTRL, Cell=Rec L

#### Difference: +AZA-Basal

| | | | |
|---|---|---|---|
| +AZA | 0.01189 | t-Ratio | 0.8745 |
| Basal | 0.01011 | DF | 8 |
| Mean Difference | 0.00178 | Prob > \|t\| | 0.4073 |
| Std Error | 0.00203 | Prob > t | 0.2037 |
| Upper95% | 0.00647 | Prob < t | 0.7963 |
| Lower95% | -0.0029 | | |
| N | 9 | | |
| Correlation | -0.2743 | | |
